# Rare deleterious mutations of the gene *EFR3A* in autism spectrum disorders

**DOI:** 10.1186/2040-2392-5-31

**Published:** 2014-04-29

**Authors:** Abha R Gupta, Michelle Pirruccello, Feng Cheng, Hyo Jung Kang, Thomas V Fernandez, Jeremy M Baskin, Murim Choi, Li Liu, Adife Gulhan Ercan-Sencicek, John D Murdoch, Lambertus Klei, Benjamin M Neale, Daniel Franjic, Mark J Daly, Richard P Lifton, Pietro De Camilli, Hongyu Zhao, Nenad Šestan, Matthew W State

**Affiliations:** 1 Department of Pediatrics and Child Study Center, Yale School of Medicine, New Haven, CT 06520, USA; 2 Scholar Rock, LLC, 300 Third Street, 4th floor, Cambridge, MA 02142, USA; 3 Department of Neurobiology, Kavli Institute for Neuroscience, Yale School of Medicine, New Haven, CT 06520, USA; 4 College of Pharmacy, University of South Florida, Tampa, FL 33612, USA; 5 Department of Life Science, Chung-Ang University, Seoul, Korea; 6 Department of Psychiatry and Child Study Center, Yale School of Medicine, New Haven, CT 06520, USA; 7 Department of Cell Biology, Howard Hughes Medical Institute, Program in Cellular Neuroscience Neurodegeneration and Repair, Yale School of Medicine, New Haven, CT 06520, USA; 8 Department of Genetics, Howard Hughes Medical Institute, Yale School of Medicine, New Haven, CT 06520, USA; 9 Department of Statistics, Carnegie Mellon University, Pittsburgh, PA 15213, USA; 10 Program on Neurogenetics, Child Study Center, Yale School of Medicine, New Haven, CT 06520, USA; 11 Program on Neurogenetics, Child Study Center, Department of Psychiatry, Department of Genetics, Yale School of Medicine, New Haven, CT 06520, USA; 12 Department of Psychiatry, University of Pittsburgh School of Medicine, Pittsburgh, PA 15213, USA; 13 Program in Medical and Population Genetics, Broad Institute of Harvard and MIT, Cambridge, MA 02142, USA; 14 Departments of Biostatistics and Genetics, Yale School of Medicine, New Haven, CT 06520, USA; 15 Department of Psychiatry, University of California San Francisco, San Francisco, CA 94143, USA

**Keywords:** Autism spectrum disorder, Genetics, Rare variants, *EFR3A*, Synapse, Phosphoinositide metabolism

## Abstract

**Background:**

Whole-exome sequencing studies in autism spectrum disorder (ASD) have identified *de novo* mutations in novel candidate genes, including the synaptic gene *Eighty-five Requiring 3A (EFR3A)*. EFR3A is a critical component of a protein complex required for the synthesis of the phosphoinositide PtdIns4P, which has a variety of functions at the neural synapse. We hypothesized that deleterious mutations in *EFR3A* would be significantly associated with ASD.

**Methods:**

We conducted a large case/control association study by deep resequencing and analysis of whole-exome data for coding and splice site variants in *EFR3A*. We determined the potential impact of these variants on protein structure and function by a variety of conservation measures and analysis of the *Saccharomyces cerevisiae* Efr3 crystal structure. We also analyzed the expression pattern of *EFR3A* in human brain tissue.

**Results:**

Rare nonsynonymous mutations in *EFR3A* were more common among cases (16 / 2,196 = 0.73%) than matched controls (12 / 3,389 = 0.35%) and were statistically more common at conserved nucleotides based on an experiment-wide significance threshold (*P* = 0.0077, permutation test). Crystal structure analysis revealed that mutations likely to be deleterious were also statistically more common in cases than controls (*P* = 0.017, Fisher exact test). Furthermore, *EFR3A* is expressed in cortical neurons, including pyramidal neurons, during human fetal brain development in a pattern consistent with ASD-related genes, and it is strongly co-expressed (*P* < 2.2 × 10^−16^, Wilcoxon test) with a module of genes significantly associated with ASD.

**Conclusions:**

Rare deleterious mutations in *EFR3A* were found to be associated with ASD using an experiment-wide significance threshold. Synaptic phosphoinositide metabolism has been strongly implicated in syndromic forms of ASD. These data for *EFR3A* strengthen the evidence for the involvement of this pathway in idiopathic autism.

## Background

Autism spectrum disorders (ASDs) are defined by persistent deficits in social communication and social interaction and restricted repetitive patterns of behavior, interests or activities [1]. These syndromes are common in the population, with a prevalence of approximately 1% [2], and demonstrate both considerable phenotypic and extensive genetic heterogeneity [3]. High-throughput sequencing approaches have provided substantial insight into the genomic architecture of ASDs. For example, multiple analyses of whole-exome sequencing data demonstrate an over-representation of *de novo*, loss-of-function mutations in brain-expressed genes in affected individuals and point to half a dozen new ASD genes [3-6]. These have been identified based on the clustering of mutations in the same gene in unrelated individuals, providing strong evidence for association [3]. However, a large number of compelling, rare, *de novo* missense mutations are also found in probands, though a clear threshold for identifying the association of these mutations with ASD is less obvious. Both the rarity of the individual mutations and the small size of current exome discovery cohorts suggest that clarifying which of these *de novo* mutations point to *bona fide* ASD genes will require alternative approaches. Large-scale, targeted, case/control sequencing as a complement to *de novo* mutation discovery in ASD is one such strategy.

In a whole-exome analysis of 238 families [3], we identified a single proband carrying two novel *de novo* missense mutations in synaptic genes, one each in *EFR3A* (*Eighty-five Requiring 3A* [NCBI Reference Sequence: NM_015137]) and *CASK* (*Calcium/Calmodulin-dependent Serine/Threonine Kinase* [NCBI Reference Sequence: NM_003688]). Both yeast *EFR3*[7] and mammalian *EFR3A* and *EFR3B*[8] have been linked to the control of phosphoinositide metabolism, a pathway demonstrated to play a role in ASD [9]. *CASK* is implicated in X-linked intellectual disability [10]. Neither the occurrence of one or several *de novo* missense mutations in a single affected individual is a statistically significant finding. However, our overall analysis of 599 simplex ASD quartets suggests that approximately 20% of *de novo* missense mutations in brain-expressed genes found in cases will prove to be true ASD loci, representing an approximately fourfold increase over a brain-expressed gene chosen at random [11]. Given an increased prior probability based on the exome results and the strong biological plausibility of both genes, we conducted a targeted analysis of *EFR3A* and *CASK* in large cohorts using both Sanger sequencing and whole-exome data. We found that rare deleterious mutations in *EFR3A* are associated with ASD using an experiment-wide significance threshold.

## Methods

### Subjects

Initial cases were drawn from the Simons Simplex Collection (SSC). The SSC is an exhaustively characterized ASD family cohort, with the majority of families consisting of a proband, two unaffected parents and an unaffected sibling. The diagnostic methodology used is well described elsewhere [12]. *EFR3A* and *CASK* were sequenced in 705 cases of European ancestry (Family Distribution List v13) based on genome-wide genotyping data (see below). Based on a preliminary analysis of the sequence data, mutation screening then focused on *EFR3A*, which was evaluated in several cohorts for which we had access to DNA or whole-exome sequencing results. Additional cases were drawn from the SSC (*n* = 452) and via collaboration with the ARRA Autism Sequencing Collaboration (AASC, *n* = 1,039). All cases were identified as having European ancestry via genome-wide genotyping data. Sample characteristics and diagnostic methodology for the AASC have been described previously [5,13]. For controls, 912 were drawn from the National Institute of Neurological Disorders and Stroke (NINDS) Neurologically Normal Caucasian Control Panel (NDPT020, 079, 082, 084, 090, 093, 094, 095, 096, and 098). This set of adult subjects has a negative personal and family history (first-degree relatives) of neuropsychiatric illness. Additional controls were drawn from the AASC (*n* = 863) and from ongoing studies of non-neuropsychiatric conditions at our home institution (northern European (NE) controls, *n* = 1,614). Again, all controls were of confirmed European ancestry. The NE and AASC controls were considered population controls since subjects with potential neuropsychiatric disorders were not excluded. This study only accessed de-identified biospecimens or sequencing data and no protected health information; it received an exemption from human subject research from the Yale Human Research Protection Program.

### Genotyping and ancestry matching

1,304 SSC cases were genotyped using Human1M-Duo v1, Human1M-Duo v3 or HumanOmni2.5 BeadChips (Illumina, San Diego, CA, USA). 923 NINDS controls were genotyped using Illumina HumanOmniExpress12v1. 1,779 NE controls were genotyped using Illumina 550 K Single or 610 Quad v1 BeadChips. Subjects were removed because of: (1) genotyping call rate <95%, (2) discrepancy of genotyping data with recorded sex, and (3) Mendelian inconsistencies or cryptic relatedness (up to and including second-degree relatives).

For ancestry matching, Golden Helix SNP and Variation Suite v7.5.4 (Bozeman, MT, USA) was used in principal component analysis (PCA) of SSC cases, NINDS controls and NE controls using 8,210 SNPs common to all arrays and not in high linkage disequilibrium. Based on visualization of a scree plot (Additional file 1: Figure S1), eigenvalues of the first three principal components, which contributed the greatest amount of variation relative to the other principal components, were plotted against one another (Additional file 2: Figure S2). The interquartile range (IQR) distance around the median of the study population cluster was calculated. A threshold that included all NINDS and NE controls was determined to lie at 5 IQRs from the third quartile, and 54 SSC cases beyond this threshold were excluded as ancestral outliers (Additional file 3: Figure S3). The final cohort sizes were 1,157 SSC cases, 912 NINDS controls and 1,614 NE controls.

AASC cases and controls were genotyped using Illumina microarrays, including 550 K, 610 K and 1 M BeadChips, and also filtered to exclude subjects because of: (1) genotyping call rate <95%, (2) discrepancy of genotyping data with recorded sex, and (3) Mendelian inconsistencies or cryptic relatedness. Ancestry matching between AASC cases and controls was conducted using PCA of genotyping data for a subset of SNPs common to all arrays; each case was matched to the nearest control using a greedy algorithm. The final cohort sizes were 1,039 cases and 863 controls, all of European descent.

### Sanger sequencing of Simons Simplex Collection cases and NINDS controls

PCR primers were designed to flank all coding exons and splice sites of *EFR3A* and *CASK* (Additional file 4: Table S1). Then 10 ng lymphoblastoid cell line-derived genomic DNA served as template in a 25 μl PCR containing 1× PreMix D buffer (Epicentre Biotechnologies, Madison, WI, USA), 0.48 μM each forward and reverse primer, and 0.36 μL Taq polymerase/0.072 μL Pyrococcus furiosus (PFU) polymerase. Both enzymes, which were synthesized in house, were used to permit proofreading during PCR and reduce Taq-induced mutations. A Tetrad2 Peltier Thermal Cycler (Bio-Rad, Hercules, CA, USA) was programmed as follows: 95.0°C/5 min; 40 cycles of 95.0°C/30 sec, 60.0°C/30 sec and 72.0°C/60 sec; 72.0°C/10 min. PCR products were visualized by agarose gel electrophoresis and sent to Beckman Coulter Genomics (Danvers, MA, USA) or the Yale Keck Biotechnology Resource Laboratory (New Haven, CT, USA) for Sanger sequencing. Chromatograms were aligned and analyzed using Sequencher v4.9 (Gene Codes, Ann Arbor, MI, USA). We obtained a 96% sequencing success rate for both cases and controls. All potential rare (<1% frequency) nonsynonymous variants were confirmed by a second round of PCR and Sanger sequencing in forward and reverse directions, using blood-derived genomic DNA for SSC cases since it was available. Segregation analysis of confirmed variants was performed using blood-derived genomic DNA from all family members, which were only available for SSC cases.

### Whole-exome data from northern European controls and ARRA Autism Sequencing Collaboration cases/controls

For the NE controls, we examined whole-exome sequencing data. Greater than 98% of the *EFR3A* coding and splice site sequence was covered by at least eight independent reads. For variant calling, a minimum read threshold of only one independent read was used to minimize the liability for false negatives. All coding and splice site variants with a SAMtools SNP quality score ≥50 were subjected to confirmation by PCR and Sanger sequencing of whole-genome amplified DNA.

We also examined whole-exome sequencing data for AASC cases and controls (generated by the Broad Institute and Baylor College of Medicine), which were treated as a matched set and subjected to identical quality control and variant calling criteria within each site. All coding and splice site variants were identified after three rounds of filtering the whole-exome data for quality control. Variants were excluded if: (1) they had ≥10% missing calls, (2) they had average coverage <17 for Broad cases/controls and <12 for Baylor cases/controls, and (3) >50% of minor allele calls had <17 reads or a balance of depth >0.66 for Broad cases/controls and <12 reads or a balance of depth >0.75 for Baylor cases/controls (balance of depth being defined as the number of reference reads divided by the total number of reads). Filtering criteria differed between the two sites since samples were sequenced on different platforms and the data were processed using different software packages (Illumina/GATK at Broad and Solid/AtlasSNP2 at Baylor). AASC case and control variants were not confirmed by Sanger sequencing. However, given that the cohorts are approximately the same size and the entire AASC set was subjected to identical sequencing methods, we anticipated that calling errors would be randomly distributed across affected and unaffected individuals.

To assess the novel singleton status of variants identified in all case and control groups, we queried dbSNP137 and whole-exome sequencing data from an additional 6,503 individuals from release ESP6500 of the Exome Variant Server, comprising 4,300 European-Americans and 2,203 African-Americans.

### Analysis by conservation measures

We evaluated conservation at the positions of novel nonsynonymous singleton mutations in *EFR3A* with three widely used informatics tools: PhyloP (phylogenetic *P* values), GERP (genomic evolutionary rate profiling) and ConSurf. PhyloP scores [14] were obtained from the UCSC Genome Browser. A PhyloP score ≥1.3 indicates *P* = 0.05 for conservation and was used as a threshold to determine whether a mutation occurred at a conserved site. GERP scores [15] were obtained from the SeattleSeq annotation pipeline. A GERP score ≥5 was used as a threshold to determine conservation [16]. Regarding ConSurf analysis, a multiple EFR3 protein sequence alignment was constructed using PSI-Blast, which was then edited to remove partial or redundant sequences and produce a comprehensive sampling of genetic space. Both EFR3A and EFR3B were included to increase the number of sequences available, which totaled 42 (Additional file 5: Figure S4). The alignment was produced with TCoffee and sent to the ConSurf server to quantify conservation [17]. The server normalizes the conservation score for each amino acid such that average positions cluster around zero; the most conserved residues have negative scores, and the least conserved are positive.

### Structure-based analysis

The crystal structure of the N-terminal fragment (amino acids 8 to 562) of the *Saccharomyces cerevisiae* Efr3 was recently determined [18]. Yeast Efr3 and human EFR3A were aligned through amino acid 451, corresponding to the most conserved portion of Efr3 (Additional file 6: Figure S5). We created a homology model and found that secondary structure predictions of the human EFR3A matched well with the observed secondary structure of the yeast protein. Based on the crystal structure, human *EFR3A* case and control mutations were blindly assessed for their potential to disrupt protein structure and function using the following structural criteria prioritization: first, it was determined whether the mutated residue was located in the protein core or on the surface as shown by the crystal. If the mutation was located in the core, it was then assessed, taking secondary structure into account, for whether a hydrophilic residue would be placed in a hydrophobic environment or whether the mutation changed the residue size, which could result in a defect in packing the core or misfolding. If the mutation was located on the surface of the protein, it was then determined whether that area was well conserved and hence likely to be functionally important. If so, any change in residue charge and/or size was categorized as potentially disruptive as these could affect protein-protein or protein-membrane interactions. To this end we devised a grading scheme, where deleterious variants received a score of 3 or higher. It should be noted, however, that this grading scheme cannot take into account interactions of EFR3A that have not been described to date.

### In situ *hybridization*

Human brain tissue samples were fixed in 4% PFA (Paraformaldehyde) at 4°C for 2 to 3 days, cryoprotected in graded sucrose solutions (up to 30%) at 4°C, frozen at −40°C in isopentane/dry ice, and stored at −80°C. Frozen samples were cut at 20 μm using a Leica CM3050S cryostat and mounted onto gelatine-coated slides. To prepare complementary RNA probes, cDNA was amplified with T7 and SP6 promoter-attached primers (T7/forward primer: TAATACGACTCACTATAGGGAGACGGGCCACCATTTGGGAACCT, SP6/reverse primer: GCGATTTAGGTGACACTATAGCCAGCACTGTCGGACCTATGGA) and used to generate digoxigenin-labeled riboprobes with T7 RNA polymerase (Roche, Basel, Switzerland) for the sense probe (negative control) and SP6 RNA polymerase (Roche) for the antisense probe. After acetylation, sections were hybridized with the riboprobes at 55°C/16 hr. They were then processed as follows: (1) rinsed in 2× SSC, (2) incubated with 20 μg/ml RNase A at 37°C/30 min, (3) washed in high stringency conditions at 60°C, (4) incubated at room temperature (RT)/2 hr with AP-coupled anti-digoxigenin Fab fragment (Roche) in 1% donkey serum in TBST, and (5) washed in NTMT buffer (2 × 10 min). Signals were developed in a light-protected humidified chamber with NBT/BCIP in NTMT buffer/2 mM levamisole solution at RT overnight. The sections were rinsed in TE and cover-slipped using a crystal aqueous mounting medium (Accurate Chemical and Scientific Corporation, Westbury, NY, USA). SSC: saline-sodium citrate buffer, AP: alkaline phosphatase, TBST: Tris-buffered saline, 0.1% Tween-20, NTMT: NaCl + Tris-HCl + Magnesium chloride + Tween-20, NBT: nitro-blue tetrazolium, BCIP: 5-bromo-4-chloro-3’-indolyphosphate, TE: Tris-EDTA buffer.

### Gene co-expression analysis

Using data from Kang *et al.*[19], Spearman correlation was performed between expression levels of *EFR3A* and M12 genes [20] and between *EFR3A* and all 15,132 genes expressed in the human brain [19]. Of the 432 unique genes in M12, 356 had expression data in the array platform used by Kang *et al.*[19], and these were used to perform the analysis (Additional file 7: Table S2). These analyses were also performed with ten additional genes: *ACTB* (a housekeeping gene), *CHD8*, *DYRK1A*, *EFR3B* (a homologue), *GRIN2B*, *KATNAL2*, *NRXN1*, *SCN2A*, *SHANK2* and *SHANK3*. The median expression correlation coefficients for the 11 genes when compared to M12 and all brain-expressed genes are shown in Additional file 8: Table S3. To show the distribution of the correlation coefficients, kernel density plots were generated using the sm.density.compare function in the sm package in R with the smoothing parameter *h* = 0.1. The entire process was repeated for an additional three modules: M2, M3 and M16 genes [21].

### Statistical analysis

All *P* values for mutation burden, conservation measures and crystal structure analysis were calculated by the Fisher exact test. We used the right-tailed test based on the hypothesis that there would be a greater number of mutations in cases versus controls and that case mutations would be more deleterious. Since we initially investigated two genes, *CASK* as well as *EFR3A*, we performed a Bonferroni correction and multiplied the *P* value for overall mutation burden by two. The initial *de novo* mutation F338S identified by whole-exome sequencing was not included in calculations of overall mutation burden between cases and controls but was included when assessing the potential deleteriousness of case versus control mutations.

To study the relative enrichment of variants at conserved positions in cases and controls, we conducted the following analysis. For each novel nonsynonymous singleton variant, we used cutoff values for three conservation measures to annotate whether each variant maps to a conserved position and is, therefore, potentially deleterious: PhyloP ≥ 1.3 (indicates *P* = 0.05 for conservation), GERP ≥ 5 [16] and ConSurf < 0 (indicates conservation). We also performed a permutation test by first creating an input file (Additional file 9: Table S4) of binary entries, with ‘1’ indicating that the variant met the cutoff and is functional by that conservation measure and ‘0’ indicating that it did not. For each measure, we calculated the proportion of individuals carrying functional variants in the case and control cohorts. We then calculated the ratio of the two proportions as the relative enrichment in cases. We used the largest ratio among the three measures as the test statistic for the observed data. To estimate the statistical significance, we adopted the following permutation procedure. For each of 10,000 permutations, we permuted the case and control labels of the subjects. Based on the case and control groups defined by the permuted labels, we repeated the same relative enrichment ratio calculation and estimated a *P* value for enrichment of deleterious mutations in cases. The nonparametric Wilcoxon test was used to calculate the *P* value for the difference in median expression correlation coefficients between *EFR3A*/M12 and *EFR3A*/all brain-expressed genes.

### Western blot analysis of mouse tissues

Female C57BL/6 mice (Jackson Laboratory, Bar Harbor, ME, USA) were euthanized at approximately 21 days after birth. Organs were isolated, homogenized in lysis buffer (1% Triton X-100, 150 mM NaCl, 20 mM Tris, 0.5 mM EDTA (Ethylenediaminetetraacetic acid), pH 7.4, supplemented with Complete EDTA-free protease inhibitor tablet (Roche)), centrifuged for 10 min at 16,000 *g*, and the supernatant was reserved. Protein concentrations were normalized using the bicinchoninic acid (BCA) assay (Thermo Pierce, Rockford, IL, USA). The samples were analyzed by Western blot (30 μg sample/lane), probing with anti-EFR3A (Ab2, Sigma, St Louis, MO, USA) or anti-GAPDH (1D4, GeneTex, Irvine, CA, USA) antibodies. For the EFR3A Western blot, a goat HRP (horseradish peroxidase)-conjugated anti-rabbit secondary antibody (Bio-Rad, Hercules, CA, USA) was used, and the blot was developed using SuperSignal West Pico chemiluminescent reagent (Thermo Pierce). For the GAPDH Western blot, an IRDye 800CW-conjugated anti-mouse secondary antibody (LI-COR Biosciences, Lincoln, NE, USA) was used, and the Western blot was scanned on an Odyssey imaging system (LI-COR Biosciences).

### Verification of EFR3A antibody specificity

The specificity of the EFR3A antibody (Ab2, Sigma) was verified by Western blot analysis of HeLa cell lysates treated with siRNA duplexes (Integrated DNA Technologies, Coralville, IA, USA) targeted against human *EFR3A* or negative control siRNA, termed NC1. The siRNA sequences are shown in Additional file 10: Table S5. HeLa cells were transfected with the appropriate siRNA duplex (from a 20 μM stock in 30 mM HEPES (4-(2-Hydroxyethyl)piperazine-1-ethanesulfonic acid, N-(2-Hydroxyethyl)piperazine-N´-(2-ethanesulfonic acid)), 100 mM potassium acetate) using RNAiMAX (Invitrogen, Carlsbad, CA, USA) according to the manufacturer’s instructions, and after 6 hr, the media was exchanged for regular growth media. After 3 days, the cells were collected, dissolved in lysis buffer (1% Triton X-100, 150 mM NaCl, 20 mM Tris, 0.5 mM EDTA, pH 7.4, supplemented with Complete EDTA-free protease inhibitor tablet (Roche)), centrifuged for 10 min at 16,000 *g*, and the supernatant was reserved. Protein concentrations were normalized using the BCA assay (Thermo Pierce). The samples were analyzed by Western blot (50 μg sample/lane), probing with anti-EFR3A (Ab2, Sigma) or anti-α-tubulin (B-5-1-2, Sigma) antibodies. IRDye 800CW-conjugated anti-rabbit and anti-mouse secondary antibodies (LI-COR Biosciences) were used, and the Western blots were scanned on an Odyssey imaging system (LI-COR Biosciences).

## Results

To test our hypothesis that mutations in *EFR3A* and/or *CASK* confer risk for ASD, we performed Sanger sequencing of all coding exons and splice sites of both genes in 705 comprehensively phenotyped European cases from the SSC. All rare (<1% frequency) nonsynonymous variants were confirmed by a second round of Sanger sequencing. We focused on novel alleles seen only once (singleton variants) and not present in two large databases, dbSNP137 and Exome Variant Server, the latter of which had 6,503 exomes. We reasoned that this strategy would most likely identify deleterious substitutions subject to purifying selection and provide, along with case/control matching for ancestry, the most robust protection against population stratification [22].

In *CASK*, only two variants met these criteria among all 705 cases (Additional file 11: Table S6). In light of the low cumulative allele frequency and anticipated low power to detect an effect, we did not pursue this gene further. We identified six novel nonsynonymous singleton mutations in *EFR3A* (Table 1 and Additional file 12: Table S7) and, consequently, we proceeded to screen this gene in several cohorts for which we had access to DNA or whole-exome sequencing data. We identified an additional 1,491 European cases: (1) 452 from the SSC were subjected to Sanger sequencing and (2) 1,039 from the AASC had whole-exome sequencing data, for a total of 2,196 cases. We identified a total of 3,389 European controls: (1) 912 NINDS neurologically normal European controls matched to SSC cases via PCA of genotyping data and subjected to Sanger sequencing, (2) 1,614 neuropsychiatrically unscreened controls of NE origin matched to SSC cases and who had whole-exome sequencing data, and (3) 863 from the AASC with whole-exome sequencing data. For the NE control exomes, a minimum read threshold of only one independent read was used to identify variants in an effort to minimize false negatives. For the AASC dataset, Broad cases/controls and Baylor cases/controls were matched and evaluated using identical variant-calling approaches and filtering criteria within each site. All rare nonsynonymous variants in SSC cases, NINDS controls and NE controls were confirmed by Sanger sequencing; confirmations were not available for case and control AASC variants.

**Table 1:** **Novel nonsynonymous singleton mutations in *EFR3A***

**Cohort**	**Exon**	**Amino acid change**^**a**^	**Chromosome**	**hg19**	**Reference**	**Variant**	**ID**	**Sex**	**Father**	**Mother**	**Sib**	**PhyloP**	**GERP**	**ConSurf**	**Crystal**
Cases (*n* = 2,196)	**SSC cases (*n*** **= 705)**														
	3	R70C	8	132957112	C	T	12093.p1	M	+	-	n/a	2.95617	5.73	−1.51	Deleterious
	4	L118P	8	132958867	T	C	12610.p1	M	+	-	- F	3.21686	5.47	−0.73	Deleterious
	7	G243A	8	132968104	G	C	11572.p1	M	-	+	+ F	6.387	5.64	−0.291	Benign
	10	F338S^b^	8	132982744	T	C	11379.p1	F	-	-	- F	4.58456	5.54	−1.107	Deleterious
	15	I534T	8	132996411	T	C	11473.p1	M	-	+	+ F	4.78917	6.02	−0.113	n/a
	15	I576_A577insI^c^	8	132996539	*	I:ATT	11577.p1	M	-	+	+ F	3.628	5.03	0.043	n/a
	22	T785A	8	133015525	A	G	11808.p1	F	-	+	- F	3.02491	4.27	−0.312	n/a
	**SSC cases (*n*** **= 452)**														
	5	F123L	8	132962216	T	C	13507.p1	M	-	+	- M	4.86423	5.51	−1.377	Deleterious
	7	G216Sfs*12^d^	8	132968022	*	I:TCGCATA	11027.p1	M	-	+	- M	6.995	6.07	−1.591	Deleterious
	**AASC cases (*n*** **= 1039)**														
	3	K50E	8	132957052	A	G	20094	M	n/a			5.32367	5.55	−1.59	Deleterious
	9	A321S	8	132980647	G	T	00HI1409C	M	n/a			3.10583	5.51	−0.66	Benign
	10	V337L	8	132982740	G	C	00HI1533A	M	n/a			6.32607	5.54	−0.986	Benign
	14	D504G	8	132991604	A	G	06C57233	M	n/a			4.41785	5.06	−0.002	n/a
	14	L508P	8	132991616	T	C	07C71126	M	n/a			4.19838	5.06	0.349	n/a
	14	I510V	8	132991621	A	G	20072	M	n/a			2.07681	3.88	0.088	n/a
	15	Q528R	8	132996393	A	G	98HI0204A	M	n/a			5.21937	6.02	0.08	n/a
	17	M646V	8	132998507	A	G	08C73985A	M	n/a			2.13232	4.65	2.129	n/a
Controls (*n* = 3,389)	**NINDS controls (*n*** **= 912)**														
	2	P14R	8	132952776	C	G	ND11540	M	n/a			5.86267	5.93	−1.264	Benign
	**NE controls (*n*** **= 1,614)**														
	5	R161*^d^	8	132962330	C	T	S19G8	M	n/a			6.995	6.07	−1.591	Deleterious
	6	M194V	8	132966156	A	G	S16H7	F	n/a			5.34254	5.73	−0.985	Benign
	9	E320D	8	132980646	G	T	S6G2	F	n/a			1.06245	2.3	−0.162	Benign
	10	N354D	8	132982791	A	G	S6B11	M	n/a			0.272244	0.403	1.188	Benign
	13	T451M	8	132991119	C	T	S4H5	M	n/a			2.72643	5.71	0.289	Benign
	15	R532W	8	132996404	C	T	S1B3	F	n/a			0.659283	0.794	1.218	n/a
	15	D570G	8	132996519	A	G	S15E2	F	n/a			5.21937	6.02	−0.169	n/a
	**AASC controls (*n*** **= 863)**														
	3	G55C	8	132957067	G	T	04C27095A	F	n/a			6.81431	5.73	−1.321	Benign
	4	F100L	8	132958812	T	C	05C42103	M	n/a			4.82034	5.47	−0.879	Benign
	8	D268G	8	132971858	A	G	05C42750	F	n/a			4.99605	5.64	0.396	Benign
	15_16	? (5′ splice site)^d^	8	132996549	T	C	05C45515	F	n/a			6.995	6.07	−1.591	n/a

^a^All mutations are single alleles, i.e., heterozygous.

^b^F338S is the initial *de novo* mutation identified by whole-exome sequencing.

^c^For I576_A577insI, PhyloP, GERP and ConSurf scores were derived by averaging the scores of the adjacent positions.

^d^These mutations were assigned the maximum conservation scores found for any position in *EFR3A*.

*Under the Reference column indicates absence of the insertion/deletion variant.

AASC, ARRA Autism Sequencing Collaboration; F, female; GERP, genomic evolutionary rate profiling; M, male; n/a, not applicable; NE, northern European; NINDS, National Institute of Neurological Disorders and Stroke; PhyloP, phylogenetic *P* values; SSC, Simons Simplex Collection.

The analysis of a total of 2,196 cases and 3,389 controls demonstrated that novel nonsynonymous singleton mutations in *EFR3A* were twice as frequent in cases compared to controls. However, the *P* value was not statistically significant when corrected for the investigation of two genes since we initially analyzed *CASK* as well as *EFR3A* (16/2,196 cases and 12/3,389 controls; *P* = 0.084, odds ratio = 2.065, 95% confidence interval = 0.924 to 4.652, Fisher exact test, right-tailed). Since the combination of low allele frequency and high conservation has been shown to provide high sensitivity and specificity for predicting functionality in rare variant studies, in contrast to *in silico* prediction programs [23], we evaluated conservation with three widely used informatics tools: PhyloP, GERP and ConSurf. All found significantly more case variants mapping to conserved positions (Tables 1 and 2). Furthermore, using 10,000 permutations of the cases and controls to test the significance of the enrichment of deleterious mutations in cases, we calculated a *P* value of 0.0077. We evaluated family data from SSC subjects and found that all newly identified variants were transmitted. Whole-exome data for only two of these subjects (11379.p1 and 11808.p1) have now been reported; neither has a *de novo* loss-of-function mutation which might contribute to their phenotype. SSC case 11808.p1 does have a novel *de novo* missense mutation (N160S) in *DGCR14* (*DiGeorge Syndrome Critical Region Gene 14*), which has not been associated with ASD or intellectual disability [24]. We also determined that all of the SSC subjects except one (13507.p1) have undergone genome-wide copy number variant (CNV) analysis; none have *de novo* CNVs that might better explain their phenotype [25].

**Table 2:** **Statistics for novel nonsynonymous singleton mutations in *EFR3A***

**Cohorts ( *n *)**		**PhyloP ≥ 1.3**^**b**^	**GERP ≥ 5**^**c**^	**ConSurf < 0**^**d**^	**Deleterious by crystal**
Cases (2,196)	Number of mutations^a^	17 (0.77%)	14 (0.64%)	12 (0.55%)	6 (0.27%)
Controls (3,389)	Number of mutations	9 (0.27%)	9 (0.27%)	8 (0.24%)	1 (0.03%)
	*P*^e^	**0.006**	**0.030**	**0.049**	**0.017**
	Odds ratio	2.930	2.410	2.322	9.282
	95% confidence interval	1.234–7.109	0.979–6.032	0.885–6.214	1.119–204.784

^a^The initial *de novo* mutation was not included in calculations of mutation burden but was in calculations involving severity.

^b^PhyloP ≥ 1.3 indicates *P* = 0.05 for conservation.

^c^GERP ≥ 5 [16].

^d^ConSurf < 0 indicates conservation.

^e^All *P* values are calculated by Fisher exact test, right-tailed. *P* ≤ 0.05 are in bold.

GERP, genomic evolutionary rate profiling; PhyloP, phylogenetic *P* values.

**Figure 1:**
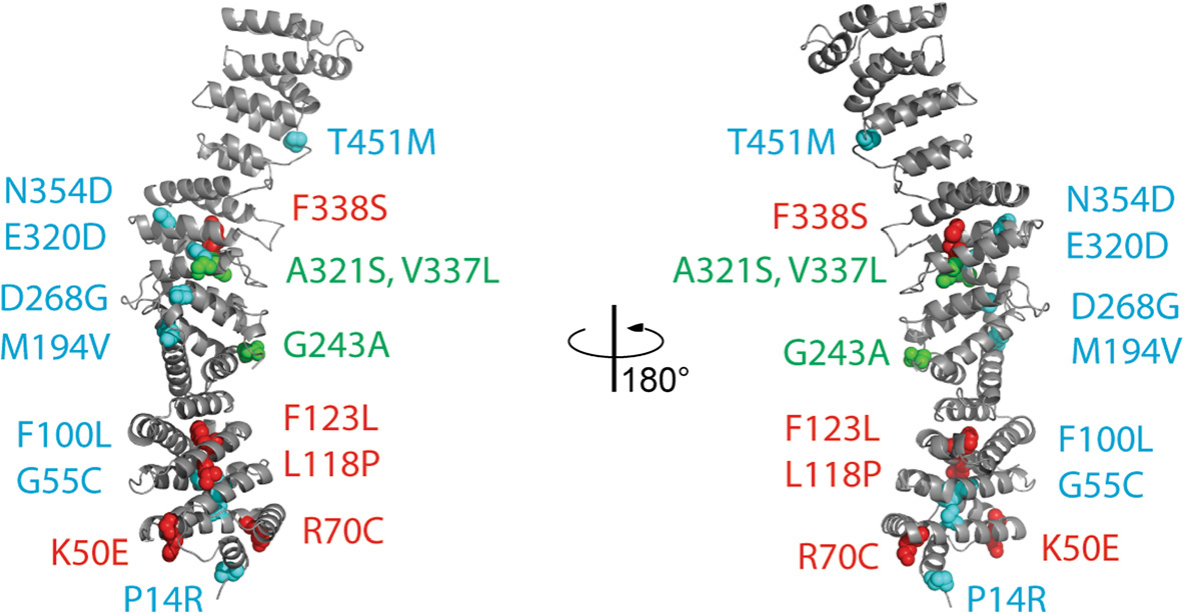
**Ribbon diagram of *S. cerevisiae *Efr3 crystal structure.** Crystallization of Efr3 revealed a series of HEAT repeats, as we had predicted bioinformatically. Alignment of yeast Efr3 and human EFR3A was reliable to amino acid 451. Blinded to case/control status, the human mutations were mapped and analyzed for their potential to disrupt protein structure and function given the three-dimensional crystal structure. Mutations in red are deleterious and found in cases. (R161* in a control and G216Sfs*12 in a case are not shown but presumed to be deleterious.) Mutations in green are benign and found in cases. Mutations in blue are benign and found in controls.

We took advantage of the recently available crystal structure (Protein Data Bank ID 4N5A) of the N-terminal fragment of *S. cerevisiae* Efr3 [18] to map the human mutations (Figure 1) and determine their potential to disrupt protein structure and function, blinded to case/control status. Because the C-terminal portion is not well conserved, only mutations up to and including amino acid 451 could be evaluated with high confidence. (The full length EFR3A protein has 821 amino acid residues.) Every mutation that was assessed to be deleterious as informed by the crystal structure was a case variant, except R161*, which is assumed to be damaging (Table 1 and Additional file 13: Table S8). R161* was found in an NE control for whom neuropsychiatric information is unavailable, so we cannot determine if this mutation is associated with any neuropsychiatric condition. Thus, crystal structure analysis also identifies a significantly greater number of deleterious mutations in cases than controls (*P* = 0.017, odds ratio = 9.282, 95% confidence interval = 1.119 to 204.784, Table 2). As would be expected, all of these deleterious mutations are also at highly conserved positions as per PhyloP, GERP and ConSurf. Interestingly, the reverse is not always true, i.e., there are mutations at highly conserved positions which were assessed to be benign in light of the crystal structure. Therefore, having knowledge of the three-dimensional structure of Efr3 enriches our analysis by providing more biological information to evaluate the deleteriousness of mutations. We also noted for subjects with family data, deleterious mutations as per the crystal structure are not shared by the unaffected siblings (Table 1).

*EFR3A* is a member of the *EFR3* family of genes, conserved throughout eukaryotes and essential for viability [7]. The *Drosophila melanogaster* homologue, rolling blackout (RBO), is highly expressed in the nervous system [26], is enriched at the neural synapse [27], and was proposed to regulate phospholipase C signaling [26]. RBO has also been proposed to function as a transmembrane lipase [26], but structural analysis of Efr3 does not support this hypothesis (Additional file 14: Table S9) [18]. Instead, it shows that EFR3/RBO has a scaffold function with the majority of the protein comprising alpha-helical HEAT (Huntington, Elongation factor 3, regulatory subunit A of protein phosphatase 2A, and Target of rapamycin) repeats.

**Figure 2:**
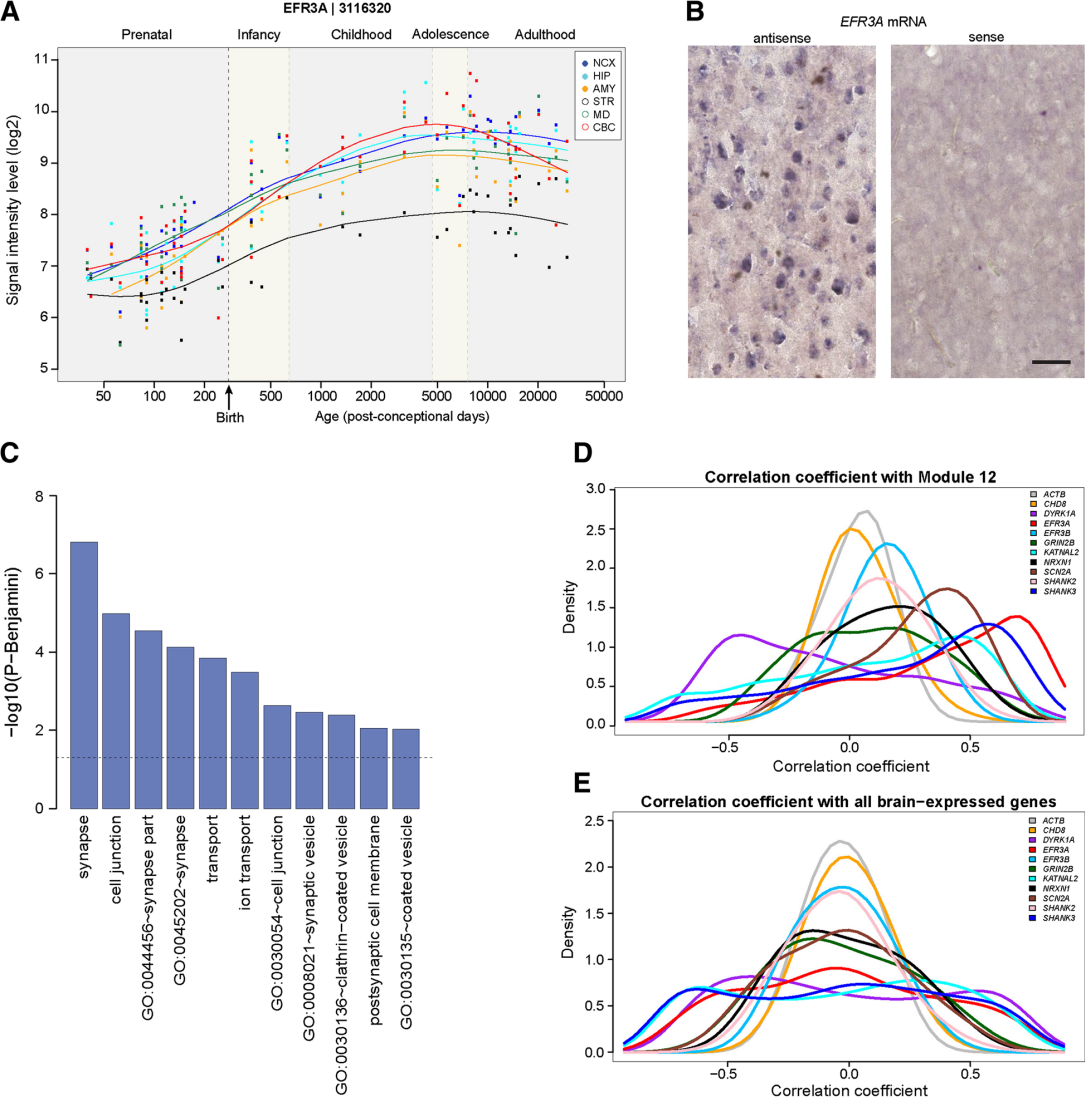
**Expression analysis of human *EFR3A*. (A)** Spatio-temporal mRNA expression of *EFR3A* in the human brain. Line plots show log_2_-transformed exon-array signal intensity during prenatal to adult stages. **(B)***In situ* hybridization of *EFR3A*, using antisense and sense (negative control) probes, in the dorsolateral prefrontal cortex of 40-year-old human brain. Scale bar, 20 μm. **(C)** Functional annotation of top 100 genes correlated with *EFR3A* expression. The dashed line is the threshold for significance, *P* = 0.05. **(D)** Distribution of expression correlation coefficients of *EFR3A* and ASD genes with M12 genes (*n* = 356) [20]. **(E)** Distribution of expression correlation coefficients of *EFR3A* and ASD genes with all brain-expressed genes (*n* = 15,132) [19]. The homologue *EFR3B* is shown for comparison and *ACTB*, a housekeeping gene, is included as a negative control.

The tissue expression of *EFR3A* has not been described, so we performed Western blot analysis of several mouse tissues and found that *EFR3A* is broadly expressed, including in the brain (Additional file 15: Figure S6). We also analyzed its expression using exon-array data from a study of the spatio-temporal transcriptome of the human brain [19]. There is a steady increase in *EFR3A* mRNA levels in multiple brain regions through fetal development and into adolescence (Figure 2A). *In situ* hybridization of adult human dorsolateral prefrontal cortex revealed the presence of *EFR3A* in cortical neurons including pyramidal neurons (Figure 2B). This pattern is consistent with prior data on the expression of ASD genes [9,19], as well as functional annotation of genes that are highly co-expressed with ASD genes, showing enrichment for a category associated with the development of cortical projection (pyramidal) neurons [19].

We next identified the top 100 genes co-expressed with *EFR3A* (Additional file 16: Table S10) using the same dataset [19]. Gene ontology enrichment analysis using the Database for Annotation, Visualization and Integrated Discovery (DAVID v6.7) [28,29] revealed synaptic genes, including *SYNJ1*, the major PtdIns(4,5)P_2_ phosphatase in the brain [30], as the most significant finding (Figure 2C). We also compared the expression profile of *EFR3A* with a discrete module of co-expressed genes (M12) significantly associated with ASD in a prior transcriptome analysis of post-mortem autism and control brains [20]. M12 is enriched for genes involved in synaptic function, vesicular transport and neuronal projection and is downregulated in the autistic brain. We compared the distribution of expression correlation coefficients between *EFR3A* and M12 genes (Figure 2D) and between *EFR3A* and all brain-expressed genes [19] (Figure 2E). We found that the distribution between *EFR3A* and M12 genes was significantly skewed toward positive correlation coefficients compared to the distribution between *EFR3A* and all brain-expressed genes (*P* < 2.2 × 10^−16^, Wilcoxon test). When a similar analysis was performed on the homologue *EFR3B* (which is largely brain-expressed) and eight genes strongly associated with ASD from recent CNV and exome studies [3-6,25], *EFR3A* was the most strongly correlated with M12 expression (Figure 2D). We repeated this process with three additional modules of co-expressed genes (M2, M3 and M16) identified by a prior analysis of BrainSpan transcriptome data from normal brains [21]. All are significantly associated with ASD candidate genes, although M2 and M3 are enriched for early fetal transcriptional regulators affected by *de novo* loss-of-function mutations in ASD, while M16, which has significant overlap with M12, is enriched for synaptic genes upregulated during late fetal/early postnatal stages and genes harboring inherited common variants in ASD. As might be expected given its developmental expression pattern and synaptic function, *EFR3A* is positively correlated with M16 (Additional file 17: Figure S7A; *P* < 2.2 × 10^−16^, Wilcoxon test) and negatively correlated with M2 and M3 (Additional file 17: Figure S7B,C; *P* < 2.2 × 10^−16^, Wilcoxon test).

## Discussion

Our conservation, structure-based functional and expression analyses suggest a role for rare deleterious *EFR3A* mutations in the risk for ASD, adding to the emerging data on specific synaptic functions, including phosphoinositide metabolism, relevant to these disorders. Multiple resequencing projects for ASD have revealed numerous rare variants in both cases and controls. Given the over-representation of *de novo* loss-of-function mutations in cases, it is implausible that a subset of damaging missense mutations does not carry risk as well. However, differentiating relevant functional mutations from the large collection of neutral background variation remains a challenge. We have approached this issue by following up an observation of a *de novo* mutation in an ASD proband with a relatively large case/control analysis, relying on diverse approaches to identify putatively deleterious variants. While the overall burden of singleton variants was not impressive, the use of multiple conservation measures and crystal structure analysis to segregate functional variation showed consistent evidence for experiment-wide association with ASD.

Our results would clearly not survive correction for genome-wide comparisons. Of course, given the distribution of singleton mutations across the genome, this statistical threshold, if applied to every targeted analysis, would demand implausibly large case/control samples. In an effort to skirt this problem, we used an initial observation in an unbiased exome-wide study to establish a narrow hypothesis and then relied on an experiment-wide *P* value threshold for our case/control analysis. At present, this seems a reasonable approach to evaluating single gene association. Additional data on the distribution of *de novo* missense mutations in the genome and the integration of ASD risk associated with varying classes of mutations [31] with co-expression network data [11,21] will shed significant light on the contribution of any one gene to ASD.

Our expression data, combined with evidence for the involvement of *EFR3A* in synaptic phosphoinositide metabolism [8], suggest that *EFR3A* may play an important role in synaptic function during human fetal brain development. In addition to the significant conservation and structure-based findings, our analysis comparing the expression profile of *EFR3A* with M12 and M16 further suggests that this gene is associated with ASD. Not only are *EFR3A* and M12/M16 expression strongly correlated but *EFR3A* is also the most strongly correlated in the context of ASD-associated genes and its homologue *EFR3B*. Although co-expression data do not prove that a gene causes a disorder, they can provide another piece of supportive evidence [11,21]. The determination of when in development and in what cell types *EFR3A* is expressed provides insight into how *EFR3A* mutations might contribute to the pathophysiology of ASD.

A potential limitation of this study is that we combined data from Sanger sequencing (SSC cases and NINDS controls) and whole-exome sequencing (AASC cases/controls and NE controls). It is possible that the two techniques can yield different sets of variants. As described under Methods, for the NE controls, we determined that >98% of the coding and splice site sequences were covered by at least eight independent reads. To minimize false negatives in controls that might bias toward an excess of rare mutations in cases, a minimum of only one independent read was used to identify variants for confirmation. Regarding the AASC samples, case and control exome data were subjected to identical variant-calling approaches and filtering criteria within each site and were, therefore, treated equally, suggesting that any error should be randomly distributed between these groups.

Another limitation is that we did not find additional *de novo* mutations in the subjects for whom family DNA was available (only SSC cases), which would strengthen the association of *EFR3A* mutations with ASD. However, there is abundant evidence that inherited mutations also contribute to ASD [31]. The presence of SSC case mutations in unaffected parents and/or siblings points to incomplete penetrance, as expected in complex genetic disorders such as ASD. The crystal structure analysis was able to stratify the mutations further by determining that potentially deleterious variants were generally not shared by siblings. Although this is an interesting observation, it is based on a very small number of events (five SSC case mutations with both sibling data and crystal structure information) and cannot be assigned statistical significance. We did observe one premature stop codon mutation in an NE control as well as in an SSC case, indicating that *EFR3A* mutations are not sufficient to cause ASD. However, neuropsychiatric data was not available for this control cohort. Moreover, the identification of well-established ASD-associated variants in unscreened controls is so commonplace as to be expected in a study such as this one.

**Figure 3:**
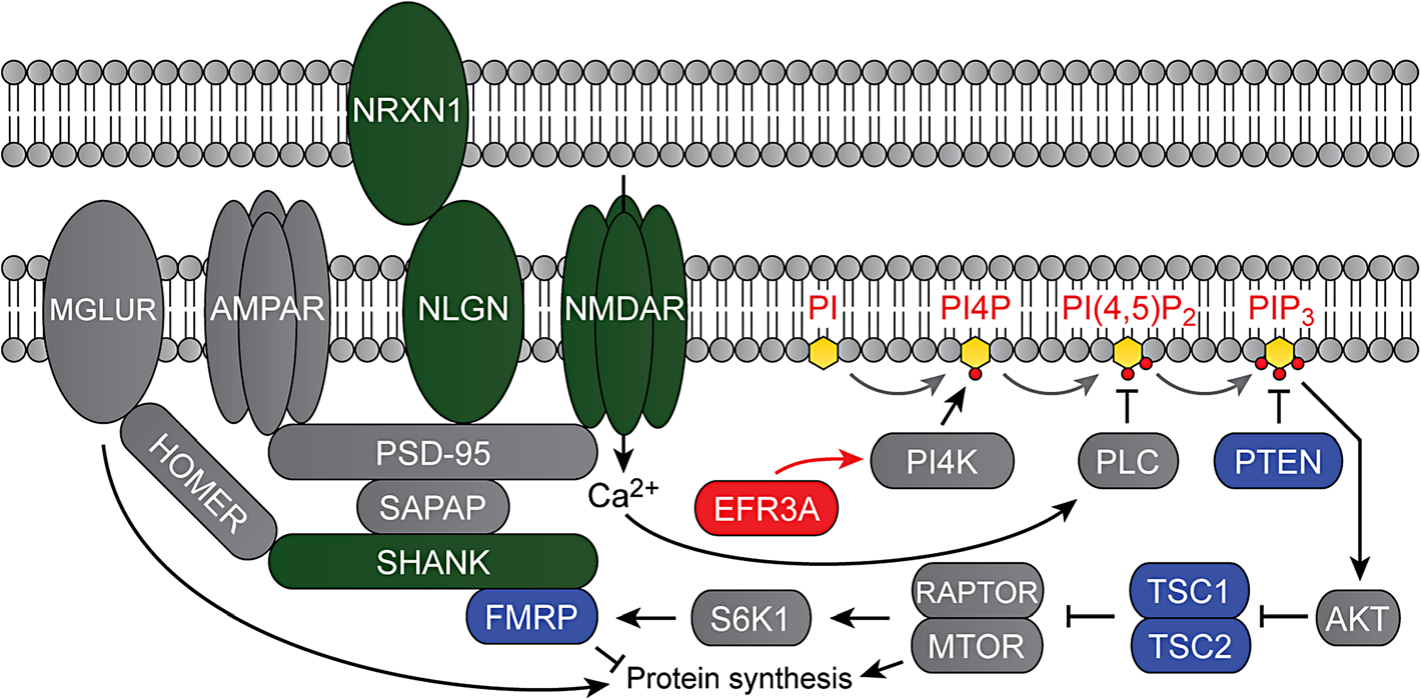
**ASD-related molecules at the synapse.** Mutations in proteins in green have been demonstrated to carry risk for idiopathic ASD, and mutations in proteins in blue cause syndromic forms of ASD. EFR3A has been linked to phosphoinositide metabolism [8].

EFR3A is a critical component of a complex containing a phosphatidylinositol 4-kinase that synthesizes the plasma membrane pool of the phosphoinositide PtdIns4P, the direct precursor of PtdIns(4,5)P_2_[8]. PtdIns(4,5)P_2_ has a wide variety of direct functions in the central nervous system, including regulation of exo/endocytosis, ion channel function, neurotransmitter receptors, and transporters and nucleation of the actin cytoskeleton [32,33]. Additionally, PtdIns(4,5)P_2_ is a precursor to numerous signaling metabolites: diacylglycerol and InsP_3_ (via phospholipase C activity), which are key regulators of Ca^2+^ signaling, and PtdIns(3,4,5)P_3_ (via PI 3-kinase activity), which mediates many cellular processes such as activation of the Akt/mTOR signaling pathway [30]. Mutations in *PTEN*, which encodes a PtdIns(3,4,5)P_3_ phosphatase, and in *TSC1* and *TSC2*, which are key effectors in the PtdIns(3,4,5)P_3_ signaling pathway, have demonstrated the importance of synaptic phosphoinositide signaling in syndromic forms of autism (Figure 3) [9,34,35]. Common polymorphisms and rare CNVs in *MET*, another gene involved in phosphoinositide metabolism, have implicated this pathway in idiopathic ASD as well [36,37].

The identification of rare deleterious mutations in *EFR3A*, a gene linked to PtdIns4P synthesis (Figure 3), further strengthens the role of phosphoinositide metabolism in ASD. The precise effects of EFR3A on the levels of various phosphoinositides still have to be determined, and an *EFR3A* knock-out mouse is not yet available. Delineating the molecular details and functional significance of interactions between EFR3A and its binding partners will allow the development of *in vitro* assays to assess further the severity of the variants we report here. Importantly, phosphoinositide metabolizing enzymes are pharmacologically targetable [38-40]. The applicability of this approach towards ASD has been shown for the closely connected mTOR pathway in mouse models of tuberous sclerosis [41,42]. Therefore, mutations in *EFR3A* and perturbations in phosphoinositide metabolism may point to a potential avenue for treatment in a subset of ASD patients.

## Conclusions

Rare nonsynonymous mutations in *EFR3A* are significantly more common among ASD cases than controls at positions that are conserved and positions that would be disruptive to protein structure and function based on analysis of the Efr3 crystal structure. These results further implicate phosphoinositide metabolism in the pathophysiology of ASD, a pathway that is pharmacologically targetable. Exactly how *EFR3A* mutations contribute to that pathophysiology will have to await further delineation of how the protein functions and the development of specific assays to test their severity.

## Availability of supporting data

NINDS Neurologically Normal Caucasian Control Panel: [http://ccr.coriell.org/Sections/Collections/NINDS/DNAPanels.aspx?PgId=195&coll=ND/]

AASC controls: [https://www.nimhgenetics.org/available_data/controls/]

NCBI dbSNP: [http://www.ncbi.nlm.nih.gov/snp]

EVS: [http://evs.gs.washington.edu/EVS/]

UCSC Genome Browser: [http://www.genome.ucsc.edu/]

SeattleSeq: [http://snp.gs.washington.edu/SeattleSeqAnnotation138/]

ConSurf: [http://consurf.tau.ac.il/]

DAVID v6.7: [http://david.abcc.ncifcrf.gov/]

## Abbreviations

AASC: ARRA Autism Sequencing Collaboration; ASD: autism spectrum disorder; BCA: bicinchoninic acid; CASK: *Calcium/Calmodulin-dependent Serine/Threonine Kinase*; CNV: copy number variant; DAVID: Database for Annotation, Visualization and Integrated Discovery; EFR3A: *Eighty-five Requiring 3A*; GERP: genomic evolutionary rate profiling; HEAT: Huntington, Elongation factor 3, regulatory subunit A of protein phosphatase 2A, and Target of rapamycin; IQR: interquartile range; NE: northern European; NINDS: National Institute of Neurological Disorders and Stroke; PCA: principal component analysis; PCR: polymerase chain reaction; PhyloP: phylogenetic *P* values; RBO: rolling blackout; RT: room temperature; siRNA: small interfering RNA; SNP: single nucleotide polymorphism; SSC: Simons Simplex Collection.

## Competing interests

The authors declare that they have no competing interests.

## Authors’ contributions

ARG conceived and designed the study, collected and analyzed data, and wrote and gave final approval for the manuscript. HJK, MC, LL, AGE-S and BNM collected and analyzed data, and edited and gave final approval for the manuscript. MP, FC, JMB and DF collected and analyzed data, made a critical revision and gave final approval for the manuscript. TVF, JDM, LK, MJD, RPL and HZ analyzed the data, and edited and gave final approval for the manuscript. PDC and NS analyzed data, made a critical revision and gave final approval for the manuscript. MWS conceived and designed the study, provided financial support, analyzed data, and wrote and gave final approval for the manuscript. All authors read and approved the final manuscript.

## Supplementary Material

Additional file 1: Figure S1 Scree plot of the first 50 components from principal component analysis identifies three principal components that contribute the greatest amount of variation. Click here for file

Additional file 2: Figure S2 Three largest principal components of genotypes for all SSC cases, NINDS controls and NE controls were plotted against one another. EV, eigenvalue; PC, principal component. Click here for file

Additional file 3: Figure S3 Interquartile range (IQR) distance around the median of the study population cluster was calculated. A threshold that included all of the NINDS and NE controls was determined to lie at 5 IQRs from the third quartile, and 54 SSC cases beyond this threshold were excluded as ancestral outliers. Included samples are in blue; excluded samples (outliers) are in green. EV, eigenvalue; PC, principal component. Click here for file

Additional file 4: Table S1 Primers used for PCR and Sanger sequencing. Click here for file

Additional file 5: Figure S4 Multispecies protein sequence alignment of EFR3A/B for ConSurf analysis. Click here for file

Additional file 6: Figure S5 Conservation structure of the EFR3A protein as determined by ConSurf. Click here for file

Additional file 7: Table S2 List of the genes in M12, M16, M2 and M3 and the subset of each which is included in the exon-array platform used by Kang *et al.* (2011). These subsets were used for the comparisons made in Figure 2D,E, and Additional file 17: Figure S7. Click here for file

Additional file 8: Table S3 Median expression correlation coefficients for *ACTB*, *CHD8*, *DYRK1A*, *EFR3A*, *EFR3B*, *GRIN2B*, *KATNAL2*, *NRXN1*, *SCN2A*, *SHANK2* and *SHANK3* compared to M12, M16, M2, M3 and all brain-expressed genes. Click here for file

Additional file 9: Table S4 Binary entries used for permutation test. Click here for file

Additional file 10: Table S5 All rare nonsynonymous *CASK* variants in SSC cases. Click here for file

Additional file 11: Table S6 All rare nonsynonymous *EFR3A *variants. Click here for file

Additional file 12: Table S7 Severity of novel nonsynonymous singleton *EFR3A* mutations informed by Efr3 crystal structure. Click here for file

Additional file 13: Table S8 Molecular modeling of EFR3A protein. Molecular modeling was accomplished by inputting reference sequences into the I-TASSER [1,2], Phyre2 [3], Raptor [4] and HHpred [5] web servers. The Protein Data Bank identification codes for template structures are indicated, with the best matches for each run in bold and the error assessment for each server shown. An independent technique for detecting and scoring HEAT repeats was also used [6]. Using this technique, three HEAT repeats were detected with an *E* value less than 50, the benchmark for significance. Additionally, six HEAT repeats were detected by the REP server [7]. Click here for file

Additional file 14: Table S9 Top 100 genes co-expressed with *EFR3A*. Click here for file

Additional file 15: Figure S6 Expression analysis of mouse *EFR3A*. **(A)***EFR3A* is expressed in several mouse tissues, including the brain, as analyzed by Western blot. **(B)** EFR3A antibody specificity is verified by Western blot analysis of lysates from HeLa cells treated with control siRNA (−) or three different siRNA duplexes against human *EFR3A*. Although this antibody works well for Western blots, it does not work well for immunofluorescence, so we were not able to provide data for protein subcellular localization. Click here for file

Additional file 16: Table S10 siRNA sequences used to verify EFR3A antibody specificity. Click here for file

Additional file 17: Figure S7 Co-expression analysis of human *EFR3A*. Distribution of expression correlation coefficients of *EFR3A* and ASD genes with **(A)** M16, **(B)** M2 and **(C)** M3 genes. The homologue *EFR3B* is shown for comparison and *ACTB*, a housekeeping gene, is included as a negative control. Click here for file
