# Supplementary figures and images for: Rare deleterious mutations of the gene EFR3A in autism spectrum disorders
Source: Mol Autism. 2014 Apr 29;5:31. doi: 10.1186/2040-2392-5-31 (PMC4032628; doi:10.1186/2040-2392-5-31)

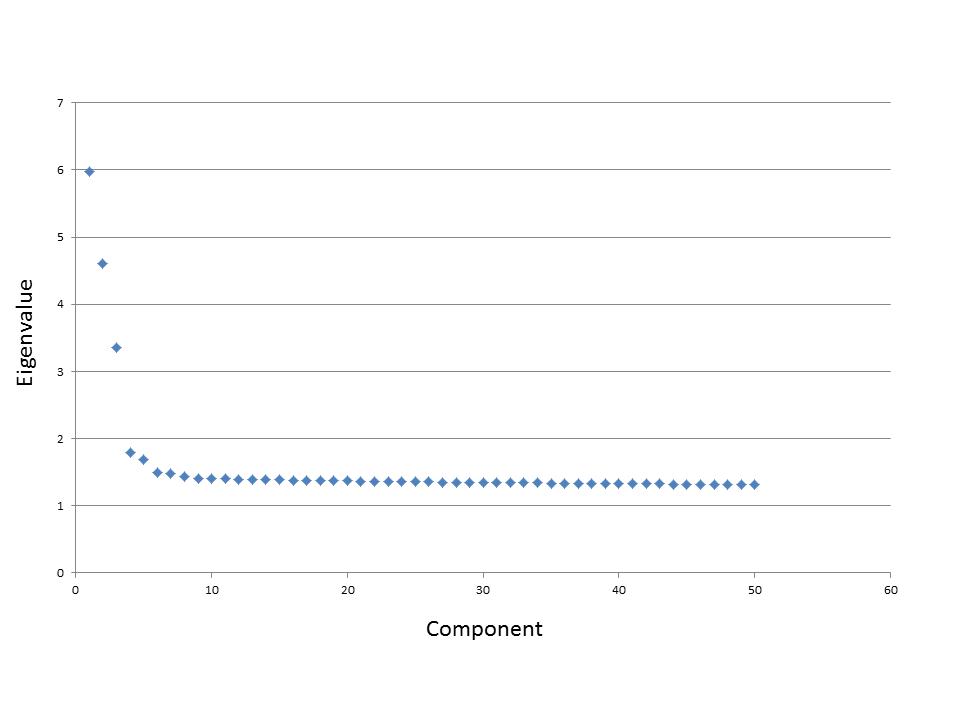

Supplement: Additional file 1: Figure S1 — Scree plot of the first 50 components from principal component analysis identifies three principal components that contribute the greatest amount of variation. [file 2040-2392-5-31-S1.jpeg]

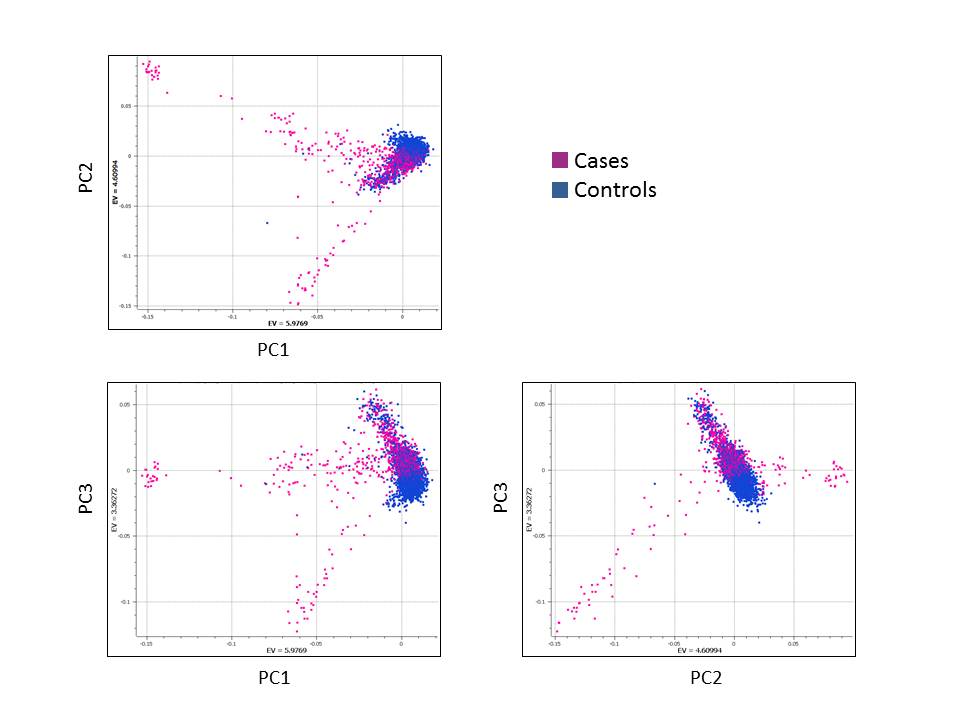

Supplement: Additional file 2: Figure S2 — Three largest principal components of genotypes for all SSC cases, NINDS controls and NE controls were plotted against one another. EV, eigenvalue; PC, principal component. [file 2040-2392-5-31-S2.jpeg]

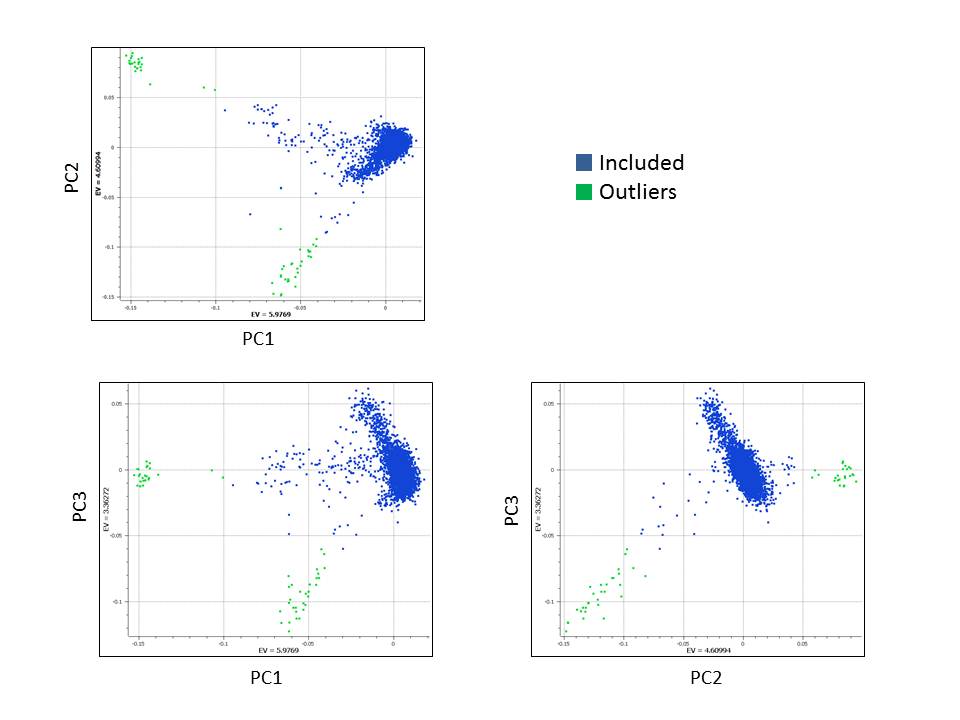

Supplement: Additional file 3: Figure S3 — Interquartile range (IQR) distance around the median of the study population cluster was calculated. A threshold that included all of the NINDS and NE controls was determined to lie at 5 IQRs from the third quartile, and 54 SSC cases beyond this threshold were excluded as ancestral outliers. Included samples are in blue; excluded samples (outliers) are in green. EV, eigenvalue; PC, principal component. [file 2040-2392-5-31-S3.jpeg]

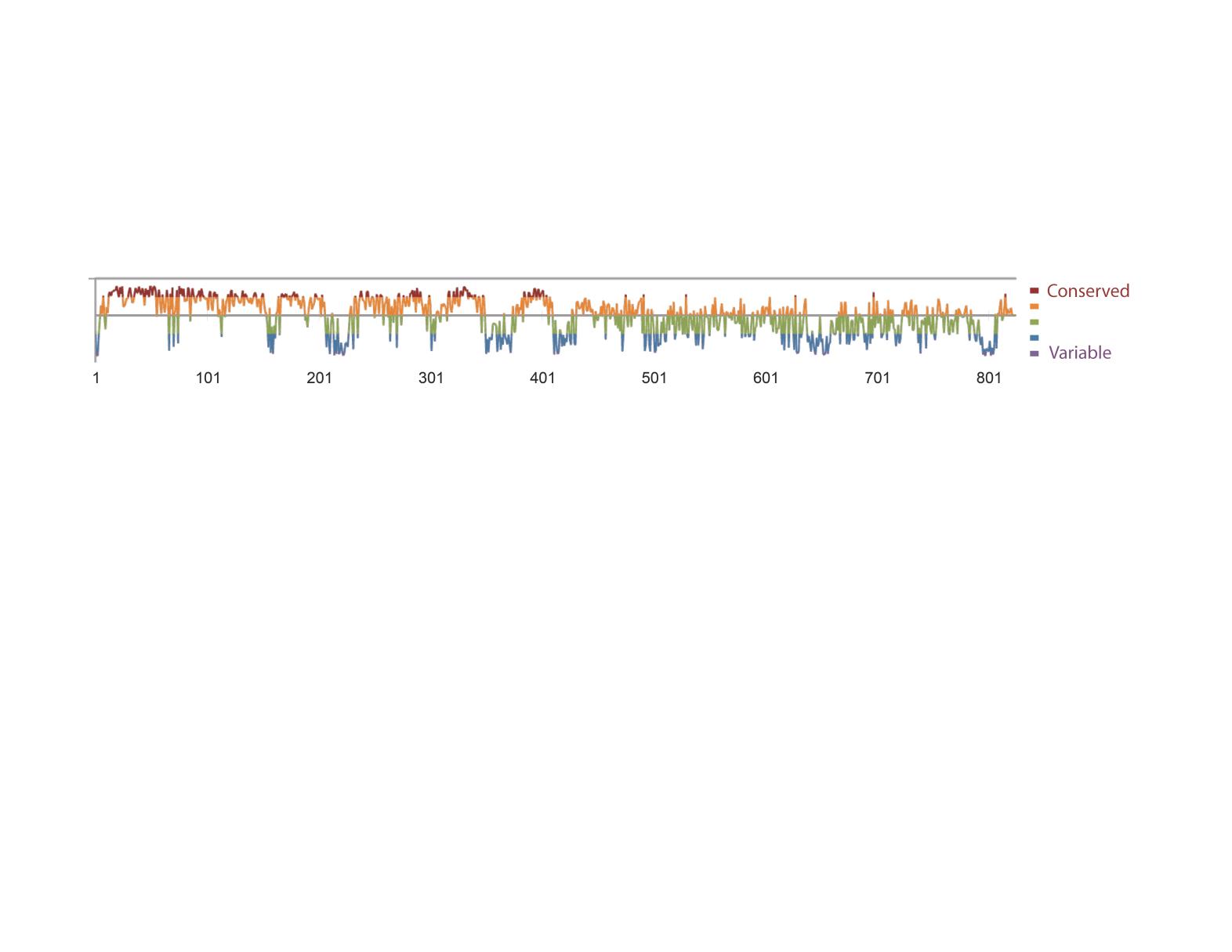

Supplement: Additional file 6: Figure S5 — Conservation structure of the EFR3A protein as determined by ConSurf. [file 2040-2392-5-31-S6.jpeg]

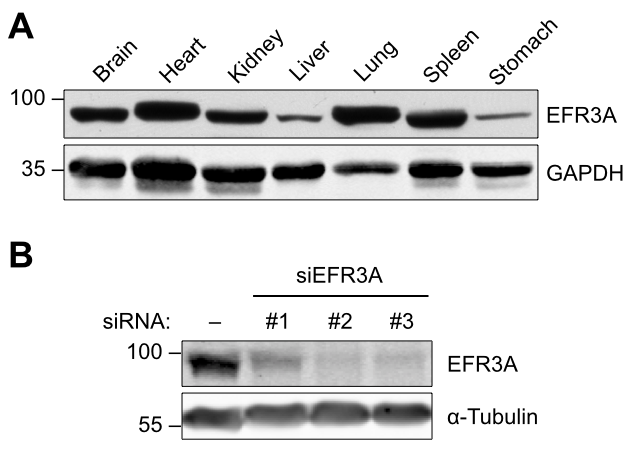

Supplement: Additional file 15: Figure S6 — Expression analysis of mouse EFR3A. (A)EFR3A is expressed in several mouse tissues, including the brain, as analyzed by Western blot. (B) EFR3A antibody specificity is verified by Western blot analysis of lysates from HeLa cells treated with control siRNA (−) or three different siRNA duplexes against human EFR3A. Although this antibody works well for Western blots, it does not work well for immunofluorescence, so we were not able to provide data for protein subcellular localization. [file 2040-2392-5-31-S15.tiff]

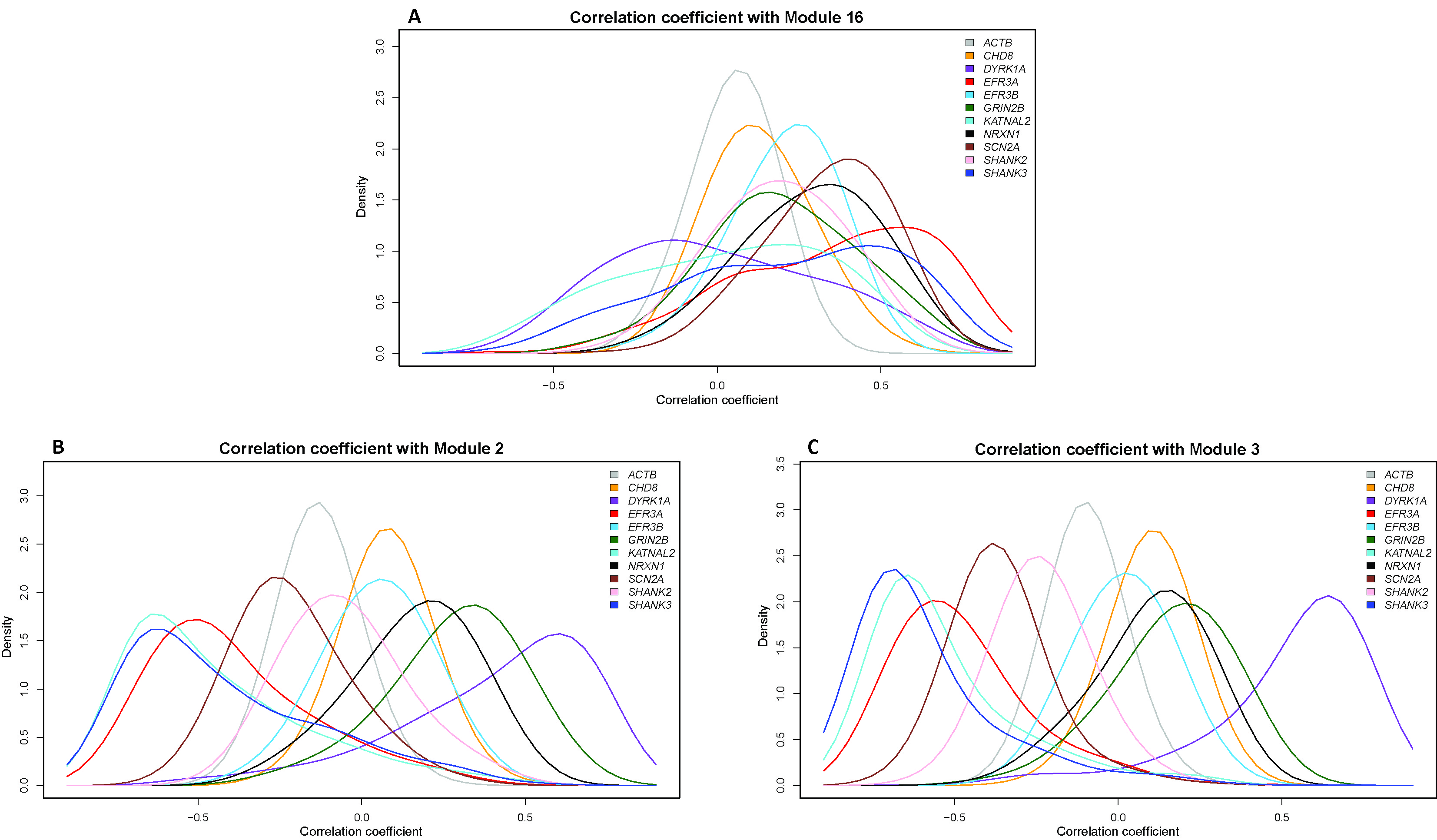

Supplement: Additional file 17: Figure S7 — Co-expression analysis of human EFR3A. Distribution of expression correlation coefficients of EFR3A and ASD genes with (A) M16, (B) M2 and (C) M3 genes. The homologue EFR3B is shown for comparison and ACTB, a housekeeping gene, is included as a negative control. [file 2040-2392-5-31-S17.jpeg]
