# Supplementary material for: Rare deleterious mutations of the gene EFR3A in autism spectrum disorders
Source: Mol Autism. 2014 Apr 29;5:31. doi: 10.1186/2040-2392-5-31 (PMC4032628; doi:10.1186/2040-2392-5-31)
Supplement: Additional file 5: Figure S4 — Multispecies protein sequence alignment of EFR3A/B for ConSurf analysis. [file 2040-2392-5-31-S5.pdf]

```
Cryptococcus_neoform_0        ------------------------------------------------------------
Drosophila_melanogas_1        MA----------------------------------------------------------
EFR3A_Equus_caballus_2        ------------------------------------------------------------
Neurospora_crassa_3           ------------------------------------------------------------
Debaryomyces_hanseni_4        ------------------------------------------------------------
Saccharomyces_cerevi_5        ------------------------------------------------------------
Ustilago_maydis_6             ------------------------------------------------------------
Ascaris_suum_7                ------------------------------------------------------------
Harpegnathos_saltato_8        ------------------------------------------------------------
Monodelphis_domestic_9        ------------------------------------------------------------
Ailuropoda_melanoleu_10       ------------------------------------------------------------
Ashbya_gossypii_11            ------------------------------------------------------------
Nematostella_vectens_12       ------------------------------------------------------------
Acyrthosiphon_pisum_13        ------------------------------------------------------------
Culex_quinquefasciat_14       ------------------------------------------------------------
Aedes_aegypti_15              ------------------------------------------------------------
Camponotus_floridanu_16       ------------------------------------------------------------
Caenorhabditis_elega_17       ------------------------------------------------------------
EFR3B_Homo_sapiens_18         ------------------------------------------------------------
EFR3A_Homo_sapiens_19         ------------------------------------------------------------
EFR3B_Ailuropoda_mel_20       MEEHVRQYKNTAGERKMTPSAGTVINGGLSRKIQQMPGSTQHDDFKTSDHPLPWLGGGSY
Schizosaccharomyces__21       ------------------------------------------------------------
EFR3A_Xenopus_laevis_22       ------------------------------------------------------------
EFR3A_Taeniopygia_gu_23       ------------------------------------------------------------
EFR3B_Danio_rerio_24          ------------------------------------------------------------
Anopheles_darlingi_25         ------------------------------------------------------------
EFR3B_Mus_musculus_26         ------------------------------------------------------------
Yarrowia_lipolytica_27        ------------------------------------------------------------
EFR3A_Rattus_norvegi_28       ------------------------------------------------------------
Gallus_gallus_29              ------------------------------------------------------------
Kluyveromyces_lactis_30       ------------------------------------------------------------
Ornithorhynchus_anat_31       MDGEEGHAISQVTFWTRAGGARENAMPTLPRETLSQSDLTHRTSGVVSFGPVFLVGTVLK
EFR3A_Oryctolagus_cu_32       ------------------------------------------------------------
Nasonia_vitripennis_33        ------------------------------------------------------------
Candida_glabrata_34           ------------------------------------------------------------
Ixodes_scapularis_35          ------------------------------------------------------------
Daphnia_pulex_36              ------------------------------------------------------------
Tribolium_castaneum_37        MS----------------------------------------------------------
Anopheles_gambiae_38          ------------------------------------------------------------
EFR3B_Ciona_intestin_39       ------------------------------------------------------------
Tetraodon_nigrovirid_40       ------------------------------------------------------------
Solenopsis_invicta_41         ------------------------------------------------------------

Cryptococcus_neoform_0        ------------------------------------------------------------
Drosophila_melanogas_1        --------------------------------LIRCCFEPPELPEFFDSFVQKCTDPSC-
EFR3A_Equus_caballus_2        -------------------------------------------------------MPTR-
Neurospora_crassa_3           ------------------------------------------------------------
Debaryomyces_hanseni_4        ------------------------------------------------------------
Saccharomyces_cerevi_5        ------------------------------------------------------------
Ustilago_maydis_6             ------------------------------------------------------------
Ascaris_suum_7                --------------------------------------------------------MDG-
Harpegnathos_saltato_8        ------------------------------------------------------------
Monodelphis_domestic_9        ------------------------------------------------MSRGHGNMTRG-
Ailuropoda_melanoleu_10       -------------MKDYIQVPAAELDKPYELPSTRESTAFLWLSRSNDFILLVLVMFTR-
Ashbya_gossypii_11            ------------------------------------------------------------
Nematostella_vectens_12       ------------------------------------------------------MATSH-
Acyrthosiphon_pisum_13        ----------------------------------------------------------M-
Culex_quinquefasciat_14       -------------------------------MSIIKCCFEPVEMPEFLDSFVKKCTEGC-
Aedes_aegypti_15              -------------------------------MSMIRCCFEPVEMPEFLDSFVKKCTEGC-
Camponotus_floridanu_16       --------------------------------------------------------MFG-
Caenorhabditis_elega_17       --------------------------------------------------------MNG-
EFR3B_Homo_sapiens_18         --------------------------------------------------------MYG-
EFR3A_Homo_sapiens_19         -------------------------------------------------------MPTR-
EFR3B_Ailuropoda_mel_20       QGDRSVQETASGKRAKEKETGLQSVGVAGTPMLLADKGSEEIGKEEQTRKKHQDVMPWGL
Schizosaccharomyces__21       ------------------------------------------------------------
```

```
EFR3A_Xenopus_laevis_22        -------------------------------------------------------MPAG-
EFR3A_Taeniopygia_gu_23        ----------------------------MRQYVQVPTAEQEKAYDYNSAFEPRSCMCLG-
EFR3B_Danio_rerio_24           --------------------------------------------------------MTG-
Anopheles_darlingi_25          -------------------------------MSMIKCCFEPLEMPEFLDSFVKKCTEGC-
EFR3B_Mus_musculus_26          --------------------------------------------------------MYG-
Yarrowia_lipolytica_27         ------------------------------------------------------------
EFR3A_Rattus_norvegi_28        -------------------------------------------------------MPTR-
Gallus_gallus_29               ------------------------------------------------------MGRKG-
Kluyveromyces_lactis_30        ------------------------------------------------------------
Ornithorhynchus_anat_31        AAFNIITGGVAVANGRGPRPGRLVQLGKAGWACQGPPSPRPLHRPPAAPGFGLGQAPSRL
EFR3A_Oryctolagus_cu_32        -------------MKDYVQVPAAELEKPYEFTSRESTTFLYPMIMKNITSVLLVTDRCG-
Nasonia_vitripennis_33         -------------------------------MALIRCCFEAEAPDIFESVVGKCTDPGC-
Candida_glabrata_34            -------------------------------------------------------MQLG-
Ixodes_scapularis_35           --------------------------------------------------------IAG-
Daphnia_pulex_36               -------------------------------MSVWSTLSDSSFLEMLDTVLSKCDASYC-
Tribolium_castaneum_37         --------------------------------FIKCCFDGIEIAEVLDTFAQKCTDPGC-
Anopheles_gambiae_38           --------------------------------------------------------MLG-
EFR3B_Ciona_intestin_39        ------------------------------------------------------------
Tetraodon_nigrovirid_40        ------------------------------------------------------------
Solenopsis_invicta_41          ----------------------------------------------------------G-

Cryptococcus_neoform_0         ------------------------------------------------------------
Drosophila_melanogas_1         ------------------------------------------------------------
EFR3A_Equus_caballus_2         ------------------------------------------------------------
Neurospora_crassa_3            ------------------------------------------------------------
Debaryomyces_hanseni_4         ------------------------------------------------------------
Saccharomyces_cerevi_5         ------------------------------------------------------------
Ustilago_maydis_6              ------------------------------------------------------------
Ascaris_suum_7                 ------------------------------------------------------------
Harpegnathos_saltato_8         ------------------------------------------------------------
Monodelphis_domestic_9         ------------------------------------------------------------
Ailuropoda_melanoleu_10        ------------------------------------------------------------
Ashbya_gossypii_11             ------------------------------------------------------------
Nematostella_vectens_12        ------------------------------------------------------------
Acyrthosiphon_pisum_13         ------------------------------------------------------------
Culex_quinquefasciat_14        ------------------------------------------------------------
Aedes_aegypti_15               ------------------------------------------------------------
Camponotus_floridanu_16        ------------------------------------------------------------
Caenorhabditis_elega_17        ------------------------------------------------------------
EFR3B_Homo_sapiens_18          ------------------------------------------------------------
EFR3A_Homo_sapiens_19          ------------------------------------------------------------
EFR3B_Ailuropoda_mel_20        ECDWFSPGMGSSRFCAGRGMFQEEACELEKEALEQKG-----------------------
Schizosaccharomyces__21        ------------------------------------------------------------
EFR3A_Xenopus_laevis_22        ------------------------------------------------------------
EFR3A_Taeniopygia_gu_23        ------------------------------------------------------------
EFR3B_Danio_rerio_24           ------------------------------------------------------------
Anopheles_darlingi_25          ------------------------------------------------------------
EFR3B_Mus_musculus_26          ------------------------------------------------------------
Yarrowia_lipolytica_27         ------------------------------------------------------------
EFR3A_Rattus_norvegi_28        ------------------------------------------------------------
Gallus_gallus_29               ------------------------------------------------------------
Kluyveromyces_lactis_30        ------------------------------------------------------------
Ornithorhynchus_anat_31        TGSLRRSSAGPWLTPGRALPCAQYFGRLAEAKQLPLNQVPQNSSDECLLCTEHWGNGWML
EFR3A_Oryctolagus_cu_32        ------------------------------------------------------------
Nasonia_vitripennis_33         ------------------------------------------------------------
Candida_glabrata_34            ------------------------------------------------------------
Ixodes_scapularis_35           ------------------------------------------------------------
Daphnia_pulex_36               ------------------------------------------------------------
Tribolium_castaneum_37         ------------------------------------------------------------
Anopheles_gambiae_38           ------------------------------------------------------------
EFR3B_Ciona_intestin_39        ------------------------------------------------------------
Tetraodon_nigrovirid_40        ------------------------------------------------------------
Solenopsis_invicta_41          ------------------------------------------------------------

Cryptococcus_neoform_0         ------------------------------------------------------------
```

```
Drosophila_melanogas_1        ------------------------------------------------------------
EFR3A_Equus_caballus_2        ------------------------------------------------------------
Neurospora_crassa_3           ------------------------------------------------------------
Debaryomyces_hanseni_4        ------------------------------------------------------------
Saccharomyces_cerevi_5        ------------------------------------------------------------
Ustilago_maydis_6             ------------------------------------------------------------
Ascaris_suum_7                ------------------------------------------------------------
Harpegnathos_saltato_8        ------------------------------------------------------------
Monodelphis_domestic_9        ------------------------------------------------------------
Ailuropoda_melanoleu_10       ------------------------------------------------------------
Ashbya_gossypii_11            ------------------------------------------------------------
Nematostella_vectens_12       ------------------------------------------------------------
Acyrthosiphon_pisum_13        ------------------------------------------------------------
Culex_quinquefasciat_14       ------------------------------------------------------------
Aedes_aegypti_15              ------------------------------------------------------------
Camponotus_floridanu_16       ------------------------------------------------------------
Caenorhabditis_elega_17       ------------------------------------------------------------
EFR3B_Homo_sapiens_18         ------------------------------------------------------------
EFR3A_Homo_sapiens_19         ------------------------------------------------------------
EFR3B_Ailuropoda_mel_20       ------------------------------------------------------------
Schizosaccharomyces__21       ------------------------------------------------------------
EFR3A_Xenopus_laevis_22       ------------------------------------------------------------
EFR3A_Taeniopygia_gu_23       ------------------------------------------------------------
EFR3B_Danio_rerio_24          ------------------------------------------------------------
Anopheles_darlingi_25         ------------------------------------------------------------
EFR3B_Mus_musculus_26         ------------------------------------------------------------
Yarrowia_lipolytica_27        ------------------------------------------------------------
EFR3A_Rattus_norvegi_28       ------------------------------------------------------------
Gallus_gallus_29              ------------------------------------------------------------
Kluyveromyces_lactis_30       ------------------------------------------------------------
Ornithorhynchus_anat_31       VAVRIASRTVDVIPVKVIWGDPNEELLSSTNAAYLLLLGRECSHVRSRKQLDKLPEHEVT
EFR3A_Oryctolagus_cu_32       ------------------------------------------------------------
Nasonia_vitripennis_33        ------------------------------------------------------------
Candida_glabrata_34           ------------------------------------------------------------
Ixodes_scapularis_35          ------------------------------------------------------------
Daphnia_pulex_36              ------------------------------------------------------------
Tribolium_castaneum_37        ------------------------------------------------------------
Anopheles_gambiae_38          ------------------------------------------------------------
EFR3B_Ciona_intestin_39       ------------------------------------------------------------
Tetraodon_nigrovirid_40       ------------------------------------------------------------
Solenopsis_invicta_41         ------------------------------------------------------------

Cryptococcus_neoform_0        ----------------------MGCIPCR-TLQP---EVAHLNACYPPPKALLTAGPEYR
Drosophila_melanogas_1        --------------------CCGCCSALRPRYKR---LV---DNIFP-------VNPEDG
EFR3A_Equus_caballus_2        --------------------VCCCCSALRPRYKR---LV---DNIFP-------EDPKDG
Neurospora_crassa_3           -----------------------MNALQQKCRPKHQVLV---LKCYPRTIK---GAVDVK
Debaryomyces_hanseni_4        ------------------------MPIFRPKHQK---LI---LQCYPPG-----KGVDKK
Saccharomyces_cerevi_5        -------------------MQLSMRMMFTPKHQK---LV---NQCYPTG-----RTTDKK
Ustilago_maydis_6             ----------------------MC--IPKSNHKK---LV---DDCYPPPKALITSAPEYR
Ascaris_suum_7                --------------------LMFCCAPCKPRYRR---LV---DAIYP-------RSLTEG
Harpegnathos_saltato_8        --------------------CCWCCSALRPRYKR---LV---DNIFP-------VNPQDG
Monodelphis_domestic_9        ------------------GRICCCCGALRPRFKR---LV---DNIFP-------EDPKDG
Ailuropoda_melanoleu_10       --------------------VCCCCSALRPRYKR---LV---DNIFP-------EDPKDG
Ashbya_gossypii_11            -----------------------MGLLFTPKHQK---LV---NQCYPTG-----RTPDKK
Nematostella_vectens_12       --------------------ICSCFGNFGPRYKR---LV---DNIFP-------ADARSG
Acyrthosiphon_pisum_13        --------------------CVGCLSRMRPRYKR---LV---DNIFP-------SVPQDG
Culex_quinquefasciat_14       --------------------CCGCCSALRPRYKR---LV---DNIFP-------VNPEDG
Aedes_aegypti_15              --------------------CCGCCSALRPRYKR---LV---DNIFP-------VNPEDG
Camponotus_floridanu_16       --------------------CCWCCSALRPRYKR---LV---DNIFP-------VNPQDG
Caenorhabditis_elega_17       --------------------LC-CCTPCKPRYRR---LV---DSIYP-------RAVTDG
EFR3B_Homo_sapiens_18         --------------------VCGCCGALRPRYKR---LV---DNIFP-------EDPEDG
EFR3A_Homo_sapiens_19         --------------------VCCCCSALRPRYKR---LV---DNIFP-------EDPKDG
EFR3B_Ailuropoda_mel_20       ------------------GRVCGCCGALRPRYKR---LV---DNIFP-------EDPEDG
Schizosaccharomyces__21       -----------------------MWLLFRSKHKK---LV---LRCFPSG-----KLGETE
EFR3A_Xenopus_laevis_22       --------------------ICGCCGALRPRYKR---LV---DNIFP-------EDPRDG
```

```
EFR3A_Taeniopygia_gu_23       ---------------------VCCCCAALRPRYKR---LV---DNIFP-------EDPKDG
EFR3B_Danio_rerio_24          ---------------------VCGCCGALRPRYKR---LV---DNIFP-------EDPEDG
Anopheles_darlingi_25         ---------------------CCGCCSALRPRYKR---LV---DNIFP-------ANPEDG
EFR3B_Mus_musculus_26         ---------------------VCGCCGALRPRYKR---LV---DNIFP-------EDPEDG
Yarrowia_lipolytica_27        -----------------------MAPGIPKPRHQR---LV---LQCYPDG-----QAADKK
EFR3A_Rattus_norvegi_28       ---------------------VCCCCSALRPRYKR---LV---DNIFP-------EDPKDG
Gallus_gallus_29              ---------------------VCCCCAALRPRYKR---LV---DNIFP-------EDPKDG
Kluyveromyces_lactis_30       ------------------------MGFLFTPKHQK---LV---NQCYPPG-----RTPDKK
Ornithorhynchus_anat_31       REGLCHHGCRTAEKLIIPSRVCCCCAALRPRYKR---LV---DNIFP-------EDPKDG
EFR3A_Oryctolagus_cu_32       ---------------------VCCCCSALRPRYKR---LV---DNIFP-------EDPKDG
Nasonia_vitripennis_33        ---------------------CCWCCSALRPRYKR---LV---DNIFP-------VNPQDG
Candida_glabrata_34           --------------------------HIFTPKHQK---LV---NQCYPSG-----RAPDKK
Ixodes_scapularis_35          ---------------------CCGCCAALRPRYKR---LV---DNIFP-------ANPEDG
Daphnia_pulex_36              ---------------------CCGCCAALRPRYKR---LV---DNIFP-------VNPEDG
Tribolium_castaneum_37        ---------------------CCGCCSAFRPRYKR---FV---DNIFP-------VYPQDG
Anopheles_gambiae_38          ---------------------CCGCCSALRPRYKR---LV---DNIFP-------ANPEDG
EFR3B_Ciona_intestin_39       ------------------MSCCSCFSALRPQYKR---LV---DSIYP-------ANPEDG
Tetraodon_nigrovirid_40       ---------------------VCGCCGPLRPRYKR---LV---DNIFP-------EDPKDG
Solenopsis_invicta_41         ---------------------CCWCCAALRPRYKR---LV---DNIFP-------VNPQDG

Cryptococcus_neoform_0        PLAQDLSKLTYFATNKPSKLAKIGEELEKRVAQESARAS---SGNHKYRASLLISLAILR
Drosophila_melanogas_1        LVKSNMEKLTFYSLSSPDKLDRIGEYLYQKATKDINRKR---YKL------AEIAMEAMD
EFR3A_Equus_caballus_2        LVKADMEKLTFYAVSAPEKLDRIGSYLAERLSRDVVRHR---SGY------VLIAMEALD
Neurospora_crassa_3           PNSSELSYLLFYCQSRRAKIQKVGSFLEKKTASDVYHQR---IGN------VQVTLQILA
Debaryomyces_hanseni_4        PNPSELSYLLYYASTRRVKLEKVVTFLDRKTTSDAKHNR---AGN------LQVTLTIIS
Saccharomyces_cerevi_5        PKSSETSYLLYYVNSRRSKLEKVSTYLIKRSTSDLNHRR---IGN------IAVTLDLMN
Ustilago_maydis_6             PNSNELGRLTYYAQNKPAKLTKVGNLLESKAQADARAAKASGPAADKGKAALMITLAITK
Ascaris_suum_7                LIHTNMQKLTFYAISQPEKLDRIGEYIVWRMSRDLYRQR---YNQ------VKISVEAMD
Harpegnathos_saltato_8        LIKNNMEKLTFYSLSSPEKLDRIGEYLFQRASRDIYRRR---NGF------VVIAMEAMD
Monodelphis_domestic_9        LVKGDMEKLTFYAVSAPEKLDRIGTYLADKLTRDVVRHR---TGN------VLIAMEALD
Ailuropoda_melanoleu_10       LVKADMEKLTFYAVSAPEKLDRIGSYLAERLSRDVVRHR---SGY------VLIAMEALD
Ashbya_gossypii_11            PKSSETSYLLYYVNSRRTKLEKVSAYLVKRTAADLAHRR---IGN------VMVTLELAE
Nematostella_vectens_12       LVKANMDKLIFYALSSPEKLDRIGTYLARKLTRFVDRKR---YDF------VRISMEALD
Acyrthosiphon_pisum_13        LVKNNMEKLTFYALSSPEKLDRIGEYLFQKASRDISRKR---NEF------VMIAMEAMD
Culex_quinquefasciat_14       LVKANMEKLTFYSLRSPEKLDRIGEYLYQRASKDINRKR---YKF------VEIAMESMD
Aedes_aegypti_15              LVKSNMEKLTFYSLRSPEKLDRIGEYLYQRASKDINRKR---YKF------VEIAMEAMD
Camponotus_floridanu_16       LIKNNMEKLTFYSLSSPEKLDRIGEYLFQRASRDIYRRR---NGF------VVIAMEAMD
Caenorhabditis_elega_17       LLYSNMQKLTFYAISHPEKLERIGEYLVMRMVRDLSRQR---PVQ------VKIAVEAMD
EFR3B_Homo_sapiens_18         LVKTNMEKLTFYALSAPEKLDRIGAYLSERLIRDVGRHR---YGY------VCIAMEALD
EFR3A_Homo_sapiens_19         LVKTDMEKLTFYAVSAPEKLDRIGSYLAERLSRDVVRHR---SGY------VLIAMEALD
EFR3B_Ailuropoda_mel_20       LVKTNMEKLTFYALSAPEKLDRIGAYLSERLIRDVGRHR---YGY------VCIAMEALD
Schizosaccharomyces__21       PNGSPLAYLSYYAASNSSKLRKVAHFLGSRVRHSYYHKR---DNE------VIIGLKICK
EFR3A_Xenopus_laevis_22       LVKADMEKLTFYAVSAPEKLDRIGAYLAERLSRDVMRHR---YGN------VFIAMEALD
EFR3A_Taeniopygia_gu_23       LVKADMEKLTFYAVSAPEKLDRIGAYLAERLTRDVARHR---YGY------VLIAMEALD
EFR3B_Danio_rerio_24          LVKANMEKLTFYALSAPEKLDRIGAYLSERLSRDVARHR---YGY------VCIAMEALD
Anopheles_darlingi_25         LVKSNMEKLTFYSLRSPEKLDRIGEYLYQRASKDIYRKR---YKF------VEIAMEAMD
EFR3B_Mus_musculus_26         LVKTNMEKLTFYALSAPEKLDRIGAYLSERLIRDVGRHR---YGY------VCIAMEALD
Yarrowia_lipolytica_27        PNPSELSYLLFYVNHRRVKLEKVGPFLENKCYKDVSRGR---QGN------VMVALDIFA
EFR3A_Rattus_norvegi_28       LVKADMEKLTFYAVSAPEKLDRIGAYLAERLSRDVVRHR---SGY------VLIAMEALD
Gallus_gallus_29              LVKADMEKLTFYAVSAPEKLDRIGAYLAERLSRDVVRHR---YGY------VLIAMEALD
Kluyveromyces_lactis_30       PKGSETSYLLYYVNSRRPKLEKVSSYLVKRSTTDLNRRR---SGN------VSVTLELLA
Ornithorhynchus_anat_31       LVKADMEKLTFYAVSAPEKLDRIGSYLADRLSRDVVRHR---SGY------VLIAMEALD
EFR3A_Oryctolagus_cu_32       LVKADMEKLTFYAVSAPEKLDRIGAYLAERLSRDVVRHR---SGY------VLIAMEALD
Nasonia_vitripennis_33        LIKNNMEKLMFYSLSSPEKLDRIGEYLFQRASRDISRRR---NGF------VVIAMEAMD
Candida_glabrata_34           PKSSETSYLIYYVNSRSSKLEKVSNYLIKRTNTDLSRRR---VGN------VCVTLELMA
Ixodes_scapularis_35          LVRNNMEKLTFYALSSPEKLDRIGEYLAVRVSRDISRHR---VRY------VEISMEAMD
Daphnia_pulex_36              LVRSNMDKLTFYSMSSPEKLDRIGEYLAQRVSRDIYRHR---NPM------VIIAMEAMD
Tribolium_castaneum_37        LVKNNMEKLTFYALSSPEKLDRIGEYMYQRAARDIYRRR---YGF------VIIAMEAMD
Anopheles_gambiae_38          LVKSNMEKLTFYSLRSPEKLDRIGEYLYQRASKDIYRKR---YKF------VEIAMEAMD
EFR3B_Ciona_intestin_39       LVRSEMEKLTYYAASAPEKLDRIGDYLARRLTRDMARKR---EMP------VVIAMEALN
Tetraodon_nigrovirid_40       LSKSDMEKLTFYAVSAPEKLDRIGEYLAKRLSHDV----------------------ALD
Solenopsis_invicta_41         LIKNNMEKLTFYSLSSPEKLDRIGEYLFQRASRDIYRRR---NGF------VVIAMEAMD

Cryptococcus_neoform_0        ALLTECKR--DIALFARSTLRVIDSSLDVRVYQRGG-------IDLEV----VGRAAAAF
Drosophila_melanogas_1        LLLQACHAQTTLNLFVESFLRMVQKLLE----DSNP--------NLKI----MA--TNSF
```

```
EFR3A_Equus_caballus_2        QLLMACHSQ-SIKPFVESFLHMVAKLLE----SGEP--------KLQV----LG--TNSF
Neurospora_crassa_3           ALIEKSPK--DLPLFASCVLSILEQVLK----SSDI--------TMVE----SS--IPTF
Debaryomyces_hanseni_4        SLIEECSE--NLNVFASFVCSILKSVLQ----SKDL--------SLCK----HA--IQTY
Saccharomyces_cerevi_5        KIVLHCKE--NLNVFVKDFLYIMNKVLSNNNFNNDV--------SVVE----LI--ELAF
Ustilago_maydis_6             NLLTECKN--SLNYFIKPSQSIIAAALD----AAQPTSARPRDLEISA----RA--ASAF
Ascaris_suum_7                QLLQSCHDLSSLNQFIESFLKMLQKLLE----TNNF--------QMEK----LA--TDSF
Harpegnathos_saltato_8        QLLVACHAQ-TLNLFIESFLKMVQKLLE----STDP--------QLQI----LA--TQSF
Monodelphis_domestic_9        QLLMACHSQ-SIKPFVESFLHMVAKLLE----SGEP--------ELQM----LG--TNSF
Ailuropoda_melanoleu_10       QLLMACHSQ-SIKPFVESFLHMVAKLLE----SGEP--------KLQV----LG--TNSF
Ashbya_gossypii_11            KIVTSCKE--NLNVFVKEFLDIMIKTLS----NNNF--------NLDVCVVEAA--EAVF
Nematostella_vectens_12       LLLMACHAP-SLNLFVESFLRMVQKLLE----SPEA--------ELQV----LG--TSSF
Acyrthosiphon_pisum_13        QLLVACHAQ-TLNLFVESYLRIVQKLLE----SSEP--------SLQI----LA--TQSF
Culex_quinquefasciat_14       LLLMACHAQ-ILNLFVESFLRMVQKLLE----DTNP--------TLQI----MA--TNSF
Aedes_aegypti_15              LLLMACHAQ-ILNLFVESFLRMVQKLME----DVNP--------TLQI----MA--TNSF
Camponotus_floridanu_16       QLLVACHAQ-TLNLFIESFLKMVQKLLE----STDP--------QLQI----LA--TQSF
Caenorhabditis_elega_17       QLLQACHSSPSLPQFSENHLRMVQRLLE----SNNA--------KMEQ----LA--TDSF
EFR3B_Homo_sapiens_18         QLLMACHCQ-SINLFVESFLKMVAKLLE----SEKP--------NLQI----LG--TNSF
EFR3A_Homo_sapiens_19         QLLMACHSQ-SIKPFVESFLHMVAKLLE----SGEP--------KLQV----LG--TNSF
EFR3B_Ailuropoda_mel_20       QLLMACHCQ-SINLFVESFLKMVAKLLE----SEKP--------NLQI----LG--TNSF
Schizosaccharomyces__21       TLVQKCRD--NINVMASEVVNML--LVA----SSSK--------NLEV----LSACVDCF
EFR3A_Xenopus_laevis_22       QLLMACHSQ-SIKPFVESFLHMVAKLLE----SGEP--------KLQI----YG--TNSF
EFR3A_Taeniopygia_gu_23       QLLMACHSQ-SIKPFVESFLHMVAKLLE----SGEP--------KLQV----LG--TNSF
EFR3B_Danio_rerio_24          QLLMACHCQ-SINLFVESFLKMVRKLLE----ADKP--------NLQI----LG--TNSF
Anopheles_darlingi_25         LLLMACHAQ-ILNLFVESFLRMVQKLLE----DTNP--------TLQI----MA--TNSF
EFR3B_Mus_musculus_26         QLLMACHCQ-SINLFVESFLKMVAKLLE----SEKP--------NLQI----LG--TNSF
Yarrowia_lipolytica_27        KLIEECHE--DLNLFAQNVVNTLLDVVN----SGDL--------LLCQ----HS--NKVF
EFR3A_Rattus_norvegi_28       QLLMACHSQ-SIKPFVESFLHMVAKLLE----SGEP--------KLQV----LG--TNSF
Gallus_gallus_29              QLLMACHSQ-SIKPFVESFLHMVAKLLE----SGEP--------KLQV----LG--TNSF
Kluyveromyces_lactis_30       KIVENCNE--NMNIFIKDFIHIMTLVLNNNNFNNDP--------TIVG----LI--ERVL
Ornithorhynchus_anat_31       QLLMACHSQ-SIKPFVESFLHMVAKLLE----SGEP--------KLQV----LG--TNSF
EFR3A_Oryctolagus_cu_32       QLLMACHSQ-SIKPFVESFLHMVAKLLE----SGEP--------KLQV----LG--TNSF
Nasonia_vitripennis_33        QLLAACHAQ-TLNLFVESFLKMVQKLLE----STDP--------QLQI----LA--TQSF
Candida_glabrata_34           KIVDHCKE--NLNVFVKEFLTLMNMVLTNNSINNDV--------TVIE----LL--EITF
Ixodes_scapularis_35          QLLVACHAQ-SLNLFVESFLKMVQRLLE----CHNA--------DLQL----LA--TQSF
Daphnia_pulex_36              QLLLACHAQ-SLNLFVESFLKTVQKLLE----TTEP--------ALQI----LA--SQSF
Tribolium_castaneum_37        QLLLACHAP-ALNLFVESFLKMVQKLLE----STEP--------ELQI----LA--TQSF
Anopheles_gambiae_38          LLLMACHAQ-ILNLFVESFLRMVQKLLE----DTNP--------TLQI----MA--TNSF
EFR3B_Ciona_intestin_39       QLLLACHAQ-QINLFVESFLKMVATLLE----SDNP--------EFLT----LG--TNSF
Tetraodon_nigrovirid_40       QLLMACHSQ-SIKPFVESFLHMVAKLLE----SREP--------DLQV----LG--TNSF
Solenopsis_invicta_41         QLLVACHAQ-TLNLFVESFLKMVQKLLE----STDP--------QLQI----LA--TQSF

Cryptococcus_neoform_0        IAYT-TYTDGSAVGVDDT-----LTKTYFEILRKFGSMATVSL-LDSSEKPDTE------
Drosophila_melanogas_1        VKFA-NIN-----EDTPS-----YHRRYDFFISKFSSMCHS-------DAASMRD-----
EFR3A_Equus_caballus_2        VKFA-NIE-----EDTPS-----YHRRYDFFVSRFSAMCHSCH-----SDPEIRT-----
Neurospora_crassa_3           QAFC-ENHDPTSLAADQA-----YFRQYVSVVQQYASLASTR------PAPGKAQ-----
Debaryomyces_hanseni_4        GVLC-SKLDGGLFSGDKV-----FVDSFGSLSQNLINIGSENSKKRGPNNLEWQMI----
Saccharomyces_cerevi_5        SSICQNLD-DVLCNGDME-----FVQLYQNFVDLFFKIVTERI-----HNDDML------
Ustilago_maydis_6             YALASFLD-----PASSV-----VDEGFQRLLRSFALLAVERPLGADATLGEDAE-----
Ascaris_suum_7                VNFA-NIE-----ENTPA-----YHRQYDFFISKFAAMCHSNQ----GEDAK--------
Harpegnathos_saltato_8        VRFA-NIE-----EDTPS-----YHTRYDFFVSKYSAMCHSNN-----DDPAIRK-----
Monodelphis_domestic_9        VKFA-NIE-----EDTPS-----YHRRYDFFVSRFSSMCHSCY-----DDPEVQR-----
Ailuropoda_melanoleu_10       VKFA-NIE-----EDTPS-----YHRRYDFFVSRFSAMCHSCH-----SDPEIRT-----
Ashbya_gossypii_11            SSIC-QHLDGVLCNSDLE-----FTQMYRSFVDVYCQVVTERL-----HNDE--------
Nematostella_vectens_12       VKFA-NIE-----EDTPS-----YHRRYDFFVSKFSAMCWNDN-----ENQKHRQ-----
Acyrthosiphon_pisum_13        VRFS-NIE-----EDTPS-----YHRRYDFFVSKFSSMCHNNN-----PNLVTME-----
Culex_quinquefasciat_14       VRFA-NIE-----EDTPS-----YHRRYDFFISKFSSMCYGNN-----DDLELRD-----
Aedes_aegypti_15              VRFA-NIE-----EDTPS-----YHRRYDFFISKFSSMCYGNN-----DDLELRD-----
Camponotus_floridanu_16       VRFA-NIE-----EDTPS-----YHTRYDFFVSKYSAMCHSNN-----DDSIIRK-----
Caenorhabditis_elega_17       VTFS-NIE-----ESSPS-----YHRQYDFFIDKFSQMCHANPQAAYGDDFR--------
EFR3B_Homo_sapiens_18         VKFA-NIE-----EDTPS-----YHRSYDFFVSRFSEMCHSSH-----DDLEIKT-----
EFR3A_Homo_sapiens_19         VKFA-NIE-----EDTPS-----YHRRYDFFVSRFSAMCHSCH-----SDPEIRT-----
EFR3B_Ailuropoda_mel_20       VKFA-NIE-----EDTPS-----YHRSYDFFVSRFSEMCHSSH-----DDMEIKT-----
Schizosaccharomyces__21       ATFCDNSG-----KGSPATFGNEFHSAFNNLVNSFFELSKGID----CVDPQ--------
EFR3A_Xenopus_laevis_22       VKFA-NIE-----EDTPS-----YHRRYDFFVSRFSAMCHSCH-----DDPEVRK-----
EFR3A_Taeniopygia_gu_23       VKFA-NIE-----EDTPS-----YHRRYDFFVSRFSAMCHSCD-----PDPEIQN-----
```

```
EFR3B_Danio_rerio_24         VKFA-NIE-----EDTPS-----YHRSYDFFVSRFSEMCHSGY-----EDPDIRT-----
Anopheles_darlingi_25        VRFA-NIE-----EDTPS-----YHRRYDFFISKFSSMCYGNN-----DDVELRD-----
EFR3B_Mus_musculus_26        VKFA-NIE-----EDTPS-----YHRSYDFFVSRFSEMCHSSH-----DDLEIKT-----
Yarrowia_lipolytica_27       ALFC-QYHDGGLFLGDPE-----FVRSFKQLLEVYVNMAKVPN-----GPNSVQW-----
EFR3A_Rattus_norvegi_28      VKFA-NIE-----EDTPS-----YHRRYDFFVSRFSAMCHSCH-----SDPEIRT-----
Gallus_gallus_29             VKFA-NIE-----EDTPS-----YHRRYDFFVSRFSAMCHSCD-----HDPETQT-----
Kluyveromyces_lactis_30      EAIC-NHLDGSLVSGDSE-----FLELFKNFVTLYFKVANTKL-----NDTDLV------
Ornithorhynchus_anat_31      VKFA-NIE-----EDTPS-----YHRRYDFFVSRFSAMCHSCH-----ADPETRT-----
EFR3A_Oryctolagus_cu_32      VKFA-NIE-----EDTPS-----YHRRYDFFVSRFSAMCHSCH-----SDPEIRT-----
Nasonia_vitripennis_33       VRFA-NIE-----EDTPS-----YHTRYDFFVSKYSSMCHSNN-----DDSTVRK-----
Candida_glabrata_34          GTICRNLD-GAYYGGDTE-----FIKMFKSFVDLLFEVVSKRL---------NND-----
Ixodes_scapularis_35         VKFA-NIE-----EDTPS-----YHRRYDFFVSKFSSLCHDNN-----PDAELRK-----
Daphnia_pulex_36             VKFA-NIE-----EDTPS-----YHRRYDFFVSKFASLCHSNH-----PEVDVLN-----
Tribolium_castaneum_37       VKFA-NIE-----EDTPS-----YHRRYDFFVSKFSAMCHSSE-----GD---------
Anopheles_gambiae_38         VRFA-NIE-----EDTPS-----YHRRYDFFISKFSSMCYGNN-----DDMELRD-----
EFR3B_Ciona_intestin_39      EKFS-EIK-----EDTAS-----YHRRYDFFVSKFSSMCHSQQ-----KNSAIRQ-----
Tetraodon_nigrovirid_40      VKFA-NIE-----EDTPS-----YHRRYDFFVSQFSAMCHSTH-----EDPETMTRSGLR
Solenopsis_invicta_41        VRFA-NIE-----EDTPS-----YHTRYDFFVSKYSAMCHSSN-----DDPTTRK-----

Cryptococcus_neoform_0       ----------------QQNRTRLI----ALAGLNGAA----TSDAIFASTRDFPRQIDLI
Drosophila_melanogas_1       ----------------SLRLAGIK----GLQGVIRKT----VSDDLVEN-IWEAEHMEKI
EFR3A_Equus_caballus_2       ----------------EIRIAGIR----GIQGVVRKT----VNDELRAT-IWEPQHMDKI
Neurospora_crassa_3          ----------------HSKPIALRWRNAGLEAIRSVA----SSDALSSM---VARQYDIL
Debaryomyces_hanseni_4       ----------------------------SLMTCRY------ISNCLGYNSKFSKKFIEIC
Saccharomyces_cerevi_5       ----------------------------LKCCIDIS----NTNSVSSN-PQLNHFVSKS
Ustilago_maydis_6            ----------------QRNRTRLI----GLGALAGAV----ASDAIYSS-NFK-QLLSLL
Ascaris_suum_7               ----------------AIRYAGLR----GLRGVLWKS----ATDPLQAS-IWEKQHMDKI
Harpegnathos_saltato_8       ----------------QIRLAGIQ----GLQGVVRKT----LSDDLVEN-IWEPVHMDKI
Monodelphis_domestic_9       ----------------EIRVAGIR----GIQGVVRKT----VNDELQAT-IWEPQHMDKI
Ailuropoda_melanoleu_10      ----------------EIRIAGIR----GIQGVVRKT----VNDELRAT-IWEPQHMDKI
Ashbya_gossypii_11           -------------------L--------LMKGCLDIS----KTANLAGN-PGVSSLISRG
Nematostella_vectens_12      ----------------QIRTSGLR----GLQGVVRKT----VSDDLQVN-LWDNTHISKI
Acyrthosiphon_pisum_13       ----------------MIRMAGIK----GIQGVIRKT----VSDDLVEN-IWEPVHMDKI
Culex_quinquefasciat_14      ----------------SIRMAGIK----GLQGVIRKT----VSDDLVEN-IWEKQHMEKI
Aedes_aegypti_15             ----------------SIRMAGIK----GLQGVIRKT----VSDDLVEN-IWEKQHMEKI
Camponotus_floridanu_16      ----------------QIRLAGIQ----GLQGVVRKT----LSDDLVEN-IWEPVHMDKI
Caenorhabditis_elega_17      ----------------LARCAGLR----GLRGVVWKS----VTDDLHPN-IWEQQHMDKI
EFR3B_Homo_sapiens_18        ----------------KIRMSGIK----GLQGVVRKT----VNDELQAN-IWDPQHMDKI
EFR3A_Homo_sapiens_19        ----------------EIRIAGIR----GIQGVVRKT----VNDELRAT-IWEPQHMDKI
EFR3B_Ailuropoda_mel_20      ----------------KIRMSGIK----GLQGVVRKT----VNDELQAN-IWDPQHMDKI
Schizosaccharomyces__21      ----------------QSKML-------GLKAFHALTACKFAGTEGGMR--YQPHFAIHC
EFR3A_Xenopus_laevis_22      ----------------EIRIAGIR----GIQGVVRKT----VNDELRAT-IWEPQHMDKI
EFR3A_Taeniopygia_gu_23      ----------------EIRIAGIR----GIQGVVRKA----VNDELRAT-IWEPQHMDKI
EFR3B_Danio_rerio_24         ----------------KIRMAGIK----GLQGVVRKT----VNDELQAN-IWDPQHMDKI
Anopheles_darlingi_25        ----------------SIRMAGIK----GLQGVIRKT----VSDDLVAN-IWEKQHMEKI
EFR3B_Mus_musculus_26        ----------------KIRMSGIK----GLQGVVRKT----VNDELQAN-IWDPQHMDKI
Yarrowia_lipolytica_27       ----------------KIV---------GLEAAKSIAGSAAIATQTGST------SISPI
EFR3A_Rattus_norvegi_28      ----------------EIRIAGIR----GIQGVVRKT----VNDELRAT-IWEPQHMDKI
Gallus_gallus_29             ----------------EIRIAGIR----GIQGVVRKT----VNDELRAT-IWEPQHMDKI
Kluyveromyces_lactis_30      -----------------------------LKGCLDFS----KISNLGSIHQWSAT-AKNC
Ornithorhynchus_anat_31      ----------------EIRIAGIR----GIQGVVRKT----VNDELRAT-IWEPQHMDKI
EFR3A_Oryctolagus_cu_32      ----------------EIRIAGIR----GIQGVVRKT----VNDELRAT-IWEPQHMDKI
Nasonia_vitripennis_33       ----------------QIRLAGIQ----GLQGVVRKT----VSDDLVEN-IWNMVHMDKI
Candida_glabrata_34          ----------------DLM----------LKVCIDISTIIGIASDPQLN-YLVPKCVETA
Ixodes_scapularis_35         ----------------QLRLAGLR----GLQGVVRKT----VSDDLQVN-IWDETHMEKI
Daphnia_pulex_36             ----------------SLRLAGLR----GIQGVVRKT----VSDDLVEN-IWEPVHMDKI
Tribolium_castaneum_37       ----------------QIRIAGIK----GLQAVVRKT----VSDDLVEN-IWEPVHMDKI
Anopheles_gambiae_38         ----------------SIRMAGIK----GLQGVIRKT----VSDDLVAN-IWEKQHMEKI
EFR3B_Ciona_intestin_39      ----------------KVQLHGVR----GLRGVIRKT----VTDELQVN-IWEKQHMDKI
Tetraodon_nigrovirid_40      LGLGLGSLLTSLAANVRIRVAGIR----GLQGVVRKT----VNDELQAI-IWEPQHMDKL
Solenopsis_invicta_41        ----------------QIRLAGIQ----GLQGVVRKT----LSDDLVEN-IWEPVHMDKI

Cryptococcus_neoform_0       IPPLLVNTFEGQISE---------------LKLESAKIGMDASPSP--FFSEFAAKGPVA
Drosophila_melanogas_1       VPSLLFNMQF-----CVNV-----------MFVKKN----LLASGD-------LTPVEDA
EFR3A_Equus_caballus_2       VPSLLFNMQK--------I-----------EEADSR----IGPPSS-------PSAA--D
```

```
Neurospora_crassa_3          VPMILENLWTENEDFLDVLLQRVQGDNNVEDAPLLRRRTSNATAQP-------SETTGGE
Debaryomyces_hanseni_4       IPILTETVHA--NNKQSNLLTILKSNVNIEDENHHLGRIASTKTNQ-------TSRKAQQ
Saccharomyces_cerevi_5       VAYTISKFQERNPKFKTLSLEAALE-----SNLGKR----LSRTQT-------RTIGLDK
Ustilago_maydis_6            TPALVENAKANRVTLDWLRS----------ESNKAT----EGEPTYAEFNIAKKPLAIRR
Ascaris_suum_7               VPSILFNLQD-----DDSL-----------ESEDTAETVMNAPFLD-------QPYAVDG
Harpegnathos_saltato_8       VPSLLYNMQN--------S-----------RYADKE----QATPDS-------PT----E
Monodelphis_domestic_9       VPSLLFNMQK--------L-----------EDVDSR----IGPPSS-------PDG---D
Ailuropoda_melanoleu_10      VPSLLFNMQK--------T-----------EEVDSR----AGPPPS-------PSAS--D
Ashbya_gossypii_11           VELTLAKFQEVQPRFQGES-----------LQVDLHSSEKRLSRSQ-------THLTAAE
Nematostella_vectens_12      VPSLLFILEE----------------------------------SE-------KSFNEFQ
Acyrthosiphon_pisum_13       VPSLLFNMHT------------------------------MNGISE-------EV----N
Culex_quinquefasciat_14      VPSLLFNMQS--------------------------VSGS----KSVDQE-------------A
Aedes_aegypti_15             VPSLLYNMQS--------S---------------------SGSKIV-------DQE---A
Camponotus_floridanu_16      VPSLLYVDKE------------------------------HAMPDS-------PT----E
Caenorhabditis_elega_17      VPSILFNLQE--------------------PDDSGKGFSSSQIPKFDNTFADSTQSHRVD
EFR3B_Homo_sapiens_18        VPSLLFNLQH--------V-----------EEAESR----SPSPLQ-------APE---K
EFR3A_Homo_sapiens_19        VPSLLFNMQK--------I-----------EEVDSR----IGPPSS-------PSAT--D
EFR3B_Ailuropoda_mel_20      VPSLLFNLQH--------I-----------EEAESR----SPSPLQ-------APE---K
Schizosaccharomyces__21      V--LINSWSESDQEKKSFI-----------DLVRSTAKSYKPLPPS-------STGVSLP
EFR3A_Xenopus_laevis_22      VPSLLFNMQK--------I-----------EDTDSR----TGPPAS-------PTTG--D
EFR3A_Taeniopygia_gu_23      VPSLLFNMQK--------I-----------EDIDSR----TGPPSS-------PTVG--V
EFR3B_Danio_rerio_24         VPSLLFNLQS--------G-----------EGTESR----SPSPLQ-------ASE---K
Anopheles_darlingi_25        VPSLLFNMQS-------------------------------GPSKS-------TDTE--T
EFR3B_Mus_musculus_26        VPSLLFNLQH--------V-----------EEAESR----SPSPLQ-------APE---K
Yarrowia_lipolytica_27       VHLLLSSLQE----SQSDL-----------EQLDHSLDLGGGLPDN-------IGGSKRN
EFR3A_Rattus_norvegi_28      VPSLLFNMQK--------I-----------EEVDSR----LGPPSS-------PSAA--D
Gallus_gallus_29             VPSLLFNMQK--------I-----------EDVDSR----TGPPSS-------PTGG--E
Kluyveromyces_lactis_30      VSIALTKFQERHPIYSE-------------ATIDSSFSEPGSPALKKKLTRTQTKVMGLD
Ornithorhynchus_anat_31      VPSLLFNMQK--------I-----------EDNDSR----IGPPSS-------PPGG--D
EFR3A_Oryctolagus_cu_32      VPSLLFNMQK--------I-----------EEVDSR----LGPPSS-------PSAA--D
Nasonia_vitripennis_33       VPSLLYNMQN--------A-----------RYSNKE----NATPES-------PT----E
Candida_glabrata_34          IDQLQARYPQFKENSLLEQ-----------PSLTKR----LSKTQT-------RAQEVLE
Ixodes_scapularis_35         VPSLLFNMQD--------A---------------------RWTPDS-------------P
Daphnia_pulex_36             IPSLLYNMQH--------A---------------------SGHPEE------QTDSENGA
Tribolium_castaneum_37       VPSLLFNMQN--------A-----------RFLNNE------AKEV-------IP----E
Anopheles_gambiae_38         VPSLLFNMQS------------------------------GPSKST-------DTE---A
EFR3B_Ciona_intestin_39      IPSLLYSMQ---------------------DDPDSS----LTEEAS-------PGNN--K
Tetraodon_nigrovirid_40      IPSMLFNMQD--------G-----------EDMD-R----AGHPST-------PSVAGQD
Solenopsis_invicta_41        VPSLLYNMQN--------S-----------RYADKE----DATPDS-------PT----E

Cryptococcus_neoform_0       Q-----------RRAPSL-HAHIPGEKGPTSADVVSAALRSLH---SLLQQCNVTQASQI
Drosophila_melanogas_1       T-----------NVTPPA------------------LAEEVLR---ELVGRASFGHIRSV
EFR3A_Equus_caballus_2       K-----------EENPAV------------------LAENCFR---ELLGRATFGNMNNA
Neurospora_crassa_3          P-----------GPNPIAFLGTAVDVDKLAEEDIGVLAMQCLR---QVFVAPSRSQTHNP
Debaryomyces_hanseni_4       D-----------LDNDAV--------------KDSDLNEEALRGLKALFSTSLTSQISEA
Saccharomyces_cerevi_5       A-----------AEDNHD------------------LSVKALQ---SYFNTTETDKLNLS
Ustilago_maydis_6            T-----------RSISAH----VAGEKGPSSEDVISAAIGTLR---GLLRHADAAQVQSI
Ascaris_suum_7               V-----------ADSPKT------------------LSDQFLR---ELMAKAPFGLI-SV
Harpegnathos_saltato_8       E-----------RSDPPQ------------------FAETCMR---ELIGRASFGHIRCV
Monodelphis_domestic_9       K-----------EENPAV------------------LAENCFR---ELLGRATYGNMSNA
Ailuropoda_melanoleu_10      K-----------EENPAV------------------LAENCFR---ELLGRATFGNMNNA
Ashbya_gossypii_11           E-----------SRVLGG------------------YPEQALQ---SYFSTTETDKLSIA
Nematostella_vectens_12      S-----------DDDPAH------------------IAEACLR---ELMSRASFGNIKSV
Acyrthosiphon_pisum_13       D-----------KTDSFS------------------LAESCLR---ELIGRASFGHVKSV
Culex_quinquefasciat_14      T-----------PSTPPV------------------LAEAVLR---ELVSRASFGHIRAV
Aedes_aegypti_15             T-----------PSTPPV------------------LAEAVLR---ELVSRASFGHIRAV
Camponotus_floridanu_16      E-----------RSDPPQ------------------FAETCMR---ELIGRASFGHIRCV
Caenorhabditis_elega_17      D-----------EATPKV------------------LSDRCLR---ELMGKASFGSLRAV
EFR3B_Homo_sapiens_18        E-----------KESPAE------------------LAERCLR---ELLGRAAFGNIKNA
EFR3A_Homo_sapiens_19        K-----------EENPAV------------------LAENCFR---ELLGRATFGNMNNA
EFR3B_Ailuropoda_mel_20      E-----------KENPAE------------------LAERCLR---ELLGRAAFGNIKNA
Schizosaccharomyces__21      E-----------HDEAGD-------------NIDVAIKKLCIRILFNIYTQTDPLFMAES
EFR3A_Xenopus_laevis_22      K-----------EENPGI------------------LAENCFR---ELLGRATYGNMNNA
EFR3A_Taeniopygia_gu_23      K-----------EENPSV------------------LAENCFR---ELLGRATYGNMNNA
EFR3B_Danio_rerio_24         E-----------KESPAE------------------LTERCFR---ELLGRAAYGNIKNA
```

```
Anopheles_darlingi_25         T-----------PSTPPL------------------LAEAVLR---ELVSRASFGHIKSV
EFR3B_Mus_musculus_26         E-----------KENPAE------------------LAERCLR---ELLGRAAFGNIKNA
Yarrowia_lipolytica_27        SMHQDAIDNFIHEESPEK--------------QVRYLSFHALR---TFFDTTSVVQIQSA
EFR3A_Rattus_norvegi_28       K-----------EENPAV------------------LAESCFR---ELLGRATFGNMNNA
Gallus_gallus_29              K-----------EENPAL------------------LAENCFR---ELLGRATYGNMNNA
Kluyveromyces_lactis_30       D-----------VSNTGD------------------YSILALN---TFFNTTETDKLTIG
Ornithorhynchus_anat_31       K-----------EENPAV------------------LAENCFR---ELLGRATYGNMNNA
EFR3A_Oryctolagus_cu_32       K-----------EENPAV------------------LAESCFR---ELLGRATFGNMNNA
Nasonia_vitripennis_33        E-----------RSDPPQ------------------FAETCMR---ELVGRASFGHIRCV
Candida_glabrata_34           I-----------PTADND------------------LCVATLH---NYFNTTETDKLNLS
Ixodes_scapularis_35          Q-----------VESPLS------------------LAENCFR---EVMGRATYGHIASV
Daphnia_pulex_36              R-----------NIDPSS------------------LAENCLR---ELVGRASFGNIRSV
Tribolium_castaneum_37        D-----------QTDPPM------------------LAETCLR---ELVGRASFGHIRAV
Anopheles_gambiae_38          T-----------PSTPPL------------------LAEAVLR---ELVSRASFGHIKSV
EFR3B_Ciona_intestin_39       N-----------NVNHSA------------------LAEEALR---ELFSRATFANVSAA
Tetraodon_nigrovirid_40       G-----------EENPAT------------------LAENCFR---ELLGRAAYGNMNNA
Solenopsis_invicta_41         E-----------RSDPPQ------------------FAETCMR---ELIGRASFGHIRCV

Cryptococcus_neoform_0        IDRLVMFLDKHG----------------WQY------AERDCFVAEQVTAWIP--LQYRF
Drosophila_melanogas_1        LKPLLTHLDRHE---------------LWVP------NTFAIHTFRIVMISIQ--PQYSY
EFR3A_Equus_caballus_2        VRPVFAHLDHHK---------------LWDP------NEFAVHCFKIIMYSIQ--AQYSH
Neurospora_crassa_3           TIALLRFIEERVDQNEQVVKRD----AHGKD------NGWAIKVFLMAARWAP--VADRF
Debaryomyces_hanseni_4        TRAIVKN-NYAT---------------TIDP-------IWGCTFLEMCTTWIP--VQLRF
Saccharomyces_cerevi_5        IRTLLRCLQSTP----------------------------NKELLEFVCNGIP--VQLRY
Ustilago_maydis_6             VQNVIAWLDNKSALSIPVPKDGRQLVSHWDD------PEWCCWLAESLCSWTS--LQYRF
Ascaris_suum_7                LEPVLKHCDLHK---------------KWEPP-----ATFAVTTFRAIMYSI----KDPY
Harpegnathos_saltato_8        IRPVLRHLDNHQ---------------LWVP------NYFAIHTFRIIMFSIQ--SQYSY
Monodelphis_domestic_9        VRPVFEHLDHHH---------------LWDP------NEFAVHCFKVIMYSIQ--AQYSH
Ailuropoda_melanoleu_10       VRPVFAHLDHHK---------------LWDP------NEFAVHCFKIIMYSIQ--AQYSH
Ashbya_gossypii_11            IRALIKRL-------------------LEVP---------NKELLQYIANTIP--VQLRY
Nematostella_vectens_12       INPVLNHLNSSE---------------LWVP------NEFALKVFKIIMFSVQ--NQYNY
Acyrthosiphon_pisum_13        IRPVLKHLDAHH---------------MWVP------NVFATQCFRILMFSIQ--SQYSY
Culex_quinquefasciat_14       LKPLLMHMDNHK---------------LWVP------NRFAIDTFRIVMISIQ--PQYSY
Aedes_aegypti_15              LKPLLMHLDNHK---------------LWVP------NRFAIDTFRIVMISIQ--PQYSY
Camponotus_floridanu_16       IRPVLRHLDNHQ---------------LWVP------NYFAIHTFRIIMFSIQ--SQYSY
Caenorhabditis_elega_17       IEPVLKHMDLHK---------------RWTPP-----PSFAIHVFRAIIYSIQ--SQNSY
EFR3B_Homo_sapiens_18         IKPVLIHLDNHS---------------LWEP------KVFAIRCFKIIMYSIQ--PQHSH
EFR3A_Homo_sapiens_19         VRPVFAHLDHHK---------------LWDP------NEFAVHCFKIIMYSIQ--AQYSH
EFR3B_Ailuropoda_mel_20       IKPVLIHLDNHS---------------LWEP------KVFAIRCFKIIMYSIQ--PQHSH
Schizosaccharomyces__21       TKSLIHFFAAKS----------------DTPVN----LEYISVVLNQILDWTP--VELRH
EFR3A_Xenopus_laevis_22       VKPVFAHLDHHK---------------LWES------NEFAVSCFKIIMYSIQ--AQYSH
EFR3A_Taeniopygia_gu_23       VRPVFAHLDHHR---------------LWDP------NEFAVSCFKIIMYSIQ--AQYSH
EFR3B_Danio_rerio_24          VTPVLMHLDNHS---------------LWEG------KTFAVRCFKIIMYSIQ--SQHSH
Anopheles_darlingi_25         LKPLLTHLDQHK---------------LWVP------NRFAIDTFRIVMISIQ--PQYSY
EFR3B_Mus_musculus_26         IKPVLIHLDNHS---------------LWEP------KVFATRCFKIIMYSIQ--PQHSH
Yarrowia_lipolytica_27        TRAIVTYIINSN-----------------VP------EHWATSLVVIVAKWAP--VQYRF
EFR3A_Rattus_norvegi_28       VRPVFAHLDHHK---------------LWDP------NEFAVHCFKIIMYSIQ--AQYSH
Gallus_gallus_29              VRPVFAHLDHHK---------------LWDP------NEFAVSCFKIIMYSIQ--AQYSH
Kluyveromyces_lactis_30       LHALIEHL-------------------LETP---------NKELLQFICNGIP--VQLRY
Ornithorhynchus_anat_31       VRPVFAHLDHHK---------------LWDP------NEFAVHCFKIIMYSIQ--AQYSH
EFR3A_Oryctolagus_cu_32       VRPVFAHLDHHK---------------LWDP------NEFAVHCFKIIMYSIQ--AQYSH
Nasonia_vitripennis_33        IKPVLKHLDNHQ---------------LWVP------NYFAIHTFRIIMFSIQ--SQYSY
Candida_glabrata_34           IRSLIKKLQSTP----------------------------NKELLEFISNDIP--VQLRY
Ixodes_scapularis_35          IKPVLKHLDNHR---------------LWVP------NTFAIYTFKIIIISVS--NVESY
Daphnia_pulex_36              IKPVFKHLDLHE---------------LWVP------NDFAIYTFRVIMYSIQ--VQYSY
Tribolium_castaneum_37        LKPVLKHFDLHK---------------LWAPEDNPNNNEFAIHTFRIIMFSIQ--AQYSY
Anopheles_gambiae_38          LKPLLTHLDHHK---------------LWVP------NKFAIDTFRIVMISIQ--PQYSY
EFR3B_Ciona_intestin_39       LVPALSHMDNHS---------------LWGPK-----NGFAIKCFKVIMYSIQ--AQYSH
Tetraodon_nigrovirid_40       VRPVLVHLDNHH---------------LWEP------NEFAVSCFRIIMYSIQ--AQHSH
Solenopsis_invicta_41         IRPVLRHLDNHQ---------------LWVP------NYFAIHTFRIIMFSIQVHSQYSY

Cryptococcus_neoform_0        IVPTRLVEVLMDLQDRTPTPKHTSAL--------AM----VTTILNSTTSLV--------
Drosophila_melanogas_1        TVVETLMQ-HLDNNFKSSPKTRT-SLAV------VL----SKIIAIAAGESV--------
EFR3A_Equus_caballus_2        HVIQEILG-HLDARKKDSPRVRA-GIIQ------VL----LEAVAIAAKGSI--------
Neurospora_crassa_3           TILLTAIE-VLTQRPLTDDNLRHHNTQA------AM----ISALLRSDVNLI--------
```

```
Debaryomyces_hanseni_4        ITLSTVLS-SLTSLSNKSTKKTSNYPIQ------LLYANYCLGLVSSDVNMI--------
Saccharomyces_cerevi_5        IVILLLVRQLSDKDKNVNPIVSL-----------KL----MSSLLVSDVSIV--------
Ustilago_maydis_6             VVLDTLVD-HLAENGEGKATNKHLSLIQ-------M----SRTILTGQLSLI--------
Ascaris_suum_7                FVIQALIN-HLENMSSSNASVRI-GIAT------VL----SSIVSIA-GTSI--------
Harpegnathos_saltato_8        TVVEALMT-HLDDHSKSSPKIRT-SIAD------TL----SKIISIAAGESV--------
Monodelphis_domestic_9        HVIQETLA-HLDVRKKDSPRVRA-GIIQ------VL----LEAVAIAAKGSI--------
Ailuropoda_melanoleu_10       HVIQEILG-HLDARKKDSPRVRA-GIIQ------VL----LEAVAIAAKGSI--------
Ashbya_gossypii_11            ILVLVFTH-ALNQEDEHA------VVLL------KL----MTTLLVSDVSII--------
Nematostella_vectens_12       VVIQMLLS-YLDASSKESAKKKA-GVVS------VL----AECVGLATGSSI--------
Acyrthosiphon_pisum_13        AVVETLMT-HLDENGNASPKIRT-SIAD------VL----SKIIAIAANESV--------
Culex_quinquefasciat_14       TVVETLMS-HLDQNLASSPKTRT-SLAV------VL----SKIIAIAAGESV--------
Aedes_aegypti_15              TVVETLMS-HLDQNLASSPKTRT-SLAV------VL----SKIIAIAAGESV--------
Camponotus_floridanu_16       TVVEALMT-HLDDHSKSSPKIRT-SIAD------TL----SKIISIAAGESV--------
Caenorhabditis_elega_17       FVIQELIN-HLDSMCSADASTRI-GIAT------VL----SSIVSIA-GTSI--------
EFR3B_Homo_sapiens_18         LVIQQLLG-HLDANSRSAATVRA-GIVE------VL----SEAAVIAATGSV--------
EFR3A_Homo_sapiens_19         HVIQEILG-HLDARKKDAPRVRA-GIIQ------VL----LEAVAIAAKGSI--------
EFR3B_Ailuropoda_mel_20       LVIQQLLS-HLDANSRSAATVRA-GIVE------VL----SEAAVIAATGSV--------
Schizosaccharomyces__21       SIFFCCLR-CLSSSRIVNSETNV--MVP------YM----IYSILNSKNSIS--------
EFR3A_Xenopus_laevis_22       HVIQQILV-HLDLHKKDSPRIRA-GIVQ------VL----LEAVAIAAKGSI--------
EFR3A_Taeniopygia_gu_23       HVIQEILG-HLDVRKRDSPRVRA-GIIQ------VL----LEAVAIAAKGSI--------
EFR3B_Danio_rerio_24          LVIQQLLG-HLDANSKSSATVRA-GIVE------VL----LEVAAIAASGSV--------
Anopheles_darlingi_25         TVVETLMS-HLDQNLTSSPKTRT-SLAV------VL----SKIIAIAAGESV--------
EFR3B_Mus_musculus_26         LVIQQLLS-HLDANSRSAATVRA-GIVE------VL----SEAAIIAATGSV--------
Yarrowia_lipolytica_27        VILVSLVE-MLVAMSPNNVKSEL--VVA------AL----IHALLSSTVNMV--------
EFR3A_Rattus_norvegi_28       HVIQEILG-HLDARKKDSPRVRA-GIIQ------VL----LEAVAIAAKGSI--------
Gallus_gallus_29              HVIQEILG-HLDACKKDPPRVRA-GIIQ------VL----LEAVAIAAKGSI--------
Kluyveromyces_lactis_30       IVILLFVR-PLGTSSEKNMLLIL-----------KL----ISSLLTSAVSII--------
Ornithorhynchus_anat_31       HVIQETLG-HLDARKKDSPRVRA-GIVQ------VL----LEAVAIAAKGSI--------
EFR3A_Oryctolagus_cu_32       HVIQEILG-HLDACKKDSPRVRA-GIIQ------VL----LEAVAIAAKGSI--------
Nasonia_vitripennis_33        TVVEALMT-HLDEHSQSSPKIRT-SIAD------TL----SKIISIAAGESV--------
Candida_glabrata_34           IVVLLLTR-QVSNYERNQVSGSQ-GNPDGALNSLKL----ISCLLVSKISIV--------
Ixodes_scapularis_35          EITSALTQEDGGERLSRGPKVRM-GIVN------VL----FHLVNIAAGESV--------
Daphnia_pulex_36              AVVENLMM-HLDTSSRSRAKVRT-SMAN------VL----AKIISIAAGESV--------
Tribolium_castaneum_37        TVVETLVA-HLDDNAKSSPIIRT-SISG------VL----SKIIAIAAGESV--------
Anopheles_gambiae_38          TVVETLMS-HLDQNLTSSPKTRT-SLAV------VL----SKIIAIAAGESV--------
EFR3B_Ciona_intestin_39       VVVKMLLD-HLDENTKEDDDIKA-SMLQ------VL----CIIVPIPSSAAI--------
Tetraodon_nigrovirid_40       HVIQQVLN-HLDTHSRDPPRVRA-GIVQ------VL----LETVAIAAKGSVAGVLGNHS
Solenopsis_invicta_41         TVVEALMT-HLDDHSKSSPKIRT-SIAD------TL----SKIISIAAGESV--------

Cryptococcus_neoform_0        ---------------------------------------GLGVTDLLQHLVSLIIRRIHF
Drosophila_melanogas_1        ---------------------------------------GPSALDIINNLLTHLRTSVS-
EFR3A_Equus_caballus_2        ---------------------------------------GPTVLEVFNTLLKHLRLSVEF
Neurospora_crassa_3           ---------------------------------------GLSVMDVLLNLLRHMQRLVQM
Debaryomyces_hanseni_4        ---------------------------------------GLSISDVIQQI---LFLQANL
Saccharomyces_cerevi_5        ---------------------------------------GLSVLDIMRKLLNFQLKNAT-
Ustilago_maydis_6             ---------------------------------------GLSTSDTLSNLCALAVRRVYK
Ascaris_suum_7                ---------------------------------------GPSLLGIFNSLLKHLRQSVEF
Harpegnathos_saltato_8        ---------------------------------------GPSVLEIINSLLSHLRVSVT-
Monodelphis_domestic_9        ---------------------------------------GPTVLEVFNTLLKHLRLSVEF
Ailuropoda_melanoleu_10       ---------------------------------------GPTVLEVFNTLLKHLRLSVVF
Ashbya_gossypii_11            ---------------------------------------GLSVLDLLRKIINFQLASAT-
Nematostella_vectens_12       ---------------------------------------GPAVLEVFNTLLRHLRYSVDA
Acyrthosiphon_pisum_13        ---------------------------------------GPTVLEIINSLLTNLRTSVTR
Culex_quinquefasciat_14       ---------------------------------------GPSALDIINNLLTHLKTSVS-
Aedes_aegypti_15              ---------------------------------------GPSALDIINNLLTHLKTSVS-
Camponotus_floridanu_16       ---------------------------------------GPSVLEIINSLLSHLRVSVT-
Caenorhabditis_elega_17       ---------------------------------------GPLLLSIFNSLLKHLRTSVDF
EFR3B_Homo_sapiens_18         ---------------------------------------GPTVLEMFNTLLRQLRLSIDY
EFR3A_Homo_sapiens_19         ---------------------------------------GPTVLEVFNTLLKHLRLSVEF
EFR3B_Ailuropoda_mel_20       ---------------------------------------GPTVLEMFNTLLRQLRLSIDY
Schizosaccharomyces__21       ---------------------------------------GLSVIDVLRDLGSHLINAVNS
EFR3A_Xenopus_laevis_22       ---------------------------------------GPTVLEVFNTLLKHLTLSVDF
EFR3A_Taeniopygia_gu_23       ---------------------------------------GPTVLEVFNTLLKHLRISVDF
EFR3B_Danio_rerio_24          ---------------------------------------GPTVLEVFNTLLRHLRLSVDY
Anopheles_darlingi_25         ---------------------------------------GPSALDIINNLLMHLKTSVS-
```

```
EFR3B_Mus_musculus_26          ---------------------------------------GPTVLEMFNTLLRQLRLSIDY
Yarrowia_lipolytica_27         ---------------------------------------GLSVMDILGSLLNKQAAIIKH
EFR3A_Rattus_norvegi_28        ---------------------------------------GPTVLEVFNTLLKHLRLSVEL
Gallus_gallus_29               ---------------------------------------GPTVLEVFNTLLKHLRISVDF
Kluyveromyces_lactis_30        ---------------------------------------GLSVIDILRRLI---------
Ornithorhynchus_anat_31        ---------------------------------------GPTVLEVFNTLLKHLRLSVEL
EFR3A_Oryctolagus_cu_32        ---------------------------------------GPTVLEVFNTLLKHLRLSVEF
Nasonia_vitripennis_33         ---------------------------------------GPSVLEIISSLLTHLRVSVS-
Candida_glabrata_34            ---------------------------------------GLSVLDIMRKVLAVQLKSKES
Ixodes_scapularis_35           ---------------------------------------GPSVLEVFHSLLNHLRHSIDM
Daphnia_pulex_36               ---------------------------------------GPSVLEIVNSLLKHLKQSIMS
Tribolium_castaneum_37         ---------------------------------------GPMVLEIINSLLCHLRDSVKH
Anopheles_gambiae_38           ---------------------------------------GPSALDIINNLLMHLKTSVST
EFR3B_Ciona_intestin_39        ---------------------------------------GPSVLDVFNRISKHLHLCAD-
Tetraodon_nigrovirid_40        DGRRTQRPVDARRAEVCPPEDGVRQEHLGGPAGGVVVSPGPTVLEVFNTLLKHLRVSVDL
Solenopsis_invicta_41          ---------------------------------------GPSVLEIINSLLSHLRVSVT-

Cryptococcus_neoform_0         DLRD----------------------ALLPSLVQCVSSLGTHIYYADQINDIVEELALRI
Drosophila_melanogas_1         ----------------TTSEITPEESQYQEALINALGEFANHHPDYQKIEIMLF-IMNTV
EFR3A_Equus_caballus_2         EANDLQGGSVVSANLNASS-KDNDEKIVQNAIIQTIGFFGSNLPDYQRSEIMMF-IMGKV
Neurospora_crassa_3            PGDPDS-MRVEDETIGTDEGAIGQRRELLFRLQQCVGDLATHVYYADQISDMIQTILLKL
Debaryomyces_hanseni_4         LLSQ------------SHYFQEEDVKKLSMIYSNCICNLSTHIYYFDQVPDSIREILIKV
Saccharomyces_cerevi_5         ----------------------NKEVVAQSCI--TMTDLNHKTYYAEQTSDMLYELLLKL
Ustilago_maydis_6              DTRD----------------------SLLPPLVDCISGLGTHVYYADQINDIVEEISGRI
Ascaris_suum_7                 QQSK-------------DCPSVEQEKIYQETLINAMGDYANALPDYQKVEIMMF-TVGNI
Harpegnathos_saltato_8         ----------------RNQSSSSDEQLYQEALINALGEFANHLPDYQKIEIMMF-IMSKV
Monodelphis_domestic_9         EAGERR-GSMGSVNVSTST-KDNDERIVQNAIIQTIGFFGSNLPDYQRSEIMMF-IMGKV
Ailuropoda_melanoleu_10        EANDLQGGSGGSANLNSSS-KDNDEKIVQNAIIQTIGFFGSNLPDYQRSEIMMF-IMGKV
Ashbya_gossypii_11             ------------------------SPQVTEACTNTISALNRKTYYKDQPIDMISELLLKL
Nematostella_vectens_12        RFNS-----------SGHGCKLEDEMDFEQSIVHTIGAFAALLPDFQKTDVMLF-IMDKF
Acyrthosiphon_pisum_13         RPPS-----------NTGLSESDGENEYREALIHALGEFAAHLPDYQKIEIMMF-IMSKV
Culex_quinquefasciat_14        ----------------TQHESTPEETQYQEALINALGEFANHHPDYQKIEIMLF-IMNTV
Aedes_aegypti_15               ----------------TQHESTPEETQYQEALINALGEFANHHPDYQKIEIMLF-IMNTV
Camponotus_floridanu_16        ----------------RNQSSSNDEQLYQEALINALGEFANHLPDYQKIEIMMF-IMSKV
Caenorhabditis_elega_17        ERSG-------------KCSDQPAEKMYQEALINAMGDFANALPDYQKVEMMMF-TVGNI
EFR3B_Homo_sapiens_18          ALTG---SYDGAVSLGTKIIKEHEERMFQEAVIKTVGSFASTLPTYQRSEVILF-IMSKV
EFR3A_Homo_sapiens_19          EANDLQGGSVGSVNLNTSS-KDNDEKIVQNAIIQTIGFFGSNLPDYQRSEIMMF-IMGKV
EFR3B_Ailuropoda_mel_20        ALTG---SYDGAISLGTKIIKEHEERMFQEAVIKTIGSFASTLPTYQRSEVILF-IMSKV
Schizosaccharomyces__21        FDADDQTWY----NMCESPSEKLPRRLYL--LLVCITCLTKHQYYDEEFADVWR-EIDTL
EFR3A_Xenopus_laevis_22        ELGDRR-SSAGSAVFSSSSTRESDERIVQNAIIQTIGFFGSNLPDYQRSEIMMF-IMGKV
EFR3A_Taeniopygia_gu_23        ELSDRR-SSLESTTLSISS-KESDERIVQNAIIQTIGFFGSNLPDYQRSEIMMF-IMSKV
EFR3B_Danio_rerio_24           ELTG---SYD-CTNIGTKIIKEHEERQLQEAVIRTIGSFANTLPTYQRSEVMLF-IMGKV
Anopheles_darlingi_25          ----------------TQHESASEETQYQEALINALGEFANHHPDYQKIEIMLF-IMNTV
EFR3B_Mus_musculus_26          ALTG---SYDGAVSLGSKIIKEHEECMFQEAVIKTIGSFASTLPTYQRSEVILF-IMSKV
Yarrowia_lipolytica_27         AQSN--------------NEASGAFEELIDKLSACMISLGTHIYYADQISDMVGEVLWRC
EFR3A_Rattus_norvegi_28        EASDSQKGSVGSVSLSS---KDNDEKIVQNAVIQTIGFFGSNLPDYQRSEIMMF-IMGKV
Gallus_gallus_29               ELGDRR-SSAGSATFSTSS-KENDERIVQNAIIQTIGFFGSNLPDYQRSEIMMF-IMGKV
Kluyveromyces_lactis_30        ----------------TVQLAKSDSTTVVKQIAVTIKDLNRKTYYKQQSSDMFAELSFKF
Ornithorhynchus_anat_31        ESGDAR-SSVGSASFSTSS-KDNDERIVQNAIIQTIGFFGSNLPDYQRSEIMMF-IMGKV
EFR3A_Oryctolagus_cu_32        EANDLQGGPAGSASLNTSS-KDNDEKIVQNAIIQTIGFFGSNLPDYQRSEIMMF-IMGKV
Nasonia_vitripennis_33         ----------------RNQSSTNDEQLYQEALINALGEFANHLPDYQKIEIMKF-IMSKV
Candida_glabrata_34            -------------------------MPLVHQCRVTIKDLNNKIYYSEQTSDMLYDLVVKI
Ixodes_scapularis_35           HSEE----------------ELEDEHQFQEAVIGTLGEFANNLPDYQKIEIMMF-ILGKV
Daphnia_pulex_36               AIPV------------DDAEAVADEKQFQESLISALAEFAYHLPDYQKIEIMMF-IIGRT
Tribolium_castaneum_37         GTSM------------------TEEKQYQDALINALGEFANHLPDYQKIEIMMF-IMSKI
Anopheles_gambiae_38           QHDE----------------STPEETQYQEALINALGEFANHHPDYQKIEIMLF-IMNTV
EFR3B_Ciona_intestin_39        ---------------------SKPHSKFEEALIETTGVLAAVVPDYQKLETMTF-YVNRA
Tetraodon_nigrovirid_40        ELGDGS-RRNSAASVSSAR-RESEERIVQNAIIQTIGFFGGNLPDYQRAEVMMF-IMGKV
Solenopsis_invicta_41          ----------------RNQSSSSDEQLYQEALINALGEFANHLPDYQKIEIMMF-IMSKV

Cryptococcus_neoform_0         -AE-----------------------------IPSSD----------TAR------SEII
Drosophila_melanogas_1         -PD-----------------------------LSKK-----------SKG------DQML
EFR3A_Equus_caballus_2         -PV-----------------------------FGTSTHTLDISQLG-DLG------TRRI
Neurospora_crassa_3            RPS-----------------------------RPTSPPNSSPNGERSDNGAAE---DQPL
Debaryomyces_hanseni_4         --------------------------------VSILENSVISGNANSSGV------YRFI
```

```
Saccharomyces_cerevi_5        --------------------------------KSDTVKDVEKNAVV-EDI------DFLV
Ustilago_maydis_6             -AALQMPESISDAASVKASNNLGAVHRGQQQDLSSHLHRQRLANAGPEQR------DESI
Ascaris_suum_7                -PK-----------------------------LSEDGRALK-------PG------DAFL
Harpegnathos_saltato_8        -P------------------------------YSQPDRIVSV-----GKG------DVLL
Monodelphis_domestic_9        -PV-----------------------------FGTTTHTLDTSQLG-DLG------TRRI
Ailuropoda_melanoleu_10       -PV-----------------------------FGTSTHTLDISQLG-DLG------TRRI
Ashbya_gossypii_11            -KE--------------------------------------------HPG------QREK
Nematostella_vectens_12       -PH-----------------------------RNTNQHTSDRRSLRFTES------DVEL
Acyrthosiphon_pisum_13        -PQ-----------------------------FKSSNNPN----------------DLHV
Culex_quinquefasciat_14       -PD-----------------------------PSKK-----------NKG------DTLL
Aedes_aegypti_15              -PD-----------------------------PSKK-----------NKG------DQLL
Camponotus_floridanu_16       -P------------------------------YSQPDRIVSV-----GKG------DVLL
Caenorhabditis_elega_17       -PN-----------------------------LDERKS---------KQG------DEFL
EFR3B_Homo_sapiens_18         -P------------------------------RPSLHQAVDTGRTG-ENR------NRLT
EFR3A_Homo_sapiens_19         -PV-----------------------------FGTSTHTLDISQLG-DLG------TRRI
EFR3B_Ailuropoda_mel_20       -P------------------------------LPSLHHPMETGRTG-ENR------NRLT
Schizosaccharomyces__21       --------------------------------ANSET-------------------SSAI
EFR3A_Xenopus_laevis_22       -PV-----------------------------FGSSPHMLDTSQLG-DMG------TKRI
EFR3A_Taeniopygia_gu_23       -PV-----------------------------LGTTSQSLDPSNLG-DLG------PRRI
EFR3B_Danio_rerio_24          -P------------------------------IPGLHPTLPSIGSG-PEG------NRMI
Anopheles_darlingi_25         -PD-----------------------------PSNK-----------SRG------DQLL
EFR3B_Mus_musculus_26         -P------------------------------LPSVHHPVETGRTG-ENR------NRLT
Yarrowia_lipolytica_27        -TD-----------------------------PATAGQTGHAVSADDGTGLQIKRPTRRV
EFR3A_Rattus_norvegi_28       -PV-----------------------------FGTSTHTLDISQLG-DLG------TRRI
Gallus_gallus_29              -PV-----------------------------LGATSQSLDTSHLG-DLG------TRRI
Kluyveromyces_lactis_30       -IE-----------------------------SGKPHH------------------LELF
Ornithorhynchus_anat_31       -PV-----------------------------FGTTTHTLDTSQLG-DLG------TRRI
EFR3A_Oryctolagus_cu_32       -PV-----------------------------FGTSAHTLDISQLG-DLG------TRRI
Nasonia_vitripennis_33        -P------------------------------YGEPDSITSA-----GKG------DVLL
Candida_glabrata_34           -KN-----------------------------VQNEV-------------------EKRI
Ixodes_scapularis_35          -P------------------------------QSQEAESSDPRQ---RNS------EVLL
Daphnia_pulex_36              -PV-----------------------------AGEFDPENVQA-------------ETLL
Tribolium_castaneum_37        -PF-----------------------------PLVDNHTPA---------------DNLL
Anopheles_gambiae_38          -PD-----------------------------PSHK-----------SKG------DHLL
EFR3B_Ciona_intestin_39       -NE-----------------------------VLHNSEDLNE-----TDV------EQNF
Tetraodon_nigrovirid_40       -PV-----------------------------FGTPCHTLDTVKIE--------------
Solenopsis_invicta_41         -P------------------------------YSQPDRIVSV-----GKG------DVLL

Cryptococcus_neoform_0        RVLTCCISGVMIMTD---------------------------------AADNDAESKQGN
Drosophila_melanogas_1        QNIL--LKSLLKVGT-------------------------------------QYST----
EFR3A_Equus_caballus_2        QIML--LRSLLMVTS-------------------------------------GYKA----
Neurospora_crassa_3           ESLF--ALTVAKIAA-------------------LKAIKAILWVASPRTKMSGGHI----
Debaryomyces_hanseni_4        ITLLEDISIIFDLLQ-------------------------------------TKPS----
Saccharomyces_cerevi_5        EHIT--------------------------------------------------------
Ustilago_maydis_6             RVLLFCLQGVLKATHQSSGEIHEAVDVKSNGAEKGKAAAASTDSKIGLAH----------
Ascaris_suum_7                QHVL--VKTLLKVAT-------------------------------------KYKT----
Harpegnathos_saltato_8        QSIL--LKSLLKVGT-------------------------------------KYQT----
Monodelphis_domestic_9        QIML--LRSLLMVTS-------------------------------------GYKA----
Ailuropoda_melanoleu_10       QIML--LRSLLMVTS-------------------------------------GYKA----
Ashbya_gossypii_11            DILISDLQAVLQTVG---------------------------------------------
Nematostella_vectens_12       QKML--LHSLRQVSV-------------------------------------SYNP----
Acyrthosiphon_pisum_13        QRMC--LKSLLMVST-------------------------------------KYSS----
Culex_quinquefasciat_14       QNIL--LKSLLKVGT-------------------------------------QYRT----
Aedes_aegypti_15              QNIL--LKSLLKVGT-------------------------------------QYRT----
Camponotus_floridanu_16       QSIL--LKSLLKVGT-------------------------------------KYQT----
Caenorhabditis_elega_17       QHVL--VKTLLKVAT-------------------------------------KYRT----
EFR3B_Homo_sapiens_18         QIML--LKSLLQVST-------------------------------------GFQC----
EFR3A_Homo_sapiens_19         QIML--LRSLLMVTS-------------------------------------GYKA----
EFR3B_Ailuropoda_mel_20       QIML--LKSLLQVST-------------------------------------GFQC----
Schizosaccharomyces__21       QMVA--LTAFHKLQN-------------------------------------EKLN----
EFR3A_Xenopus_laevis_22       QIML--LRSLLMVTS-------------------------------------GYKA----
EFR3A_Taeniopygia_gu_23       QIML--LRSLLMVTS-------------------------------------GYKA----
EFR3B_Danio_rerio_24          QVML--LKSLRQVTC-------------------------------------GFQT----
Anopheles_darlingi_25         QNIL--LKSLLKVGT-------------------------------------QYRT----
EFR3B_Mus_musculus_26         QIML--LKSLLQVST-------------------------------------GFQC----
```

```
Yarrowia_lipolytica_27        FTTLSANNAELQHSS------------------DESVGAATGGTANGSAGVGSFSHDNSP
EFR3A_Rattus_norvegi_28       QIML--LRSLLMVTS-------------------------------------GYKA----
Gallus_gallus_29              QIML--LRSLLMVTS-------------------------------------GYKA----
Kluyveromyces_lactis_30       QLDL---DSLISVTS---------------------------------------------
Ornithorhynchus_anat_31       QIML--LRSLLMVTS-------------------------------------GYKA----
EFR3A_Oryctolagus_cu_32       QIML--LRSLLMVTS-------------------------------------GYKA----
Nasonia_vitripennis_33        QSIL--LKSLLKVGT-------------------------------------KYQT----
Candida_glabrata_34           LVDD--MKYIVD------------------------------------------------
Ixodes_scapularis_35          QHIL--LKSLLTVGQ-------------------------------------KYRT----
Daphnia_pulex_36              QHML--LKCLLKVSY-------------------------------------KYQS----
Tribolium_castaneum_37        QSIL--LKSLLKVGT-------------------------------------KYQT----
Anopheles_gambiae_38          QNIL--LKSLLKVGT-------------------------------------QYRT----
EFR3B_Ciona_intestin_39       AHLL--LLCLVQIAK-------------------------------------TYHC----
Tetraodon_nigrovirid_40       ------------VTS-------------------------------------GFKS----
Solenopsis_invicta_41         QSIL--LKSLLKVGT-------------------------------------KYQT----

Cryptococcus_neoform_0        NVPQPTPSTPGSPTPPNKGKLPAPAETPFLTPLFEYLRPQAHRSSRRNPISPEVWQ----
Drosophila_melanogas_1        -------------------------------------------VSFEKAFPASFLQ----
EFR3A_Equus_caballus_2        -------------------------------------------KTIVTALPGSFLD----
Neurospora_crassa_3           -------------------------------------------NLSRNRVPIQTWD----
Debaryomyces_hanseni_4        -------------------------------------------NISRNHVSLEDWDLSLI
Saccharomyces_cerevi_5        ----------------------------------------------QPSISLELFI----
Ustilago_maydis_6             -------------------------------------------AGTRNRVAPASIL----
Ascaris_suum_7                -------------------------------------------AYLATIFADSFLS----
Harpegnathos_saltato_8        -------------------------------------------IHLNTTFPPSFLD----
Monodelphis_domestic_9        -------------------------------------------KTIVTALPAPFLD----
Ailuropoda_melanoleu_10       -------------------------------------------KTIVTALPGSFLD----
Ashbya_gossypii_11            ----------------------------------------------QPFMTLELFL----
Nematostella_vectens_12       -------------------------------------------TSLSAVFSPSFMD----
Acyrthosiphon_pisum_13        -------------------------------------------VQMNATFPQSFLD----
Culex_quinquefasciat_14       -------------------------------------------VSFEKAFPVSFLQ----
Aedes_aegypti_15              -------------------------------------------VSFEKAFPVSFLQ----
Camponotus_floridanu_16       -------------------------------------------IHLNTTFPPSFLD----
Caenorhabditis_elega_17       -------------------------------------------AYLATVFTDSFLD----
EFR3B_Homo_sapiens_18         -------------------------------------------NNMMSALPSNFLD----
EFR3A_Homo_sapiens_19         -------------------------------------------KTIVTALPGSFLD----
EFR3B_Ailuropoda_mel_20       -------------------------------------------NNMMSALPSNFLD----
Schizosaccharomyces__21       -------------------------------------------TKKESSAPLSLVT----
EFR3A_Xenopus_laevis_22       -------------------------------------------KTIAAALPPAFLD----
EFR3A_Taeniopygia_gu_23       -------------------------------------------TTISNALPAPFLD----
EFR3B_Danio_rerio_24          -------------------------------------------TNMLTALPNSFLD----
Anopheles_darlingi_25         -------------------------------------------VSFEKAFPLSFLQ----
EFR3B_Mus_musculus_26         -------------------------------------------NNMMSALPSNFLD----
Yarrowia_lipolytica_27        DVLIRRLAIVEGFFRLQNEEQD---------------------MTAHNSVPLSAWD----
EFR3A_Rattus_norvegi_28       -------------------------------------------KTIVTALPGSFLD----
Gallus_gallus_29              -------------------------------------------MTITNALPAPFLD----
Kluyveromyces_lactis_30       ----------------------------------------------DQCLDLDLFG----
Ornithorhynchus_anat_31       -------------------------------------------KTIVTALPPPFLD----
EFR3A_Oryctolagus_cu_32       -------------------------------------------KTIVTALPGSFLD----
Nasonia_vitripennis_33        -------------------------------------------IHLNTTFPPSFLQ----
Candida_glabrata_34           -------------------------------------------DISQPVINVDLLT----
Ixodes_scapularis_35          -------------------------------------------VQLGQAFPASFLH----
Daphnia_pulex_36              -------------------------------------------TNFSTSLPLTFLD----
Tribolium_castaneum_37        -------------------------------------------IHLNSTFPVSFLE----
Anopheles_gambiae_38          -------------------------------------------VSFEKAFPVSFLQ----
EFR3B_Ciona_intestin_39       -------------------------------------------SNISN-LTVTLLE----
Tetraodon_nigrovirid_40       -------------------------------------------KSMAAALPPSFLD----
Solenopsis_invicta_41         -------------------------------------------IHLNTTFPPSFLD----

Cryptococcus_neoform_0        ---------------ETLPLLCEADYSVRSTYARALILFLETEMQRGPTPRT-TPASGGS
Drosophila_melanogas_1        ---------------PLLKMARAPHNPTRMV----VMQILQALL-----DRH--------
EFR3A_Equus_caballus_2        ---------------PLLSPSLMEDYELRQL----VLEVMHNLM-----DRH--------
Neurospora_crassa_3           ---------------GTQWLLRDPDGLVRKA----YVDAVVTWL-----DRETTPA----
Debaryomyces_hanseni_4        LLSSEIGLNPTKDQKPLFSYEQITDIQFK------YLRVFGYFL-----NNEL-------
Saccharomyces_cerevi_5        ---------------DLAH--YMKNHIICLF------NIVETEV-----PSS--------
```

```
Ustilago_maydis_6           ---------------PTASLLASPNHAVRLA----YAQTLITLFRDEFDREQLERESAVF
Ascaris_suum_7              ---------------TLLQLALVADPCVRLD----TQHIFHTLL-----DRH--------
Harpegnathos_saltato_8      ---------------PLLRMSLAADAEMRLL----VQKIFHTLI-----DRH--------
Monodelphis_domestic_9      ---------------PILSPSLMEDYELRQL----VLEIMHNLT-----DRH--------
Ailuropoda_melanoleu_10     ---------------PLLSPSLMEDYELRQL----VLEVMHNLM-----DRH--------
Ashbya_gossypii_11          ---------------ELAPYVPDRLELFSLV----TDKLPGGFV----------------
Nematostella_vectens_12     ---------------AIFHAAIVENPEVRQL----ALEVIFALI-----DRH--------
Acyrthosiphon_pisum_13      ---------------LLLKTLTASEDEIRFI----VLRILHTLL-----DRH--------
Culex_quinquefasciat_14     ---------------PLLKMARASSLPIRVI----VMQIFQQLL-----DRH--------
Aedes_aegypti_15            ---------------PLLKMARASSVAIRVI----VMQIFQQLL-----DRH--------
Camponotus_floridanu_16     ---------------PLLRMSLAADAEMRLL----VQKIFHTLI-----DRH--------
Caenorhabditis_elega_17     ---------------TLLLLALVRDPQVRLA----TQQIFHTLL-----DRH--------
EFR3B_Homo_sapiens_18       ---------------RLLSTALMEDAEIRLF----VLEILISFI-----DRH--------
EFR3A_Homo_sapiens_19       ---------------PLLSPSLMEDYELRQL----VLEVMHNLM-----DRH--------
EFR3B_Ailuropoda_mel_20     ---------------RLLSTALMEDAEIRLF----VLEILISFI-----DRH--------
Schizosaccharomyces__21     ---------------DFLLHSWPKSAE----------------------RLH--------
EFR3A_Xenopus_laevis_22     ---------------PLLSPSLMEDCELRQL----VLEILHNLI-----DRH--------
EFR3A_Taeniopygia_gu_23     ---------------PLLSPSLMEDSELRQL----VLEILHNLI-----DRH--------
EFR3B_Danio_rerio_24        ---------------PMLSFALLEDAEIRLL----VLEILVSLI-----DRH--------
Anopheles_darlingi_25       ---------------PLLKMARAGSIPIRII----VMQIFQQLL-----DRH--------
EFR3B_Mus_musculus_26       ---------------RLLSTALMEDAEIRLF----VLEILISFI-----DRH--------
Yarrowia_lipolytica_27      ---------------GTQYLLNHDSAIVKEA----YVFCFVAFL-----EYE--------
EFR3A_Rattus_norvegi_28     ---------------PLLSPSLMEDYELRQL----VLEVMHNLM-----DRH--------
Gallus_gallus_29            ---------------PLLSPSLMEDSELRQL----VLEILHNLI-----DRH--------
Kluyveromyces_lactis_30     ---------------EFLPYVTDKTQLSKLL----YPEAPHQVI----------------
Ornithorhynchus_anat_31     ---------------PLLSASLMEDYELRQL----VLEILHNLI-----DRH--------
EFR3A_Oryctolagus_cu_32     ---------------PLLSPSLMEDYELRQL----VLEVMHNLM-----DRH--------
Nasonia_vitripennis_33      ---------------PLLRMSLASDFEMRLL----VQKIFHTLI-----DRH--------
Candida_glabrata_34         ---------------ELVP--FMKGSVIQLL------NITEEHI-----SGG--------
Ixodes_scapularis_35        ---------------SLLRMSLAADPSVRLV----VQSILHTLL-----DRH--------
Daphnia_pulex_36            ---------------PLLRTSLGQDAYVRVL----VQEILHCLL-----DRH--------
Tribolium_castaneum_37      ---------------PLLRMSLAPDPEMRLL----VQKILHTLL-----DRH--------
Anopheles_gambiae_38        ---------------PLLKMARAASIPIRII----VMQIFQQLL-----DRH--------
EFR3B_Ciona_intestin_39     ---------------PLLKVAFSAQPGDRLL----AQSLLASLL-----DKN--------
Tetraodon_nigrovirid_40     ---------------PLFSISLMEDGELRQL----VLEILHNII-----DRH--------
Solenopsis_invicta_41       ---------------PLLRMSLAADAEMRLL----VQKIFHTLI-----DRR--------

Cryptococcus_neoform_0      GSETATPNREKGVSFKVTEPTPGETATQTQ--------------SGSGATTPPKRNSRSH
Drosophila_melanogas_1      ----QNEQVLSSVS-VKPYPA---LSQEPP--------------SRSDIIFTHKY-----
EFR3A_Equus_caballus_2      ----DNRAKLRGIR-IIPDVADLKIKREKI--------------CRQDTSFMKKN-----
Neurospora_crassa_3         ----DSLARDESARTTLKNRAAQENNLARRVVSSAS--------ARVDKSANAPR-----
Debaryomyces_hanseni_4      ----VTRNENMETG-AINSIESFNVGGDHL--------------QPDYNNYISDP-----
Saccharomyces_cerevi_5      ----ILFSKLYS---LLRELDSHGVQKEMMEEIF----------DKYGKMALLSG-----
Ustilago_maydis_6           AAAP--------IG---EKVSDAIGVVHAIGAAAHVMCLSKSLSPPAQVLGNLRESPLEL
Ascaris_suum_7              ----DNLSLLQHVP-YVPDVADLQLTVEKC--------------SRADQMFMQKH-----
Harpegnathos_saltato_8      ----RNITKLAKP---TVNVTELDLVIEKA--------------SRPDVIFIRKH-----
Monodelphis_domestic_9      ----DNRAKLRGIR-IIPDVADLKIKREKI--------------SKQDASFMKKN-----
Ailuropoda_melanoleu_10     ----DNRAKLRGIR-IIPDVADLKIKREKI--------------CRQDTSFMKKN-----
Ashbya_gossypii_11          ----MNKFFLFLVA-LESPGEQEKLLDDAF--------------AKSKNFTL--------
Nematostella_vectens_12     ----NNSKNLGLNG-MLKDGKEIQLNVEKC--------------PKQDLMFFQKY-----
Acyrthosiphon_pisum_13      ----NNASKLSNIF-YRVNITKSEINLEKC--------------SRSDTIFIRKH-----
Culex_quinquefasciat_14     ----QNQHLMNLIS--INNYPTLTVEQP----------------SRSDILFTHKY-----
Aedes_aegypti_15            ----QNQHLLNMIS--VTNYPTLTVEQA----------------SRSDILFTHKH-----
Camponotus_floridanu_16     ----RNIMKLAKP---TVNMVELGLVIEKA--------------SRPDVIFIRKH-----
Caenorhabditis_elega_17     ----DNAANLVHLG-YELDVSDVQLTVEKC--------------SRADQMFMRKH-----
EFR3B_Homo_sapiens_18       ----GNRHKFSTIS-TLSDISVLKLKVDKC--------------SRQDTVFMKKH-----
EFR3A_Homo_sapiens_19       ----DNRAKLRGIR-IIPDVADLKIKREKI--------------CRQDTSFMKKN-----
EFR3B_Ailuropoda_mel_20     ----GNRHKFSTIS-TLSDISVLKLKVDKC--------------SRQDTVFMKKH-----
Schizosaccharomyces__21     ----TSQSPFVRIKTAEVFYEFLCFLRPSL--------------INFDTILRKSA--ITS
EFR3A_Xenopus_laevis_22     ----DNRAKLRGIR-IIPDVADLKIKREKI--------------SKQDVNFMKKH-----
EFR3A_Taeniopygia_gu_23     ----DNRAKLRGIR-IIPDVSDLKIKREKI--------------SRQDVSFMKKH-----
EFR3B_Danio_rerio_24        ----DNLPKFSNIS-IISDISVLKLKVDKC--------------SRQDNLFMKKH-----
Anopheles_darlingi_25       ----QNQHLLNVI---SVNNYP-TLTIEQA--------------SRSDILFTHKY-----
EFR3B_Mus_musculus_26       ----GNRHKFSTIS-TLGDISVLKLKVDKC--------------SRQDTVFMKKH-----
Yarrowia_lipolytica_27      ----------------FGNPDRFQIGFGRRG-------------YNQNVAYGFVS-----
```

```
EFR3A_Rattus_norvegi_28       ----DNRAKLRGIR-IIPDVADLKIKREKI--------------CRQDTSFMKKN-----
Gallus_gallus_29              ----DNRAKLRGIR-IIPDVSDLKIKRDKI--------------SRQDVSFMKKH-----
Kluyveromyces_lactis_30       -----FIRFFEKVSTLSKQDTEIAISTSFA--------------YYKAASL---------
Ornithorhynchus_anat_31       ----DNRAKLRGIR-IIPDVADLKIKREKI--------------SRQDASFMKKN-----
EFR3A_Oryctolagus_cu_32       ----DNRAKLRGIR-IIPDVADLKIKREKI--------------CRQDTSFMKKN-----
Nasonia_vitripennis_33        ----KNITKLAKP---TVNIIELDLAIEKA--------------SRPDVIFIRKH-----
Candida_glabrata_34           ----STLSRLFQ---MVRDIEDRNLQ------------------SKAMSIIFDKYKKIIL
Ixodes_scapularis_35          ----GNLDQLRSMPSCEGPLGDLRL--EKC--------------SRQDTVFMRKH-----
Daphnia_pulex_36              ----ENRLKLKKP---CIDICGLNLVFDKC--------------SRADSLFYKNH-----
Tribolium_castaneum_37        ----QNLEKLTKP---TVNVSKLNLVLEKS--------------SRPDLIFINKH-----
Anopheles_gambiae_38          ----QNQHLLNVIN--VSHYPTLTIETP----------------SRSDILFTHKY-----
EFR3B_Ciona_intestin_39       ----KNYGKLKLVS-HQREVESLKLITSTA--------------GKYDGDFLERR-----
Tetraodon_nigrovirid_40       ----DNRAKLRGIR-IIPNVAALKIKREKI--------------TKQDVAFMKKH-----
Solenopsis_invicta_41         ----RNITKLAKP---TVNVVELDLAIEKA--------------SRPDVIFIRKH-----

Cryptococcus_neoform_0        RPSLPL---------NRLQSYT------HLSSFDNV--AT-PLDFAAALRILDAMHMVVP
Drosophila_melanogas_1        GANIMQ---------ALIDSMAL-----S-DRVDAL--TSSFNTAALLIVEMSCNETVQE
EFR3A_Equus_caballus_2        GQQLYR---------HIYLGCKE-----EDNVQKNY--ELLYTSLALITIELANEEVVID
Neurospora_crassa_3           SHFLQL---------LHLAIYDH---------ALQF--VDYENDLVLLHVLLAKLVSQLG
Debaryomyces_hanseni_4        GNFI-----------AHFLGYVD-----KFFSGQSL--INID-NASLLQKVLKNMILILG
Saccharomyces_cerevi_5        ---LNY---------FLENVS-E-----PEYTYYSY--HLQAANFLKLNDYKSQTEYKMQ
Ustilago_maydis_6             LPQIDRINADPGRPGSLSSGGS------GTPAAEST--AALPVDYVAVAQALEHAVTALP
Ascaris_suum_7                VVAFTN---------MLYKSVCLVPERREGDMAEHI--DAILCTMALLCVEVGIDEVIIE
Harpegnathos_saltato_8        GPEIYL---------ALYESLEL-----SSNTVDNV--ESIYTTLALLAIELASEETVLE
Monodelphis_domestic_9        GQQLYR---------HIYLGCKE-----DDNVQKNF--ELLYTSLALITIELANEEVVID
Ailuropoda_melanoleu_10       GQQLYR---------HIYLGCKE-----EDNVQKNY--ELLYTSLALITIELANEEVVID
Ashbya_gossypii_11            LSGLIY---------FLE----------KGNTPSNL--YYCYHTKAARFLEFDDYHSQAQ
Nematostella_vectens_12       HDQIIW---------YLYESASM-----RTNNVSNI--LAIYVTMATISTEMSEMEVISQ
Acyrthosiphon_pisum_13        AQDIYV---------SIYESLEM-----TNNTVENV--EAVYTTLALICIELLSEETVLD
Culex_quinquefasciat_14       GSNILQ---------ALIDAMTQ-----E-NNMEIL--KPTYNTAGLMVVEMACGETVQE
Aedes_aegypti_15              GSNILQ---------AILDGMSQ-----E-NHMEVL--KSSYNTAGLMVVEMACSETVQE
Camponotus_floridanu_16       GPEIYL---------ALYESLEL-----SSNTVENV--ESIYTTLALLAIELASEETILE
Caenorhabditis_elega_17       IGEITY---------MLLRAVALAD---ENDLNKHI--DAVLCTMSLLCI-----ESLIE
EFR3B_Homo_sapiens_18         SQQLYR---------HIYLSCKE-----ETNVQKHY--EALYGLLALISIELANEEVVVD
EFR3A_Homo_sapiens_19         GQQLYR---------HIYLGCKE-----EDNVQKNY--ELLYTSLALITIELANEEVVID
EFR3B_Ailuropoda_mel_20       SQQLYR---------HIYLSCKE-----ETNIQKHY--EALYGLLALISIELANEEVVVD
Schizosaccharomyces__21       FSVLWD---------FIYETSWH-----IERWLKVFSVQSEFYILKLIIQRLYQLYGPVS
EFR3A_Xenopus_laevis_22       GQQLYR---------HIYLGCKE-----DDNVHKNY--ELLYTTLALVTIELANEEVVID
EFR3A_Taeniopygia_gu_23       GQQLYR---------HIYLGCKE-----EDNVQKNF--ELLFTSLALTTIELANEEVVID
EFR3B_Danio_rerio_24          AQHLYR---------HIYLCSKE-----QSSVQPHF--EKLYSLLALISMELANEEVVVD
Anopheles_darlingi_25         GSNILQ---------AIIESMSQ-----E-NHLEVL--KSSYNTIALVIVEMACGETVQE
EFR3B_Mus_musculus_26         SQQLYR---------HIYLSCKE-----ETNIQKHY--EALYGLLALISIELANEEVVVD
Yarrowia_lipolytica_27        HLSMQL---------NKLITNTR-------ATNGDF--LLGNSLLQNIVIRLQQDGVVAT
EFR3A_Rattus_norvegi_28       GQQLYR---------HIYLGCKE-----EDNVQKNY--ELLYTSLALITIELANEEVVID
Gallus_gallus_29              GQQLYR---------HIYLGCKE-----EDNVQKNF--ELLYTSLALITIELANEEVVID
Kluyveromyces_lactis_30       LSGLAY---------YVN----------NNRPSDGY--YA-YHHHASKFLGLADYQTQVE
Ornithorhynchus_anat_31       GQQLYR---------HIYLGCKE-----EDNVQKNF--ELLYTSLALTTIELANEEVVID
EFR3A_Oryctolagus_cu_32       GQQLYR---------HIYLGCKE-----EDNVQKNY--ELLYTSLALITIELANEEVVID
Nasonia_vitripennis_33        GPEIYM---------ALYESLEL-----TNNRVENV--EAIYTTLALLTVELASEETVLE
Candida_glabrata_34           LPGLNY---------FQM-NIKE-----PEYTYYLY--HFNAAKTLGVTSYYSETQQKLD
Ixodes_scapularis_35          GPDILY---------HVYENAQL-----ANNGPANF--QALFTTLALLCVELGSEDVLID
Daphnia_pulex_36              GSRILV---------NLSESLDL-----TNVTRENI--EAIYCTAALVCVELATDEMLVD
Tribolium_castaneum_37        GPIIYD---------ALYTTLQM-----DSNTPDNV--EAVYTTLALLCVELASAETVID
Anopheles_gambiae_38          GSNILQ---------AIIESMSQ-----E-NHLEVL--KSSYNTIALVIVEMACGETMQE
EFR3B_Ciona_intestin_39       LPSIYN---------WLYSSYTL-----ETNTIANF--TSLYKCAAVLCLSIVSHDVIMD
Tetraodon_nigrovirid_40       GQQLYR---------HIYLGCKE-----EDNVHKNF--ELLFTTLAILTIELANEDAIVD
Solenopsis_invicta_41         GPEIYL---------ALYESLEL-----SSNTVENV--ESIYTTLALLAIELASEETVLE

Cryptococcus_neoform_0        VAAL---VTGAPMLLAVDRD-AGNELV-RR----PGDGR------AGAW-----------
Drosophila_melanogas_1        FLLF---ILGIQQVACT-VD-TLGNVH-KC----SLHAI------SIGL-L---------
EFR3A_Equus_caballus_2        LIRL---AIALQDSAIINED-NLPMFH-RC----GIMAL------VAAY-L---------
Neurospora_crassa_3           VNAA---KFGIPMIFRLQED--IQDVE-TPLGKVRIGSL------VHGY-LWTLTEKFDC
Debaryomyces_hanseni_4        INFI---NNFVPFFFYWQLP-LNPNLV-DD----NTRIK-----DTLGYTL---------
Saccharomyces_cerevi_5        TRTL---FTKEDLLSYYSDT-GSNKYS-KK----GAQILLSRDNQISTSDL---------
Ustilago_maydis_6             CSAA---LALTPMLVALDKD-AGSRLT-VH----GATVN------TG-------------
```

```
Ascaris_suum_7               LFRL---SFALQSIAVDSSS-GFKGSK-RI----AVHNM------VARY-L---------
Harpegnathos_saltato_8       LLRL---VLSLQDLALTSGQINLGL---KF----NLHAI------VISL-L---------
Monodelphis_domestic_9       LIRV---AIALQDNAIINED-NLPMFH-RC----AMMAL------VAAY-L---------
Ailuropoda_melanoleu_10      LIRL---AIALQDSAIINED-NLPMFH-RC----GIMAL------VAAY-L---------
Ashbya_gossypii_11           YKRQEREIFTRNDLVNYYSDPGCNRYA-EK----GLRIL---------------------
Nematostella_vectens_12      LVPL---VFGFQNMAMKEES-KISTAC-RC----ALHAV------VAAF-L---------
Acyrthosiphon_pisum_13       FIRL---ILSIQELAITNA--VLSTQQ-KF----HLHGL------VISI-M---------
Culex_quinquefasciat_14      FLLF---VLGVQQVAIT-EA-ELSPKH-RC----NLHSI------AISL-L---------
Aedes_aegypti_15             FLLF---VLSVQQVAVA-EV-ELSPKH-RC----NLHSI------AISI-L---------
Camponotus_floridanu_16      LLRL---VLSLQDLALT-SG-QISLSL-KF----NLHAT------VISL-L---------
Caenorhabditis_elega_17      LFRL---SLALQQLALDSKQ-NFSDAK-RN----CIHNM------VAKY-L---------
EFR3B_Homo_sapiens_18        LIRL---VLAVQDVAQVNEE-NLPVYN-RC----ALYAL------GAAY-L---------
EFR3A_Homo_sapiens_19        LIRL---AIALQDSAIINED-NLPMFH-RC----GIMAL------VAAY-L---------
EFR3B_Ailuropoda_mel_20      LIRL---VLAVQDVAQVNEE-NLPVYN-RC----ALYAL------GAAY-L---------
Schizosaccharomyces__21      VTAV---LPAMYRGLRPFEN-DPP----RC----IAEAV------TAYY-I---------
EFR3A_Xenopus_laevis_22      LIRV---AIALQDIAITNED-NLQMFN-RC----GIMGM------VAAY-L---------
EFR3A_Taeniopygia_gu_23      LIRL---AIALQDIAIINED-NLPMFN-RC----GVMAL------VAAY-L---------
EFR3B_Danio_rerio_24         LIRV---ALALQDLALSSEE-MLPVYN-RC----AIHAL------SSAY-L---------
Anopheles_darlingi_25        FLLF---ILGLQQVAVT-EV-ELSPKH-RC----NLHSI------AISL-L---------
EFR3B_Mus_musculus_26        LIRL---VLAVQDVAQVNEE-NLPTYN-RC----ALYAL------GAAY-L---------
Yarrowia_lipolytica_27       FPHM----LKAHELGRALVT--------GQSSESNTLAQGIIIEDIFTLYL---------
EFR3A_Rattus_norvegi_28      LIRL---AIALQDSAIINED-NLPMFH-RC----GIMAL------VAAY-L---------
Gallus_gallus_29             LIRL---AIALQDVAIINED-NLPMFN-RC----GIMAL------VAAY-L---------
Kluyveromyces_lactis_30      FKRKDNDIFTKEDLLNYYSDAGSNIYS-EK----GRDIL-----------L---------
Ornithorhynchus_anat_31      LIRV---AIALQENAINNED-NLPMFH-RC----GIMAL------TAAY-L---------
EFR3A_Oryctolagus_cu_32      LIRL---VIALQDSAIINED-NLPMFH-RC----GIMAL------VAAY-L---------
Nasonia_vitripennis_33       LLRL---VMSLQDLALT-SI-QMNIAI-KF----NLHST------VISL-L---------
Candida_glabrata_34          NGEL---FTKEELMKYYKNA-NNTQFG-EK----GMQIL-------MSY-D---------
Ixodes_scapularis_35         LLRL---MFAIQELPTTGGA-SLSTAQ-VA----SLHGL------VAAF-L---------
Daphnia_pulex_36             ILRW---ALAVQDVALTNT--MLHPTN-RI----SLHVV------SLSI-F---------
Tribolium_castaneum_37       LLQL---VLGIQSLALNST--TLSTSQ-KF----NLHAL------VISL-L---------
Anopheles_gambiae_38         FLLF---ILGVQQVAVT-EV-ELSPKH-RC----NLHSI------AISL-L---------
EFR3B_Ciona_intestin_39      VTRI---MLAVQSVAVDSIS-NSGMTHFSC----GVLAS------TSCC-L---------
Tetraodon_nigrovirid_40      LIRL---AVALQEMALANEE-NLPMFN-RC----GVMAL------VAAY-L---------
Solenopsis_invicta_41        LLRL---VLSLQDLALTSSQISLPL---KF----NLHAI------VISL-L---------

Cryptococcus_neoform_0       --------------------VLERKRAIRELVSLV--------------------WRRIA
Drosophila_melanogas_1       -------------------VLISRVSGINNLLEYA--------------------QKIVD
EFR3A_Equus_caballus_2       -------------------NFVSQMIAVPAFCQHV--------------------SKVIE
Neurospora_crassa_3          EGTAPGSAIHGEIIRRRSKNFWVEGINIPAPAVDLVGTPGQARP-----------PPQMN
Debaryomyces_hanseni_4       ---------IYHSLIKMDKIYELNYVRESEFFNSL--------------------LGDIN
Saccharomyces_cerevi_5       -------------------LSDSQVRTTPLEYKNV--------------------PNAIF
Ustilago_maydis_6            -----------------------------LENQRRLSSRMVA-------------ARVLA
Ascaris_suum_7               -------------------NLSSQLMAIPSLCQHV--------------------QQVVK
Harpegnathos_saltato_8       -------------------VLISYACNITALMDYA--------------------KKVVE
Monodelphis_domestic_9       -------------------NFLSQMIAVPAFCQHV--------------------SKVIE
Ailuropoda_melanoleu_10      -------------------NFVSQMIAVPAFCQHV--------------------SKVIE
Ashbya_gossypii_11           ---------------------ISQNTRVST-------------------------TDLTE
Nematostella_vectens_12      -------------------ILAAQLLRLETLSDYA--------------------EEVYE
Acyrthosiphon_pisum_13       -------------------LLVAIVTDLHPLKDYV--------------------EKVIE
Culex_quinquefasciat_14      -------------------ILIGRCTGISTLVEYA--------------------EKVIQ
Aedes_aegypti_15             -------------------ILIGRCTGIGPLVEYA--------------------EKVIQ
Camponotus_floridanu_16      -------------------VLISYVCNITVLMDYA--------------------EKVVD
Caenorhabditis_elega_17      -------------------NLSAQLIANPSLCQQV--------------------QHVVS
EFR3B_Homo_sapiens_18        -------------------NLISQLTTVPAFCQHI--------------------HEVIE
EFR3A_Homo_sapiens_19        -------------------NFVSQMIAVPAFCQHV--------------------SKVIE
EFR3B_Ailuropoda_mel_20      -------------------NLISQLTTVPAFCQHI--------------------REVIE
Schizosaccharomyces__21      -------------------IYIGENLKITTLQSSG--------------------RKWLD
EFR3A_Xenopus_laevis_22      -------------------NFLSQMIAVPAFCQHV--------------------SKVIE
EFR3A_Taeniopygia_gu_23      -------------------NFLSQMIAVPAFCQHV--------------------SKVIE
EFR3B_Danio_rerio_24         -------------------NLISQLTTVPAFCQHV--------------------HEVIE
Anopheles_darlingi_25        -------------------ILLARCTAIGTLVEYA--------------------EKLIE
EFR3B_Mus_musculus_26        -------------------NLISQLTTVPAFCQHI--------------------HEVIE
Yarrowia_lipolytica_27       -------------------KQVGDVSDAADLTVQA--------------------GDEVA
EFR3A_Rattus_norvegi_28      -------------------NFVSQMIAVPAFCQHV--------------------SKVIE
```

```
Gallus_gallus_29            -------------------NFLSQMIAVPSFCQHV--------------------SKVIE
Kluyveromyces_lactis_30     -------------------VDHSDQNGTDIDQDTV-------------------------
Ornithorhynchus_anat_31     -------------------NFLSQMIAVPAFCQHV--------------------SKVIE
EFR3A_Oryctolagus_cu_32     -------------------NFVSQMIAVPAFCQHV--------------------SKVIE
Nasonia_vitripennis_33      -------------------VLIANVCNISTLIDYA--------------------NKIVE
Candida_glabrata_34         -------------------NQISN-------------------------------SDLLN
Ixodes_scapularis_35        -------------------FLAAQLTSIPSLNAHV--------------------NEVLK
Daphnia_pulex_36            -------------------NLASHTMNVATLIEYS--------------------AQVVA
Tribolium_castaneum_37      -------------------VLIPSVVTIPPLLEYA--------------------NQIVE
Anopheles_gambiae_38        -------------------ILLGRCTGVGPLVEYV--------------------EKLIQ
EFR3B_Ciona_intestin_39     -------------------LILAHVSAVPEFQHYV--------------------NQVIH
Tetraodon_nigrovirid_40     -------------------NFLSQM--------------------------------VIE
Solenopsis_invicta_41       -------------------VLISYVCNITALMDYATKVGSLKFYDVLLKIKCAMNTKVIE

Cryptococcus_neoform_0      DRWGIVEIDDLANKALAS--------------LPEPYLIP-----PYPVPD--------S
Drosophila_melanogas_1      ARR-----------------------------EEASHFLP------PLLEP--------K
EFR3A_Equus_caballus_2      IRT-----------------------------MEAPYFLP-----EHIFRD--------K
Neurospora_crassa_3         MRD-----------------------------LESEALLP--FDERASLVDCICTGYQEL
Debaryomyces_hanseni_4      YRK-----------------------------LNKLWVNGLQSKEEDAVLD--------N
Saccharomyces_cerevi_5      SNG------------------------------KAVYDNN-----DFAAKQ--------N
Ustilago_maydis_6           KLGETFDTDSVSRAAQGVLSQIPSLGIEPGPSPVGGLTLP-----------SEVVPFSAV
Ascaris_suum_7              ERA-----------------------------VRGHLSL------NLMAEG--------N
Harpegnathos_saltato_8      ARR-----------------------------NDSPHLLP-----DLQSQY--------D
Monodelphis_domestic_9      IRT-----------------------------MEAPYFLP-----EHIFRD--------K
Ailuropoda_melanoleu_10     IRT-----------------------------MEAPYFLP-----EHIFRD--------K
Ashbya_gossypii_11          TPP------------------------------EGELQIP-------------------D
Nematostella_vectens_12     MFK---------------------------------------------------------
Acyrthosiphon_pisum_13      QRKL----------------------------LSATHLLP-----ELSVHN---------
Culex_quinquefasciat_14     ARM-----------------------------EEATYLLP-----PLLDNE--------K
Aedes_aegypti_15            SRT-----------------------------EDAPYLLP-----PLLDNE--------K
Camponotus_floridanu_16     ARR-----------------------------KDSPHLLP-----DLQSQY--------D
Caenorhabditis_elega_17     CRAQ----------------------------RGIPGLNLLLNVKDSPNND--------D
EFR3B_Homo_sapiens_18       TRK-----------------------------KEAPYMLP-----EDVFVE--------R
EFR3A_Homo_sapiens_19       IRT-----------------------------MEAPYFLP-----EHIFRD--------K
EFR3B_Ailuropoda_mel_20     TRK-----------------------------KEAPYMLP-----EDVFVE--------R
Schizosaccharomyces__21     SLS-----------------------------SSLPSSLLNRGSSTHSWEK---------
EFR3A_Xenopus_laevis_22     TRN-----------------------------MDATYFLP-----EVIFRD--------K
EFR3A_Taeniopygia_gu_23     TRS-----------------------------VEAAYFLP-----ETIFKD--------K
EFR3B_Danio_rerio_24        MRQ-----------------------------KEIPYLLP-----EDVFIE--------N
Anopheles_darlingi_25       ARR-----------------------------EEASYLLP-----PLMDND--------K
EFR3B_Mus_musculus_26       TRK-----------------------------KEAPYMLP-----EDVFVE--------K
Yarrowia_lipolytica_27      LRK-------------------------------KLGLWP-------------------N
EFR3A_Rattus_norvegi_28     TRT-----------------------------MEAPYFLP-----EHIFRD--------K
Gallus_gallus_29            TRS-----------------------------LEASYFLP-----ETIFKD--------K
Kluyveromyces_lactis_30     --R-----------------------------YSTPIPLP-----QISIPP--------T
Ornithorhynchus_anat_31     TRT-----------------------------EEAPYFLP-----EHIFKD--------K
EFR3A_Oryctolagus_cu_32     IRT-----------------------------MEAPYFLP-----EHIFRD--------K
Nasonia_vitripennis_33      SRS-----------------------------KEATHLLP-----DLRSQY--------D
Candida_glabrata_34         DRP----------------------------------LSP-------VFSS--------K
Ixodes_scapularis_35        VRR-----------------------------ERAPHLLP-------------------Q
Daphnia_pulex_36            ART-----------------------------AKAPLLLR--------FNE--------G
Tribolium_castaneum_37      ART-----------------------------QEAPHLLP------DLMVH--------Y
Anopheles_gambiae_38        ARK-----------------------------EEASYLLP-----PLMDND--------K
EFR3B_Ciona_intestin_39     ARE-----------------------------KFALQFLP-----KNALDS--------H
Tetraodon_nigrovirid_40     LRS-----------------------------MRAPYLLP-----EHIFRD--------K
Solenopsis_invicta_41       ERR-----------------------------NDSPHLLP-----DLQSQY--------D

Cryptococcus_neoform_0      PP---VFLSLPEEP------------VSFIQHTLEGESSS-TAKPLLDQDTLLDALVKSQ
Drosophila_melanogas_1      KL---AGKTFNLQLPH----------LAIDKLALGECLQN-AGMD-AQRLNTGAPY----
EFR3A_Equus_caballus_2      CM---LPKSLEKHDKN----------LYFLTNKIAESLGG-SGYS-VERLSV--PY----
Neurospora_crassa_3         AT---SPPTSPTTSPG----------RNFTHPMLGSTLSA-TPKD-ETQREV--------
Debaryomyces_hanseni_4      HH--ALGHRDENGTLR----------FNPTKKNLEDFVIG-NAFT-LSWINPHRPLILDI
Saccharomyces_cerevi_5      KFDNSIDDNIEEANDT----------VISDANAKGSIYRF-VAED-ARSWKTMRAT----
Ustilago_maydis_6           GSINQLGESSSSSSPS------------LNGALVINSLASSSKLQSATQLDAATLKRWLE
Ascaris_suum_7              AMGTYNGATSNEEEEHAAVGLEEDASLLFDKNDVAEMLKA-SGKD-VQRFAI--PF----
```

```
Harpegnathos_saltato_8        AA---LTSRLAPA-------------LLVDQTVVSECLKG-AGLD-SGKLQQGSGY----
Monodelphis_domestic_9        CM---LPKSLEKHEKN----------LYFLTNKIAESLGG-SGYN-VEKLTV--PY----
Ailuropoda_melanoleu_10       CM---LPKSLEKHDKN----------LYFLTNKIAESLGG-SGYS-VERLSV--PY----
Ashbya_gossypii_11            IP---LPPTPPTPQQH----------RMFLNSD--------ASVKSLDKMK---------
Nematostella_vectens_12       ------KAGCDYKGIR----------VAYEHQELSGVLAR-T------------------
Acyrthosiphon_pisum_13        -----EVKSSSLLPAG----------VLIEEPELTEALKA-SGID-ITTETG--PV----
Culex_quinquefasciat_14       SA----PSTLNTNLPH----------LMVDKLAIAECLQQ-AGLE-HSRVQTGTPY----
Aedes_aegypti_15              PA----PSTLNTNQPH----------LMIDKLAVAECLQQ-AGLE-HNRVQTGTPYSLNQ
Camponotus_floridanu_16       AA---LTSRLTPA-------------VLVDQTVVSECLKG-VGLD-SGKLQQGSGY----
Caenorhabditis_elega_17       PL---SSSALNSTSQGATTITEEDQTLLFNAEDIAESLKA-SGKD-ATRLFV--PFNFNM
EFR3B_Homo_sapiens_18         PR---LSQNLDGVVIE----------LLFRQSKISEVLGG-SGYN-SDRLCL--PY----
EFR3A_Homo_sapiens_19         CM---LPKSLEKHEKD----------LYFLTNKIAESLGG-SGYS-VERLSV--PY----
EFR3B_Ailuropoda_mel_20       PR---LSQNLDGVVMD----------FLFRQSKISEVLGG-SGYN-SDRLCL--PY----
Schizosaccharomyces__21       -----LTNASTEEV------------MLPLDMVVNELLEK-SPKVFPEDSRL--LF----
EFR3A_Xenopus_laevis_22       CS---LPKSLDKHEKN----------QIFLTNKIAESLGG-SGYN-VEKLSM--PY----
EFR3A_Taeniopygia_gu_23       VN---LPKSLEKDETD----------LFFLTNKIAESLGG-SGYN-VERLSV--PY----
EFR3B_Danio_rerio_24          PK---IPKTLEKLEGD----------VLFQQAKITEVLGG-SGYN-TERLAT--PY----
Anopheles_darlingi_25         SA----PSTLNTNLPH----------LLIDKLAAAECLQQ-AGLE-SNRVQTGTLY----
EFR3B_Mus_musculus_26         PR---LSQNLDGVVIE----------FLFRQSKISEVLGG-SGYN-SDRLCL--PY----
Yarrowia_lipolytica_27        YLDWPVSSSSRIQDAD----------AQIDTSMVAKMKTL-SRSE-VEGLFA--EY----
EFR3A_Rattus_norvegi_28       CM---LPKSLEKHDKN----------LYFLTNKIAESLGG-SGYS-VERLSV--PY----
Gallus_gallus_29              CN---LPKSLEKHEKN----------LFFLTNKIAESLGG-SGYS-VERLSV--PY----
Kluyveromyces_lactis_30       TT---NGIGIKKSSPA-------------NDTYVTRSLKH----N---------P-----
Ornithorhynchus_anat_31       CL---LPKSLEKHEKK----------LFFLTNKIAESLGG-SGYS-VEKLSV--PY----
EFR3A_Oryctolagus_cu_32       CM---LPKSLEKHEKN----------LYFLTNKIAESLGG-SGYS-VERLSV--PY----
Nasonia_vitripennis_33        PH---LPSRIPTA-------------LIVDQTIVTECLKG-VGLE-TGKLQQGSGY----
Candida_glabrata_34           PL----SSPMGLISPQ-----------AQTNTQLNQPMRF-VSDD-VHSWKVSRPS----
Ixodes_scapularis_35          PG---RPASPSGTVPEA---------ALFDRAAVGEALRS-TGHD-TSKLFTQMLY----
Daphnia_pulex_36              SA---EEFPQDGIPPE----------CLLDFNTIADCMKE-AGKDIMGMIPLQGPF----
Tribolium_castaneum_37        PQ----DSEVANKVPH----------LLVDQMAVCENLKS-GGID-PSRLAQTSPYGAGG
Anopheles_gambiae_38          SA----PSTLNTNLPH----------LLVDKLAVAECLQQ-AGLE-CNRVQTGTPY----
EFR3B_Ciona_intestin_39       VN--HKPQKLSPENAEK---------VYFNSSEIYNALKGRSGYN-IEKLTR--PF----
Tetraodon_nigrovirid_40       CAVSPLPDSLEKDDQL----------LLFQSTDMAECLAG-PGYN-VEKLSI--PY----
Solenopsis_invicta_41         TG---LTSRLAPA-------------LLVDQTVVSECLKG-AGLD-SGKLQQGSGY----

Cryptococcus_neoform_0        TVQAAKQMDEAGLKRLFDGKWSVEQAIKDSMERFSSANLRPDDDSHYNAASALL----MS
Drosophila_melanogas_1        ----SLNQT-----DHPGHRHSWVESVSNQL---------TQRNSSADLTVYNG--D-VD
EFR3A_Equus_caballus_2        ----VPQVT-----------------DEDRL---------SRRKSIVDTVSIQV--D-IL
Neurospora_crassa_3           ----PAQFRELMLGDWTREAVLANTQAGSSQTASLNGTNGTHRNTVNNNNRLGV--NGVT
Debaryomyces_hanseni_4        VNNYIPKKE-----------------NNQNVLSDM-----SDESSIIENSSYHT-----S
Saccharomyces_cerevi_5        ----APKVSDLKKTMNEKNIPNNMKRDGSFRGSQ------SVKSRVTNITFLLN--E-LK
Ustilago_maydis_6             RDWNVGIAVDEAFAGSSPYAHSSLGTGSQQA---------SRRPSYTPINGLSAGGNAFV
Ascaris_suum_7                ----VTKGNLLAVGDGSQNDGGSSRAATDKTDNFSGMAN-GAAQSILDESALSI--D--M
Harpegnathos_saltato_8        --------------------------SNNSLQHRHSWVDNAGRNSLADINSGGT--E-LD
Monodelphis_domestic_9        ----VPQVT-----------------DEDRL---------SRRKSIVDTVSIQV--D-IL
Ailuropoda_melanoleu_10       ----VPQVT-----------------DEDRL---------SRRKSIVDTVSIQV--D-IL
Ashbya_gossypii_11            ----SPKVS-----------------DLKRA---------ARGIRVAPSHS-----SLRA
Nematostella_vectens_12       ----------------------------------------SSPESIVSSVSNEL--D---
Acyrthosiphon_pisum_13        ----LAHRRSWV--------------ENTNM---------SMKGSFTDLSNLDL--D---
Culex_quinquefasciat_14       ----SLHQT--------DMSAHRHSWVDTSS---------AARNSIVDANYN----D-IE
Aedes_aegypti_15              TDMSAHRHS-----------------WVDTS---------APRNSIVDANY-----D-AE
Camponotus_floridanu_16       --------------------------SSNSLQHRHSWVDSAGRNSLADINSGGQ--E-LD
Caenorhabditis_elega_17       ----NGRKNDGSGDQWQNDTPNFDSTDGRES---------PSGYKTVGIDDVSV--D--M
EFR3B_Homo_sapiens_18         ----IPQLT-----------------DEDRL---------SKRRSIGETISLQV--E-VE
EFR3A_Homo_sapiens_19         ----VPQVT-----------------DEDRL---------SRRKSIVDTVSIQV--D-IL
EFR3B_Ailuropoda_mel_20       ----IPQLT-----------------DEDRL---------SKRKSIGETISLQV--E-VE
Schizosaccharomyces__21       ----AEQSR-----------------HPKYTDIIQKLKKPSREKSFTSSSEYSL--PFIS
EFR3A_Xenopus_laevis_22       ----VPQVT-----------------DEDRL---------SRRKSIVDTISIQV--D-IL
EFR3A_Taeniopygia_gu_23       ----VPQVT-----------------DEDRL---------SRRKSIVDTVSLQV--D-IL
EFR3B_Danio_rerio_24          ----VPQFT-----------------DEDRL---------SKRKSIGETISLQV--E-VD
Anopheles_darlingi_25         ----ALNQT--------DISAHRHSWVDTHS---------TVRNSVVDSSYNEI--E---
EFR3B_Mus_musculus_26         ----IPQLT-----------------DEDRL---------SKRKSIGETISLQV--E-VE
Yarrowia_lipolytica_27        ----VTSMP-----------------QE------------TRAELFSDYDDVMT----IH
EFR3A_Rattus_norvegi_28       ----VPQVT-----------------DEDRL---------SRRKSIVDTVSIQV--D-IL
Gallus_gallus_29              ----VPQVT-----------------DEDRL---------SRRKSIVDTVSIQV--D-IL
```

```
Kluyveromyces_lactis_30       ----VPNVK-----------------DLKNLV--------SSKKDKSNTKTLRG------
Ornithorhynchus_anat_31       ----VPQVT-----------------DEDRL---------SRRKSIVDTVSIQV--D-IL
EFR3A_Oryctolagus_cu_32       ----VPQVT-----------------DEDRL---------SRRKSIVDTVSIQV--D-IL
Nasonia_vitripennis_33        --------------------------SSSALQHRHSWVDSAGRSSLGDINGGTT--E-LD
Candida_glabrata_34           ----IPKVSDLKKAMKNGSSTKNKPLRGSQ----------SVKSRVTNITFLLS--E--L
Ixodes_scapularis_35          ----LARPS----APSPGVPSDWAQCAGEP----------TVTRSVSDLNSNNV--E-VA
Daphnia_pulex_36              ----NANQR--------------YSWADSSL---------HQPSSFTDLSNLNL--DGSE
Tribolium_castaneum_37        PGVVIGGQR-----------------NSWIE---------TARGSVIDINAPPE----VD
Anopheles_gambiae_38          ----ALNQT--------DISAHRHSWVDTHS---------AVRNSVVDASYNDI--E---
EFR3B_Ciona_intestin_39       ----QANLQ-----------------MDTLS---------SDAQRSIDVESINI--EFSV
Tetraodon_nigrovirid_40       ----VPQVTVLYTVGKILHRCKQTSRDEDRL---------TRRKSFVDTVSLQV--D-IM
Solenopsis_invicta_41         --------------------------SGNSLQHRHSWVD-AGRNSLADINSGGA--E-LD

Cryptococcus_neoform_0        MNNASYQSVNGQ--------------------------RLSRTI---------------D
Drosophila_melanogas_1        SVSSSPGVCKKL---------------------------LAPEF---------------N
EFR3A_Equus_caballus_2        PNSVPSDDVVS----------------------------NTEEI---------------T
Neurospora_crassa_3           SPNGSNSNLRPSSSPTGPNGVQAGRTRKTSIRSNGGGGSPAHSTYRAKAQQPVT-----S
Debaryomyces_hanseni_4        ARQNGFGLGLG----------------------------NANDI----------------
Saccharomyces_cerevi_5        TFSDDANKIKDP--------------------------DEENIV---------------G
Ustilago_maydis_6             NGSNASSASVN----------------------------KNSRI---------------S
Ascaris_suum_7                SIDWSPPESRLA--------------------------SRRNTFFAVSRDRDNVLALPLT
Harpegnathos_saltato_8        SGGSSPGVQKKL---------------------------PGEEL---------------T
Monodelphis_domestic_9        PGSNPIDNMVST---------------------------TTEEI---------------T
Ailuropoda_melanoleu_10       PNSIPSDDVVS----------------------------NTEEI---------------T
Ashbya_gossypii_11            SQSVKSRVTNIT--------------------------FLLNEL---------------N
Nematostella_vectens_12       -GQPSSGGTRPS--------------------------TPMEHI---------------T
Acyrthosiphon_pisum_13        SVNSTPAMQRRF--------------------------SPNEDL---------------S
Culex_quinquefasciat_14       SVSSSPGVQKRS---------------------------LASEF---------------N
Aedes_aegypti_15              SVSSSPGVQKRS--------------------------LPVDF----------------N
Camponotus_floridanu_16       SGGSSPSVQKKL---------------------------PGEEL---------------T
Caenorhabditis_elega_17       SVDWTPPVSRKQ--------------------------SRRNTIFSIVNPPKLNAS---T
EFR3B_Homo_sapiens_18         SRNSPEKEERV----------------------------PAEEI---------------T
EFR3A_Homo_sapiens_19         SNNVPSDDVVS----------------------------NTEEI---------------T
EFR3B_Ailuropoda_mel_20       SRNSPEKEERV----------------------------PAEEI---------------T
Schizosaccharomyces__21       PASDYQQNPLLH---------------------------ATKSLVSI------------H
EFR3A_Xenopus_laevis_22       SGSNTEDKV------------------------------NTEEI---------------T
EFR3A_Taeniopygia_gu_23       SGNNTEDKVD-----------------------------NTEEI---------------T
EFR3B_Danio_rerio_24          SRNSPEKEERT----------------------------PAEEI---------------T
Anopheles_darlingi_25         SVSSSPGVQKRS---------------------------LASEY---------------N
EFR3B_Mus_musculus_26         SRNSPEKEERV----------------------------PAEEI---------------T
Yarrowia_lipolytica_27        SRQQVPDENLIVG----------------------------------------------K
EFR3A_Rattus_norvegi_28       SNSVPSDDVVS----------------------------NTEEI---------------T
Gallus_gallus_29              PGNNTEDKID-----------------------------NTEEI---------------T
Kluyveromyces_lactis_30       SQSVKSKVTNIT--------------------------FLLDEL---------------K
Ornithorhynchus_anat_31       PGNTADDTVS-----------------------------STEEI---------------T
EFR3A_Oryctolagus_cu_32       PNSIPPDDVAN----------------------------NTEEI---------------T
Nasonia_vitripennis_33        SAGSSPGVQRKL---------------------------PGEEL---------------T
Candida_glabrata_34           RSTTAGDDSHII--------------------------DPDEEE---------------V
Ixodes_scapularis_35          SVNSSPGFVRKH---------------------------PEEEI---------------T
Daphnia_pulex_36              SNTSSPGVSRRY---------------------------AEEEV---------------T
Tribolium_castaneum_37        SVSSSPGVQRKM---------------------------VEEEL---------------T
Anopheles_gambiae_38          SVSSSPGVQKRS---------------------------LASEY---------------N
EFR3B_Ciona_intestin_39       DKQQSVVMEDGT--------------------------MSAENI---------------T
Tetraodon_nigrovirid_40       SSSLPDKSQ------------------------------LAEEI---------------T
Solenopsis_invicta_41         SGGSSPGVQKKL---------------------------PGEEL---------------T

Cryptococcus_neoform_0        VTDLRDALGGRVDTVSTSGAPSIASFDDSFHS--QSAPRSSL-------------QSRRM
Drosophila_melanogas_1        FDAMKRAL---AEP-----TEAAKREQRERQM--QIVR--TF-------------REGEF
EFR3A_Equus_caballus_2        FEALKKAI----DT-----SG-MEEQEKEKRR--LVIE--KF-------------QKAPF
Neurospora_crassa_3           VEQLKAVLSGHLQPPPTSHGINFQHSDDSDDS--LVSY--DMAPSELSFNPAASGSRQGS
Debaryomyces_hanseni_4        -SSIHSGLLFHQSQNVNMKNAFSNGNETSNGS--------IY-------------TSDRQ
Saccharomyces_cerevi_5        LDKIDVAR---SNS-----LRLAPISSLSDRS---SIG-----------------NRKSF
Ustilago_maydis_6             MQAPSTGV---ND---------LREALGTAAS--L--------------------SSKQA
Ascaris_suum_7                VEALRHMI---SAP-----VDAGEEERREQERTHEILE--KF-------------RNRPF
Harpegnathos_saltato_8        FESMKRIL---TEN-----NN-NHTVEEEKRI--QLSE--FF-------------RNAPF
```

```
Monodelphis_domestic_9        FEALKKAI----DT-----NG-MEEQEKEKRR--LVIE--KF-------------QKAPF
Ailuropoda_melanoleu_10       FEALKKAI---VDT-----SG-MEEQEKEKRR--LVIE--KF-------------QKAPF
Ashbya_gossypii_11            NESQETGI---YDPEEEEVVG-LEKTDLARSISAKVSPAIAY-----------SSKRFGN
Nematostella_vectens_12       FQILKGII--------------SDRQHVNGTN--IESP--KF-------------AESSF
Acyrthosiphon_pisum_13        FEAMKRSL---LDN-----PV-VRVEQNTRQT--QLCD--MF-------------RNTSF
Culex_quinquefasciat_14       FESMKRVL---AEP-----TEASKREAREKQA--AIGR--TF-------------RETAF
Aedes_aegypti_15              FESMKRVL---AEP-----TEANKREAREKQA--AIGR--TF-------------RETAF
Camponotus_floridanu_16       FESMKRIL---TEN-----NN-NYIVEEEKRM--QLSQ--FF-------------RNAPF
Caenorhabditis_elega_17       VDDLKAYA---NAT-----FDPIEEGRKEKELTGSILS--EI-------------RNTDF
EFR3B_Homo_sapiens_18         YETLKKAI---VDS-----VA-VEEQERERRR--QVVE--KF-------------QKAPF
EFR3A_Homo_sapiens_19         FEALKKAI----DT-----SG-MEEQEKEKRR--LVIE--KF-------------QKAPF
EFR3B_Ailuropoda_mel_20       YETLKKAI---VDS-----VA-VEEQERERRR--QVVE--KF-------------QKAPF
Schizosaccharomyces__21       QQSQRGEM-----------VSTLKQALSRPHTASTVV------------------RSPSE
EFR3A_Xenopus_laevis_22       FESLKKAI----DN-----TG-MEEQEKEKRR--LVIE--KF-------------QKAPF
EFR3A_Taeniopygia_gu_23       FEALKKAI----DN-----SG-LEEQEKEKRR--LVIE--KF-------------QKAPF
EFR3B_Danio_rerio_24          FETLKNAI---VDS-----VG-VEEQEKERRR--QVVE--KF-------------QKAPF
Anopheles_darlingi_25         FESMKRVL---AEP-----TEATKREAREKQM--QIGR--AF-------------RETAF
EFR3B_Mus_musculus_26         YETLKKAI---VDS-----VA-VEEQERERQR--QVVE--KF-------------QKAPF
Yarrowia_lipolytica_27        ARSLKQLN-----------AG-------------SILE------------------RQTM
EFR3A_Rattus_norvegi_28       FEALKKAI----DT-----NG-MEEQEKEKRR--LVIE--KF-------------QKAPF
Gallus_gallus_29              FEALKKAI----DN-----NG-LEEQEKEKRR--LVIE--KF-------------QKAPF
Kluyveromyces_lactis_30       NDGDEIKI---ADPDEEDIIG-MEKQDLARSYSLRMNTISST-------------NSRTL
Ornithorhynchus_anat_31       FEALKKAI----DT-----NG-LEEQEKEKRR--LVIE--KF-------------QKAPF
EFR3A_Oryctolagus_cu_32       FEALKKAI----DT-----SG-MEEQEKEKRR--LVIE--KF-------------QKAPF
Nasonia_vitripennis_33        FDSMKRIL---TET-----NN-TQILEEEKRA--QLSQ--LF-------------RNAPF
Candida_glabrata_34           VGIDKMEI---ARS-----LS-----GRGRNS--VVVE--DV-----------STNRASF
Ixodes_scapularis_35          VESLKKLM---QEP-----PP-SQEAQEERRL--AVLD--RF-------------RHTPF
Daphnia_pulex_36              FEALKKVL---AEP-----AESRREAEYEKRQ--VICE--KF-------------RNTPF
Tribolium_castaneum_37        FESMKKVL---TES-----VEIRKEAEARRRK--QLSD--TF-------------RTATF
Anopheles_gambiae_38          FESMKRVL---AEP-----TEASKREAREKQM--QIGR--TF-------------RETAF
EFR3B_Ciona_intestin_39       YEYLKQVF---YKP-----PMSKEERLAKQKK---LAD--HI-------------RSAPF
Tetraodon_nigrovirid_40       FETLKKAI----DT-----TG-LEEQERERRR--QVME--KF-------------QKAPF
Solenopsis_invicta_41         FESMKRIL---TEN-----NN-NQVVEEEKRM--QLSQ--FF-------------RNAPF

Cryptococcus_neoform_0        NKDS----------DVKEILKDIFKDKK-----------------------KGTKAP---
Drosophila_melanogas_1        DDLMRRTEPKHD--LIQNRLNELFNSL------------------------AVERQI---
EFR3A_Equus_caballus_2        EEIAAQCESKAN--LLHDRLAQILELTV-----------------------RPPPSP---
Neurospora_crassa_3           PGNTSQAASSPPRRTSQDRTQQLKF--------------------------GGPLVP---
Debaryomyces_hanseni_4        YNTPRVSDLKDS--VLMKKKPGV-------------------------------------
Saccharomyces_cerevi_5        LQKTATGENQND--DFKDANEDLHSL----------------------------------
Ustilago_maydis_6             NGTTQDGSLSTDRRASRRES-------------------------------RRGP-----
Ascaris_suum_7                DELVEHVHNENEETDLSRTLHRLMQRS---------------------------------
Harpegnathos_saltato_8        QDLVSKTQPKHD--VLQSKLSEIFNTL------------------------SVDPRN---
Monodelphis_domestic_9        EEIAAQCETKAN--FLHDRLAQILELTI-----------------------RPPPSP---
Ailuropoda_melanoleu_10       EEIAAQCESKAN--LLHDRLAQILELTI-----------------------RPPPSP---
Ashbya_gossypii_11            LTSSLDLELQED--AFQDASGEIE------------------------------------
Nematostella_vectens_12       NEIADIASYQLQ--EFNSKLDNVLEAAL-----------------------IPLTTS---
Acyrthosiphon_pisum_13        QDLVAISTASKDEESLHSKITDVFNKV---------------------------------
Culex_quinquefasciat_14       EDLVRRTEPKHD--VIQDKLNEIFNSLS---------------------TERQIGSCAGQ
Aedes_aegypti_15              EDLVRRTEPQHD--VLQNKLNEIFNSLS-----------------------AERQIN---
Camponotus_floridanu_16       QDLVSKTQPKHD--VLQSKLSEIFNTL------------------------SVEPRN---
Caenorhabditis_elega_17       EERVNTNESLNEKSDLSKSIARLLVR----------------------------------
EFR3B_Homo_sapiens_18         EEIAAHCGARAS--LLQSKLNQIFEITI-----------------------RPPPSP---
EFR3A_Homo_sapiens_19         EEIAAQCESKAN--LLHDRLAQILELTI-----------------------RPPPSP---
EFR3B_Ailuropoda_mel_20       EEIAAHCGARAS--LLQSKLNQIFEITI-----------------------RPPPSP---
Schizosaccharomyces__21       INLTRQTSNRVP--LLDMLNLNRA------------------------------------
EFR3A_Xenopus_laevis_22       EEIAAQCESKAN--LLHDKLAQILELTI-----------------------RPPPSP---
EFR3A_Taeniopygia_gu_23       EEIAAQCETKAN--LLHDRLAQILELTI-----------------------RPPPSP---
EFR3B_Danio_rerio_24          EEIAAHCGARAT--MLQSKLNQIFEITI-----------------------RPPPSP---
Anopheles_darlingi_25         EDLVRRTEPKHD--VIQNKLNEIFTALS---------------------AERQISCGIQ-
EFR3B_Mus_musculus_26         EEIAAHCGARAS--LLQSKLNQIFEITI-----------------------RPPPSP---
Yarrowia_lipolytica_27        RDGWLGDDVKNN--KFAEERKGFTPT----------------------------------
EFR3A_Rattus_norvegi_28       EEIAAQCESKAN--LLHDRLAQILELTI-----------------------RPPPSP---
Gallus_gallus_29              EEIAAQCETKAN--LLHDRLAQILELTI-----------------------RPPPSP---
Kluyveromyces_lactis_30       IPSVENAEEHGD--DFRDAHEDIE------------------------------------
```

```
Ornithorhynchus_anat_31       EEIAAQCESKAN--LLHDRLAQILELTI-----------------------RPPPSP---
EFR3A_Oryctolagus_cu_32       EEIAAQCESKAN--LLHDRLAQILELTI-----------------------RPPPSP---
Nasonia_vitripennis_33        HDLIKKTQPKHD--VLQSKLSEIFNAL------------------------MIEPRS---
Candida_glabrata_34           VPATV---NEDD--EFRDAVEDVEAYS---------------------------------
Ixodes_scapularis_35          AELLSCPSGPPQAMDLQNKLKEILG--------------------------KLPGT----
Daphnia_pulex_36              QQLVA-SSAKND--VLNDTLNQILT--------------------------RISIMP---
Tribolium_castaneum_37        SELVRRTQPKHD--VLQNKLNEIFKSL---------------------------------
Anopheles_gambiae_38          EDLVRRTEPKHD--VIQNKLNEIFNALSAE---------------------RQISASCGV
EFR3B_Ciona_intestin_39       EQLLSEMQPDPVD-STQKLVSQLLE-----------------------------------
Tetraodon_nigrovirid_40       EELAAHCESKAN--LLHDRLARIFELTIRSGDPHRLAPEPPALANMCRPPRRPPPSP---
Solenopsis_invicta_41         QDLVSKTQPKHD--VLQSKLSEIFNTL------------------------SVDPRN---

Cryptococcus_neoform_0        -------------KGIVVNRVKSASASE---------------EARTSTGDENGLGMSGM
Drosophila_melanogas_1        -------------TQSDTKSSQLQASNEK--------------PIYETNF----------
EFR3A_Equus_caballus_2        -------------SGTLTITSGHAQYQSV--------------PVYEMKF----------
Neurospora_crassa_3           -------------GEESVVNGAGGQEGSN--------------GAAGNLG-VPISRTTSR
Debaryomyces_hanseni_4        --------------------AFNEHYNSD--------------TTPGSIL----------
Saccharomyces_cerevi_5        -------------------------------------------SSRGKIF----------
Ustilago_maydis_6             -----------------VVTSANGSNGTS--------------AVAILDSLKVGVDEDAS
Ascaris_suum_7                -------------NDTNRLNEYILREKPK--------------NIFELKM----------
Harpegnathos_saltato_8        -------------AVPTTGQQTDTKPSQT--------------PAYEIHF----------
Monodelphis_domestic_9        -------------SGTLTITSGHAQYQSV--------------PVYEMKF----------
Ailuropoda_melanoleu_10       -------------SGTLTITSGHAQYQSV--------------PVYEMKF----------
Ashbya_gossypii_11            --------------------------ASS--------------AFRGKLF----------
Nematostella_vectens_12       -------------ALSISTVSISSVPGD---------------DTFELKF----------
Acyrthosiphon_pisum_13        -------------DISNKLSAVETTNQPI--------------PIYETLF----------
Culex_quinquefasciat_14       LAKLEA-------SAVDGGGAGGKLNQQR--------------AVYENNF----------
Aedes_aegypti_15              -------------CGNQLMTAAGATVQR---------------PIYENNF----------
Camponotus_floridanu_16       -------------TVSTTGQQTDAKPSQT--------------PSYEIHF----------
Caenorhabditis_elega_17       -------------NGEMTRVRDIGRPAKPK-------------NLFEIEL----------
EFR3B_Homo_sapiens_18         -------------SGTITAAYGQPQNHSI--------------PVYEMKF----------
EFR3A_Homo_sapiens_19         -------------SGTLTITSGHAQYQSV--------------PVYEMKF----------
EFR3B_Ailuropoda_mel_20       -------------SGTITAAYGQPQNHSI--------------PVYEMKF----------
Schizosaccharomyces__21       -------------------------MSPT--------------PIQSPPY----------
EFR3A_Xenopus_laevis_22       -------------SGTLTMTAGHAQYQSV--------------PVYEMKF----------
EFR3A_Taeniopygia_gu_23       -------------SGTMTITAGHAHYHSV--------------PVYEMKF----------
EFR3B_Danio_rerio_24          -------------SGTITSSYGQTQSRSV--------------PVYEMKF----------
Anopheles_darlingi_25         -------------AGSLLGANGGLNGPLIDGGKQLGGVGGGQRPIYENNF----------
EFR3B_Mus_musculus_26         -------------SGTISAAYGQPQNHSI--------------PVYEMKF----------
Yarrowia_lipolytica_27        -------------VSDLKGAMGGNAYVSK-------------------EPIAQKFD----
EFR3A_Rattus_norvegi_28       -------------SGTLTVTSGHAQYQSV--------------PVYEMKF----------
Gallus_gallus_29              -------------SGTMTITAGHAHYQSV--------------PVYEMKF----------
Kluyveromyces_lactis_30       --------------------------VSS--------------STRGRLF----------
Ornithorhynchus_anat_31       -------------SGTLTITSGHAQYQSV--------------PVYEMKF----------
EFR3A_Oryctolagus_cu_32       -------------SGTLTITSGHAQYQSV--------------PVYEMKF----------
Nasonia_vitripennis_33        -------------QSIPGGTPSDAKPSQT--------------PAYEIHF----------
Candida_glabrata_34           -------------------------------------------TSRGKIF----------
Ixodes_scapularis_35          -------------LATCGGSQGKLDAVSQ---------DLASAPVYEVNF----------
Daphnia_pulex_36              -------------CGQPELTAVQPNNQSM--------------PLYGNNF----------
Tribolium_castaneum_37        -------------SANDRRNPTEEKKIEK--------------PAYEQNF----------
Anopheles_gambiae_38          QAGSLLLAANGGLNGPSMIEPGKHHLAAG-----------GQRPIYENNF----------
EFR3B_Ciona_intestin_39       --------------------DEDLYVQDY--------------DLESMEY----------
Tetraodon_nigrovirid_40       -------------SGTVSLSAGHGQHQSV--------------PVYEMKF----------
Solenopsis_invicta_41         -------------TVPTTGPQTDTKPSQT--------------PAYEIHF----------

Cryptococcus_neoform_0        TQGDGATGHKVVTNPPLDL-----------------------------------------
Drosophila_melanogas_1        --------------PELFY-----------------------------------------
EFR3A_Equus_caballus_2        --------------PDLCV-----------------------------------------
Neurospora_crassa_3           TQPQATTAHTTLPRPSTSSKRSIKSRAGSRAGPMSSSWLGEKPPAMDLAALLKGIDSASI
Debaryomyces_hanseni_4        --------------SKQMV-----------------------------------------
Saccharomyces_cerevi_5        --------------SST-------------------------------------------
Ustilago_maydis_6             RLGRDTSWSAI---PAKGI-----------------------------------------
Ascaris_suum_7                --------------PASFV-----------------------------------------
Harpegnathos_saltato_8        --------------PELFV-----------------------------------------
Monodelphis_domestic_9        --------------PDLCV-----------------------------------------
```

```
Ailuropoda_melanoleu_10      --------------PDLCV-----------------------------------------
Ashbya_gossypii_11           --------------SS--------------------------------------------
Nematostella_vectens_12      --------------PQEYI-----------------------------------------
Acyrthosiphon_pisum_13       --------------PELFA-----------------------------------------
Culex_quinquefasciat_14      --------------PELFF-----------------------------------------
Aedes_aegypti_15             --------------PELFF-----------------------------------------
Camponotus_floridanu_16      --------------PELFV-----------------------------------------
Caenorhabditis_elega_17      --------------PS-FA-----------------------------------------
EFR3B_Homo_sapiens_18        --------------PDLCV-----------------------------------------
EFR3A_Homo_sapiens_19        --------------PDLCV-----------------------------------------
EFR3B_Ailuropoda_mel_20      --------------PDLCV-----------------------------------------
Schizosaccharomyces__21      --------------VRT-------------------------------------------
EFR3A_Xenopus_laevis_22      --------------PDLCV-----------------------------------------
EFR3A_Taeniopygia_gu_23      --------------PDLCV-----------------------------------------
EFR3B_Danio_rerio_24         --------------PDLCV-----------------------------------------
Anopheles_darlingi_25        --------------PELFF-----------------------------------------
EFR3B_Mus_musculus_26        --------------PDLCV-----------------------------------------
Yarrowia_lipolytica_27       --------------PQTFL-----------------------------------------
EFR3A_Rattus_norvegi_28      --------------PDLCV-----------------------------------------
Gallus_gallus_29             --------------PDLCV-----------------------------------------
Kluyveromyces_lactis_30      ------------------M-----------------------------------------
Ornithorhynchus_anat_31      --------------PDLCV-----------------------------------------
EFR3A_Oryctolagus_cu_32      --------------PDLCV-----------------------------------------
Nasonia_vitripennis_33       --------------PELFV-----------------------------------------
Candida_glabrata_34          --------------AN--------------------------------------------
Ixodes_scapularis_35         --------------PELFV-----------------------------------------
Daphnia_pulex_36             --------------PDLFV-----------------------------------------
Tribolium_castaneum_37       --------------PELFF-----------------------------------------
Anopheles_gambiae_38         --------------PELFF-----------------------------------------
EFR3B_Ciona_intestin_39      --------------PSIYA-----------------------------------------
Tetraodon_nigrovirid_40      --------------PDLCV-----------------------------------------
Solenopsis_invicta_41        --------------PELFV-----------------------------------------

Cryptococcus_neoform_0       -------------SLGR-PVDVSSSS------------------
Drosophila_melanogas_1       -------------Y------------------------------
EFR3A_Equus_caballus_2       -------------Y------------------------------
Neurospora_crassa_3          SDIKSLGAGGKPPY------------------------------
Debaryomyces_hanseni_4       -------------TSD----------IDSILEGLDIDDDSEIIV
Saccharomyces_cerevi_5       --------------------------------------------
Ustilago_maydis_6            -------------TNGTAPISPPYTS------------------
Ascaris_suum_7               -------------Y------------------------------
Harpegnathos_saltato_8       -------------Y------------------------------
Monodelphis_domestic_9       -------------Y------------------------------
Ailuropoda_melanoleu_10      -------------Y------------------------------
Ashbya_gossypii_11           --------------------------------------------
Nematostella_vectens_12      -------------Y------------------------------
Acyrthosiphon_pisum_13       -------------F------------------------------
Culex_quinquefasciat_14      -------------Y------------------------------
Aedes_aegypti_15             -------------Y------------------------------
Camponotus_floridanu_16      -------------Y------------------------------
Caenorhabditis_elega_17      -------------Y------------------------------
EFR3B_Homo_sapiens_18        -------------Y------------------------------
EFR3A_Homo_sapiens_19        -------------Y------------------------------
EFR3B_Ailuropoda_mel_20      -------------Y------------------------------
Schizosaccharomyces__21      --------------------------------------------
EFR3A_Xenopus_laevis_22      -------------Y------------------------------
EFR3A_Taeniopygia_gu_23      -------------Y------------------------------
EFR3B_Danio_rerio_24         -------------Y------------------------------
Anopheles_darlingi_25        -------------Y------------------------------
EFR3B_Mus_musculus_26        -------------Y------------------------------
Yarrowia_lipolytica_27       -------------NTWTVTDNLGGLTV-----------------
EFR3A_Rattus_norvegi_28      -------------Y------------------------------
Gallus_gallus_29             -------------Y------------------------------
Kluyveromyces_lactis_30      -------------V------------------------------
Ornithorhynchus_anat_31      -------------Y------------------------------
```

```
EFR3A_Oryctolagus_cu_32       -------------Y------------------------------
Nasonia_vitripennis_33        -------------Y------------------------------
Candida_glabrata_34           -------------Y------------------------------
Ixodes_scapularis_35          -------------F------------------------------
Daphnia_pulex_36              -------------Y------------------------------
Tribolium_castaneum_37        -------------Y------------------------------
Anopheles_gambiae_38          -------------Y------------------------------
EFR3B_Ciona_intestin_39       -------------Y------------------------------
Tetraodon_nigrovirid_40       -------------Y------------------------------
Solenopsis_invicta_41         -------------Y------------------------------
```

Figure S4. Multispecies protein sequence alignment of EFR3A/B for ConSurf analysis.
