# Supplementary material for: Rare deleterious mutations of the gene EFR3A in autism spectrum disorders
Source: Mol Autism. 2014 Apr 29;5:31. doi: 10.1186/2040-2392-5-31 (PMC4032628; doi:10.1186/2040-2392-5-31)
Supplement: Additional file 16: Table S10 — siRNA sequences used to verify EFR3A antibody specificity. [file 2040-2392-5-31-S16.zip › 2059280318113810_add17.docx]

**Additional references**

1. Roy A, Kucukural A, Zhang Y: **I-TASSER: a unified platform for automated protein structure and function prediction.** *Nat Protoc* 2010, **5:**725–738.
2. Zhang Y: **Template-based modeling and free modeling by I-TASSER in CASP7.** *Proteins* 2007, **69:**108–117.
3. Kelley LA, Sternberg MJE: **Protein structure prediction on the Web: a case study using the Phyre server.** *Nat Protoc* 2009, **4:**363–371.
4. Xu J, Peng J, Zhao F: **Template-based and free modeling by RAPTOR++ in CASP8.** *Proteins* 2009, **77:**133–137.
5. Hildebrand A, Remmert M, Biegert A, Söding J: **Fast and accurate automatic structure prediction with HHpred.** *Proteins* 2009, **77:**128–132.
6. Kippert F, Gerloff DL: **Highly sensitive detection of individual HEAT and ARM repeats with HHpred and COACH.** *PLoS One* 2009, **4:**e7148.
7. Andrade MA, Ponting CP, Gibson TJ, Bork P: **Homology-based method for identification of protein repeats using statistical significance estimates.** *J Mol Biol* 2000, **298:**521–537.
